# Synthetic High‐Throughput Microarrays of Peptidoglycan Fragments as a Novel Sero‐Diagnostic Tool for Patient Antibody Profiling

**DOI:** 10.1002/anie.202420874

**Published:** 2025-02-28

**Authors:** Alexandra Tsouka, Yanyan Fu, Manuel G. Ricardo, Peter H. Seeberger, Yue Wang, Gerald B. Pier, Detlef Schuppan, Louis Boon, Jan Maarten van Dijl, Maria C. Bolling, Girbe Buist, Felix F. Loeffler, Jon D. Laman

**Affiliations:** ^1^ Department of Biomolecular Systems Max Planck Institute of Colloids and Interfaces 14476 Potsdam Germany; ^2^ Department of Medical Microbiology and Infection Prevention University of Groningen University Medical Center Groningen Groningen The Netherlands; ^3^ Institute of Chemistry and Biochemistry Freie Universität Berlin 14195 Berlin Germany; ^4^ A*STAR Infectious Diseases Labs Agency for Science and Technology Research (A*STAR) Singapore Singapore 138648; ^5^ Mass General Brigham Harvard Medical School Boston MA 02115 USA; ^6^ Institute of Translational Immunology and Celiac Center Medical Center Johannes-Gutenberg University 55099 Mainz Germany; ^7^ Division of Gastroenterology Beth Israel Deaconess Medical Center Harvard Medical School Boston MA 02115 USA; ^8^ JJP Biologics 00-728 Warsaw Poland; ^9^ Department of Dermatology UMCG Center of Expertise for Blistering Diseases University Medical Center Groningen The Netherlands; ^10^ Department of Pathology and Medical Biology University of Groningen University Medical Center Groningen Groningen The Netherlands

**Keywords:** laser-induced forward transfer (LIFT), solid phase synthesis, autoimmunity, epidermolysis bullosa (EB), antibodies

## Abstract

Peptidoglycan (PGN) is a complex biopolymer crucial for cell wall integrity and function of all bacterial species. While the strong inflammatory properties of PGN and its derived muropeptides are well‐documented in human innate immune responses, adaptive immunity, including antibody responses to PGN, remain inadequately characterized. Microarray technology represents a cost‐ and time‐efficient method for studying such interactions. Our laser‐based technology enables the high‐throughput synthesis of biomolecules on functionalized glass slides. Here, this on‐chip synthesis was developed for PGN fragments, to generate a variety of 216 stem peptides and attach six different glycan moieties that are major structural components of bacterial cell walls. Thereby, 864 PGN fragments from different Gram‐negative and Gram‐positive species were generated. The arrays were validated with four different monoclonal antibodies against PGN or poly‐*N*‐acetyl glucosamine and identified their epitopes. Finally, proof of concept for antibody profiling in patient samples was performed by comparing a panel of well‐characterized plasma samples of epidermolysis bullosa (EB) patients suffering from (chronic) wounds with Staphylococcus aureus infection. EB patients show an increased response to the muramyl dipeptide. Therefore, this novel high‐throughput PGN glycopeptide microarray technology promises to identify distinct antibody profiles against human microbiomes in diseases, notably in those involving the intestine.

## Graphical abstract

Using a novel on‐chip synthesis technology, microarrays of peptidoglycan fragments were generated comprising 864 variants derived from Gram‐positive and Gram‐negative bacteria. These were used to map monoclonal antibody specificity and to screen samples from patients with the autoimmune disease epidermolysis bullosa, revealing distinct antibody responses. This highlights the platform's potential for profiling microbiome‐associated diseases.

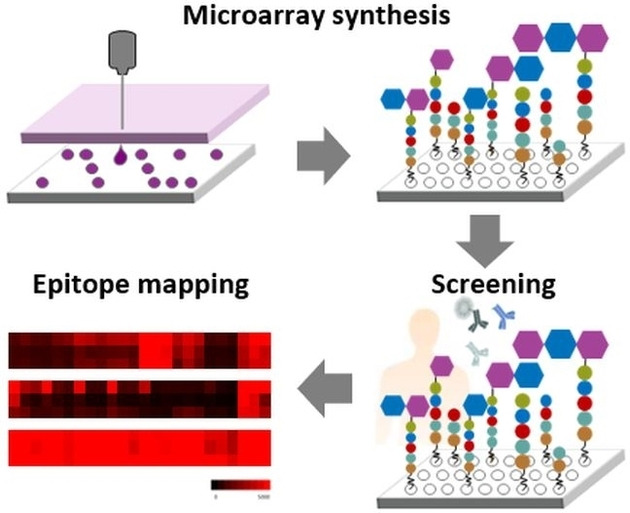

## Introduction

**Figure 1:**
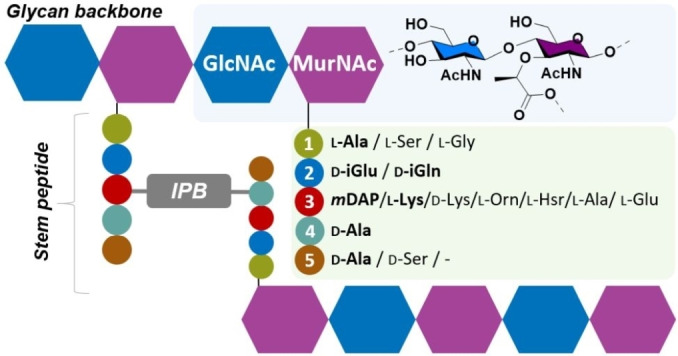
Peptidoglycan structure and sequence variation of the stem peptide. The different glycan strands are branching from the MurNAc residues with the stem peptides. The length of the stem peptides can be up to 5AA long, while the interpeptide bridge (IPB), connecting the two stem peptides may contain up to seven AA, typically polyglycine. l/d‐Ala: l/d‐alanine; l‐Arg: l‐arginine; l‐Cit: l‐citrulline; l‐Gly: l‐glycine; l‐Glu: l‐glutamic acid; d‐iGlu: d‐isoglutamic acid, l/d‐iGln: l/d‐isoglutamine; l‐Hsr: l‐homoserine; l/d‐Lys: l/d‐lysine; mDAP: meso‐diaminopimelic acid; l‐Orn: l‐ornithine; l/d‐Ser: l/d‐serine.

Peptidoglycans (PGNs) are large rigid biopolymers present in the cell wall of all bacteria. They are essential for bacterial survival, as they serve as an integral structural component and protective barrier, helping to maintain osmotic pressure and cell wall shape.^[1]^ They consist of a stable glycan backbone cross‐linked to a short and variable peptide chain, the so‐called stem peptide (Figure 1). The glycan backbone is composed of two alternating monosaccharide units, *N*‐acetylglucosamine (GlcNAc) and *N*‐acetylmuramic acid (MurNAc), linked together by a β‐1,4 glycosidic bond.^[2]^ In contrast, the stem peptides exhibit a great degree of diversity, incorporating different combinations of l‐ and d‐amino acids (AAs), up to five residues long. The archetypical pentapeptide stem typically contains an l‐alanine (l‐Ala) and two d‐alanine (d‐Ala) residues at positions 1, 4, and 5, while the amino acids in positions 2 and 3 are different for Gram‐positive and Gram‐negative bacteria. Specifically, d‐isoglutamine (d‐iGln) and l‐lysine (l‐Lys) are found in Gram‐positive bacteria, while d‐isoglutamic acid (d‐iGlu) and meso‐diaminopimelic acid (mDAP) are present in Gram‐negative bacteria. The peptide stem chains may be crossed‐linked to neighboring peptide chains by a short interpeptide bridge, forming a 3D mesh‐like structure. This bridge can be several amino acids long, as exemplified by the pentaglycine bridge in *Staphylococcus aureus*.[^3^, ^4^] The glycan backbone and the peptide stem are connected through an amide bond between the d‐lactoyl moiety of the MurNAc and the l‐Ala.^[5]^ Different modifications of the glycan backbone occur, such as *O*‐acetylation, *N*‐deacetylation, or phosphorylation. Together with specific amino acid variants, this results in over 10.000 distinct small PGN structure variants (see Supporting Information, *Section C*, for calculation of estimation).[^6^, ^7^, ^8^]

The structural diversity of these PGN variants^[6]^ and the fact that their roles in multiple physiological and biological processes, such as inflammation, immune response, and host interactions is not well understood, make them an important target for cellular and molecular studies. Recent research mainly concentrated on their role as antibiotic^[9]^ and other therapeutic targets in metabolic and mental health disorders,^[10]^ as immunostimulatory activators,^[11]^ but also on their involvement in brain development and function,^[12]^ and in chronic inflammatory (autoimmune) diseases.[^11^, ^13^, ^14^] In particular, PGN fragments have been linked to rheumatoid arthritis,^[15]^ Lyme disease,^[14]^ Alzheimer's disease,[^16^, ^17^, ^18^] and multiple sclerosis.[^11^, ^19^, ^20^, ^21^] Due to their large variety representing a muropeptidome, it is currently difficult to study this vast landscape of PGN structures, which can have different physiological as well as pathogenic functions.[^22^, ^23^]

Microarray technology allows for the high‐throughput screening of biomolecules. In situ chemical synthesis of biomolecules in the array format has significantly accelerated microarray production from months or years to a few weeks and has enabled many applications in immunological and biomedical research.[^24^, ^25^, ^26^, ^27^, ^28^, ^29^, ^30^] Thereby, rationally designed antigenic variants can be quickly accessed in a cost‐ and time‐efficient manner. In particular, peptide microarrays are widely used for the study of antibody interactions such as mAbs or patient samples, with linear epitopes, via direct incubation and fluorescence scan analysis.^[30]^

Previous PGN studies used different strategies for their synthesis, including solution and solid phase synthesis, as well as chemical and chemoenzymatic approaches.[^31^, ^32^, ^33^, ^34^] We used automated glycan assembly (AGA) to synthesize ten oligosaccharide fragments of the PGN structure.^[35]^ However, these methods produce only one single molecule at a time, highlighting the need for parallel synthesis of many PGN variants to rapidly screen and identify potential PGN epitopes and disease biomarkers.

Here, we report the synthesis of many different PGN cell wall fragments representing a variety of bacterial species, directly in the microarray format. The microarray synthesis of different stem peptides was achieved using the laser‐induced forward transfer (LIFT) technology,^[36]^ while attachment of the respective glycan variants was accomplished via an amide bond directly on the solid support. Since stem peptides can contain d‐ and l‐, as well as uncommon amino acids, their synthesis needed to be optimized. This enabled us to generate complex PGN microarrays, comprising 864 PGN fragment molecules. The synthesized arrays were used for epitope mapping of four different monoclonal antibodies, and for screening of plasma samples from patients with the genetic skin fragility disorder epidermolysis bullosa (EB), who usually have many *S. aureus* colonized wounds. It has been shown that EB patients carry multiple different *S. aureus* strains in their wounds, eliciting a pronounced antibody response against *S. aureus* surface proteins.[^37^, ^38^]

### Results and Discussion

#### Synthesis of PGN Arrays and Validation by four Monoclonal Antibodies

**Figure 2:**
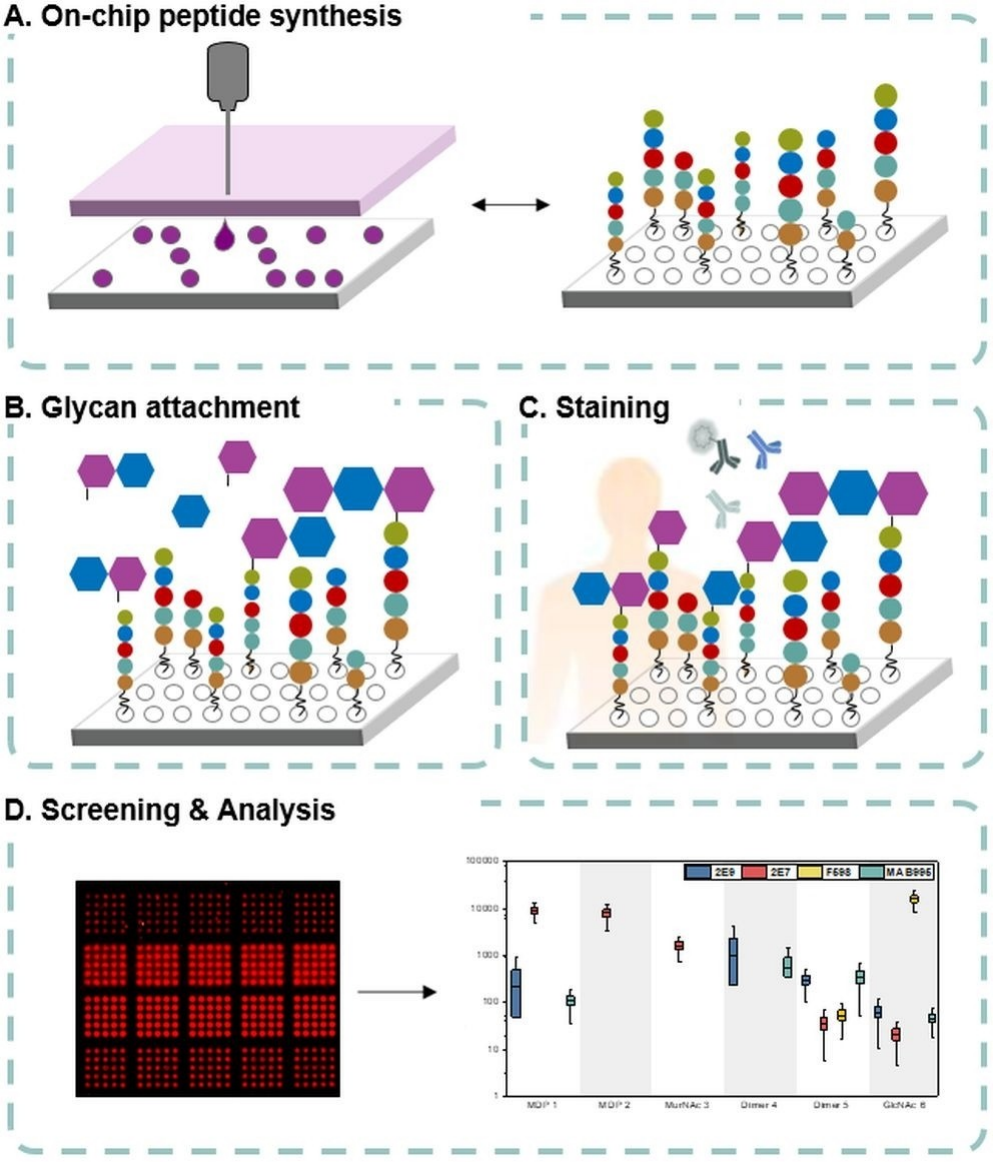
PGN microarray synthesis and screening. A) Synthesis of peptide microarrays via LIFT. B) Attachment of glycan molecules. C) Antibody signal after plasma incubation. D) Fluorescence scan and analysis of fluorescence intensities.

To generate the microarrays for this antibody screening study, the on‐chip automated laser‐based microarray synthesis technology was applied, using LIFT.^[26]^ In brief, the technology enables the parallel solid‐phase synthesis of biomolecules, such as peptides and glycans, by successively printing and coupling minute amounts of different building blocks onto a microarray surface (Figure 2).[^26^, ^28^, ^29^, ^39^] The desired chemical building blocks are embedded in a polymer matrix which is spin‐coated on a donor film. Similar to a typewriter, the different building blocks are patterned onto a functionalized substrate by laser irradiation. Then, the building block pattern is coupled to the microarray surface in a 10 min oven step at 95 °C, followed by washing, capping, and deprotection steps. Printing and processing steps are repeated, until the desired structures on the array are formed. Monoclonal antibody and patient plasma interactions are screened via fluorescence scan with fluorescently labelled secondary antibodies.

**Figure 3:**
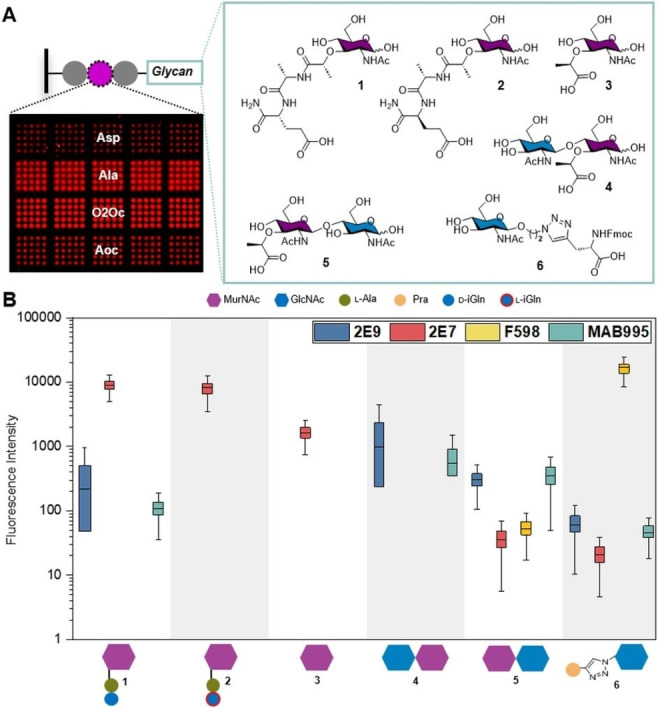
Generation of PGN fragment arrays on different surface linker functionalizations for epitope mapping of monoclonal antibodies. A) Surface functionalization by l‐aspartic acid (l‐Asp), l‐alanine (l‐Ala), 3,6‐dioxaoctanoic acid (8O_2_Oc), and 8‐aminooctanoic acid (8Aoc), on the commercial PEGMA/MMA‐β‐Ala functionalized solid support and attachment of the glycan moieties via amide bond formation. B) Representative analysis of the fluorescence intensities on l‐Asp array functionalization after incubation with mouse anti‐PGN monoclonal 2E9, mouse anti‐PGN monoclonal 2E7, mouse anti‐PGN monoclonal MAB995, and human anti‐PNAG monoclonal F598. Detection was achieved with goat anti‐mouse IgG polyclonal and goat anti‐human IgG polyclonal antibodies respectively. Box plots (center line, median; box limits, upper and lower quartiles; whiskers, outermost data point that falls within 1.5×interquartile range) were calculated from 125 spots for each linker pre‐functionalization (Supporting Information).

First, we validated the possibility of PGN synthesis on the array format by solution synthesis of a muramyl dipeptide (MDP)‐like structure on a glass slide without side reactions, and solution synthesis of a pentapeptide disaccharide (Figure 2B, Supporting Information, *Section D*). Then, we investigated the impact of the surface/substrate functionalization on the antibody binding by transferring different linkers/spacers on commercial β‐Ala‐PEGMA/MMA functionalized solid supports. Four Fmoc‐protected building blocks, l‐Aspartic acid (l‐Asp), l‐Alanine (l‐Ala), hydrophilic 3,6‐dioxaoctanoic acid (8O_2_Oc), and hydrophobic 8‐aminooctanoic acid (8Aoc), were explored as potential spacers, by adjacently patterning each as five times 5×5 spot replicas (= 125 spots) (Figure 3). The surface was further processed, and an Fmoc‐protected β‐Ala was coupled from solution onto the 5×5×5 spot patterned solid support. After deprotection of the temporary protecting group, the free amino groups were coupled with the glycan molecules **1–5** via their respective carboxylic acid groups (Figure 3). Coupling was either performed in a one‐step reaction, forming an amide bond, or in a two‐step reaction for molecule **6**, by first attaching an l‐propargylglycine (Pra) and then a GlcNAc azide via copper (I)‐catalyzed azide‐alkyne cycloaddition reaction (CuAAC) (Supporting Information *Section E*).[^28^, ^39^]

Building blocks **1–6** were either synthesized according to established protocols^[35]^ or prepared from commercially available precursors (Figure 3A, Supporting Information, *Sections F*). Muramyl dipeptide (MDP, MurNAc‐l‐Ala‐d‐iGln) **1** was implemented since it is the smallest bioactive PGN motif present in all bacteria,^[40]^ while the l‐analogue MDP **2** (l‐iGln instead of d‐iGln), served as negative control. Building blocks **3–6** represent different glycan backbone fragments of PGN which were attached to an artificial β‐Ala‐X or Pra‐β‐Ala‐X linker, where X is one of the four mentioned AA functionalizations. Two sets of array types were generated: one was treated with a trifluoroacetic acid (TFA) deprotection solution for the removal of acid‐labile protecting groups, while the other was used directly without additional treatment. Both array types were incubated with three known monoclonal mouse anti‐PGN antibodies (mAbs), namely 2E9,[^21^, ^41^] 2E7,^[13]^ and MAB995,^[18]^ as well as with the known monoclonal human anti‐poly‐β‐1,6‐*N*‐acetylglucosamine (anti‐PNAG) antibody F598.[^42^, ^43^] Bound anti‐PGN and anti‐PNAG mAbs were stained with fluorescently labeled anti‐mouse‐IgG or anti‐human‐IgG Fc‐fragment specific secondary antibodies (Figure 3B).

Acetylated arrays without the glycan‐containing molecules served as background and negative control. The fluorescence analysis of the TFA‐treated arrays on the Asp‐functionalized solid support (Figure 3B) demonstrated that the 2E7 antibody exhibited robust and selective binding to all arrays bearing a MurNAc monomer (**1**, **2**, **3**). Additionally, a weaker but discernible interaction was observed in the dimer **5** with a terminal MurNAc, as well as the GlcNAc **6**. In contrast, mAbs 2E9 and MAB995, showing a similar binding behavior, bound selectively to MDP **1**, but not to the control MDP **2** or to MurNAc **3**. This highlights the importance of the amino acids in positions 1 and 2 of the PGN stem peptides. In addition, 2E9 and MAB995 bound to both dimers (**4**, **5**), indicating a second selectivity for the glycan backbone. Finally, mAb F598 showed a very different binding profile with high specificity for GlcNAc **6** only and a weak signal for dimer **5**. Generally higher fluorescence intensities could be observed on arrays without TFA treatment, yet the binding trends remained consistent. This phenomenon was predominantly observed on arrays with d‐lactoyl moieties, which likely have undergone hydrolytic cleavage. This cleavage was not observed with GlcNAc moieties due to the CuAAC attachment, which was confirmed for both MAB995 and F598 (Supporting Information *Section H*).

### Optimization and Generation of Combinatorial PGN Arrays

**Figure 4:**
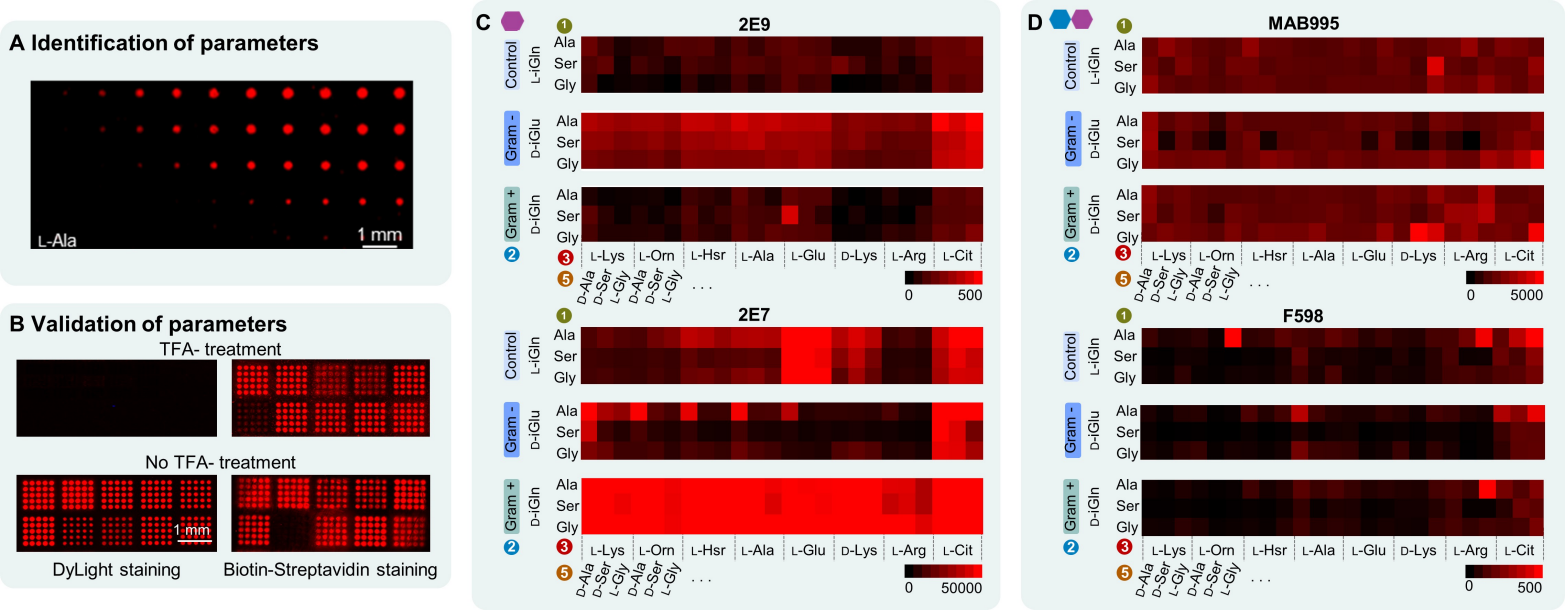
Generation of combinatorial PGN microarrays. A) Identification of lasing parameters and quantification of AA coupling by fluorescence labeling of amino groups. Example fluorescence (DyLight) staining of l‐Ala pattern with increasing laser parameters. B) Validation of the conditions and visualization by direct and indirect staining of l‐Ala, d‐Ala, l‐Lys, l‐Orn, d‐iGln (top five AAs, left to right), d‐iGlu, l‐Gln, l‐Ser, l‐iGln, d‐Ser (bottom five AAs, left to right). C, D) Optimal conditions were used for the generation of PGN arrays. Heat map of the array screening of 216 pentapeptide variations bearing the respective glycan moieties: C) MurNAc **3** and D) dimer **4**, incubated with monoclonal mouse anti‐PGN 2E9, 2E7, MAB995, and monoclonal human anti‐PNAG F598. Detection with goat anti‐mouse IgG polyclonal and goat anti‐human IgG polyclonal antibodies respectively. Fluorescence intensity was determined as the median of three spot replicas.

Next, we generated microarrays on β‐Ala‐Asp functionalized solid supports, displaying larger parts of the PGNs with combinatorial variants of the stem peptide. Since the laser‐based approach has been developed only for the 20 natural l‐AAs, we identified and validated the laser and synthesis parameters (Figure 4 A, B) for the five different d‐AAs (d‐iGlu, d‐iGln, d‐Lys, d‐Ala, d‐Ser) and four special l‐AAs (l‐iGln, l‐Orn, l‐Hsr, l‐Cit), before the generation of the combinatorial PGN arrays. Therefore, we followed an established optimization pipeline.^[26]^ Fluorescence staining was used to quantify the quality of the generated AA spots (depending on laser‐transfer parameters, spot size, homogeneity, and yield), either by direct DyLight 633 *N*‐hydroxysuccinimide ester labeling or two‐step biotin‐streptavidin labeling of the N‐terminus. Screening of the results was performed with and without TFA side chain deprotection for both labeling techniques. As previously reported, the observed fluorescence intensity was indicative of synthesis yield/quality.[^26^, ^29^] We successfully identified and validated laser parameters and synthesis conditions (Supporting Information *Section I*).

Afterwards, we generated fully combinatorial high density PGN arrays including 216 different stem‐pentapeptide variant fragments as three replicas (for pattern see Supporting Information, *Section J*). The generated pentapeptides contained all the variants shown in Figure 1 as well as additional pentapeptides that can be formed after post traditional modifications. For the first AA position, the three variants l‐Ala, l‐Ser, and l‐Gly were implemented. In position 2, d‐iGln, d‐iGlu, and l‐iGln were included, representing the Gram‐positive, Gram‐negative, and negative control (see control MDP **2**) of the peptide stems respectively. In position 3, l‐Lys, l‐Orn, l‐Hsr, l‐Ala, l‐Glu, d‐Lys, l‐Arg, and l‐Cit were used. The latter two were included as part of common post‐transitional modifications related with autoimmune diseases such as rheumatoid arthritis, as l‐Orn can be converted to l‐Arg, and then subsequently to l‐Cit.^[44]^ Position 4 was always kept constant with a d‐Ala, whereas position 5 was varied between d‐Ala, d‐Ser, and l‐Gly. Finally, sugar moieties **3–5** were attached onto the synthesized peptide arrays via amide bond formation. Then, these arrays were used to screen the respective responses of the three anti‐PGN and the anti‐PNAG mAbs (Figure 4C, D, and Supporting Information, *Section J*). The results showed a strong binding dependency of the mAbs either on the glycan moiety or on the AAs, especially at positions 2 and 3 of the peptide stem, where at position 2 the d‐iGln and d‐iGlu distinguish between Gram‐positive and Gram‐negative bacteria.

The mAbs 2E9 and 2E7 exhibited a higher degree of selectivity for the stem peptide variants (Figure 4C, Supporting Information, *Section J*). In particular, 2E9 bound to structures with MurNAc **3**, with a strong preference for d‐iGlu in position 2 (Gram‐negative strains), while weak/unspecific binding for d‐iGln (Gram‐positive) and l‐iGln (control) was observed. These results validate our initial findings (Figure 3B). In position 3, the lowest intensities were observed for l‐Lys, which is exclusively found in Gram‐positive strains, supporting the finding of selectivity for Gram‐negative strains. Conversely, the highest intensities were detected for l‐Cit in position 3. Similar signals for 2E9 were also observed on the combinatorial arrays with dimers **4** and **5**, with dimer **4** exhibiting greater intensity than **5**.

In contrast, mAb 2E7 demonstrated a clear affinity towards structures containing d‐iGln in position 2 (Figure 4C) and l‐Lys in position 3, indicating a specificity for Gram‐positive strains. The impact of AAs in positions 1, 3, and 5 is much less pronounced. The AA in position 1 does not exhibit significant impact, fluctuating from l‐Gly to l‐Ser and then l‐Ala. Additionally, the majority of AAs in position 3 presented high intensities, with the exception of l‐Orn, l‐Lys, and l‐Arg, exhibiting relatively reduced intensities compared with the rest of the AAs, as has been previously demonstrated. The final amino acid in position 5 does not significantly impact the binding trend. However, there is a discernible change in intensity, with d‐Ala (in most bacteria) exhibiting the highest signal, followed by d‐Ser and l‐Gly. Identical binding trends were observed on the combinatorial arrays with dimers **4** and **5**, with twice the intensity and apparently even higher specificity (Supporting Information, *Section J*).

Finally, the binding of mAb MAB995 and F598 demonstrated no notable impact from the stem peptide on antibody recognition. These findings indicate that antibody recognition is primarily influenced by the oligosaccharide and its length, rather than the stem peptide variation. These findings are consistent with those of the previous experiments (Figure 3B) for both antibodies, whereas no binding was detected on the arrays functionalized with structures **1–3**. Nevertheless, in the case of MAB995, binding was detected in all arrays, whereas for F598, binding was only identified on arrays with dimer **4**, in contrast to the previous experiment (Figure 3B vs. 4D, Supporting Information, *Section J)*. The selective binding of F598 was validated by additional PGN oligosaccharide fragment^[35]^ array (Supporting Information, *Section K)*. For future investigations, longer oligosaccharide structures should be synthesized and screened.

### Proof of Concept Screening of Epidermolysis Bullosa Patient Samples

Encouraged by the successful validation of the microarrays by screening of the four anti‐PGN mAbs and mapping of their respective epitopes, we next sought to provide proof of concept of our initial arrays for IgG reactivity against PGN structures in plasma samples from patients with EB, which is a rare genetic skin disorder that leads to blistering and erosions from minor trauma or friction. The wounds of EB patients are highly colonized with *S. aureus* and are more exposed to staphylococcal antigens than healthy individuals. This appears to elicit an increased immune response towards *S. aureus*.[^37^, ^38^] We screened 13 EB patient samples, 8 healthy carriers (C) of *S. aureus*, and 11 healthy non‐carriers (NC).

The EB samples included seven patients with junctional EB (JEB*), in whom besides skin fragility from birth, wound healing is also impaired and wound heal with scarring, one patient with EB simplex (EBS +), in whom blisters are caused by heat or friction and heal relatively easily without scarring, and five patients with dystrophic EB (DEB −), in whom blisters heal with scarring, and usually later in life chronic wounds persist.

The arrays bearing the four β‐Ala‐X functionalizations with glycan moieties **1–4** and **6**, were subjected to TFA treatment and then incubated with the corresponding diluted human plasma samples overnight at 4 °C. Secondary staining was performed with anti‐human IgG Fc DyLight 650 and anti‐human IgA rhodamine (TRIC) in parallel. Since the results of the IgA staining were less conclusive, they are presented in the Supporting Information (*Section L*).

Nine out of the 13 EB patient samples contained IgG binding to MDP **1**. Notably, EB01, EB15, EB51, EB53, EB58 and EB60 showed a faded IgG response, indicating a weaker immune response towards PGN compared to other samples. In contrast, samples from patients EB02, EB11 and EB14 showed intense IgG signals, indicating a robust immune response to MDP, most likely reflecting higher levels of MDP‐specific antibodies in these individuals. These results highlight the variability in immune responses to MDP among EB patients, which may be related to disease severity or subtype, or the level of wound colonization by *S. aureus*. However, larger patient and control sample numbers have to be analyzed in the future, to validate trends. Interestingly, healthy controls NC01 and NC02 also demonstrated IgG responses to MDP, as well as nasal carrier C15.

**Figure 5:**
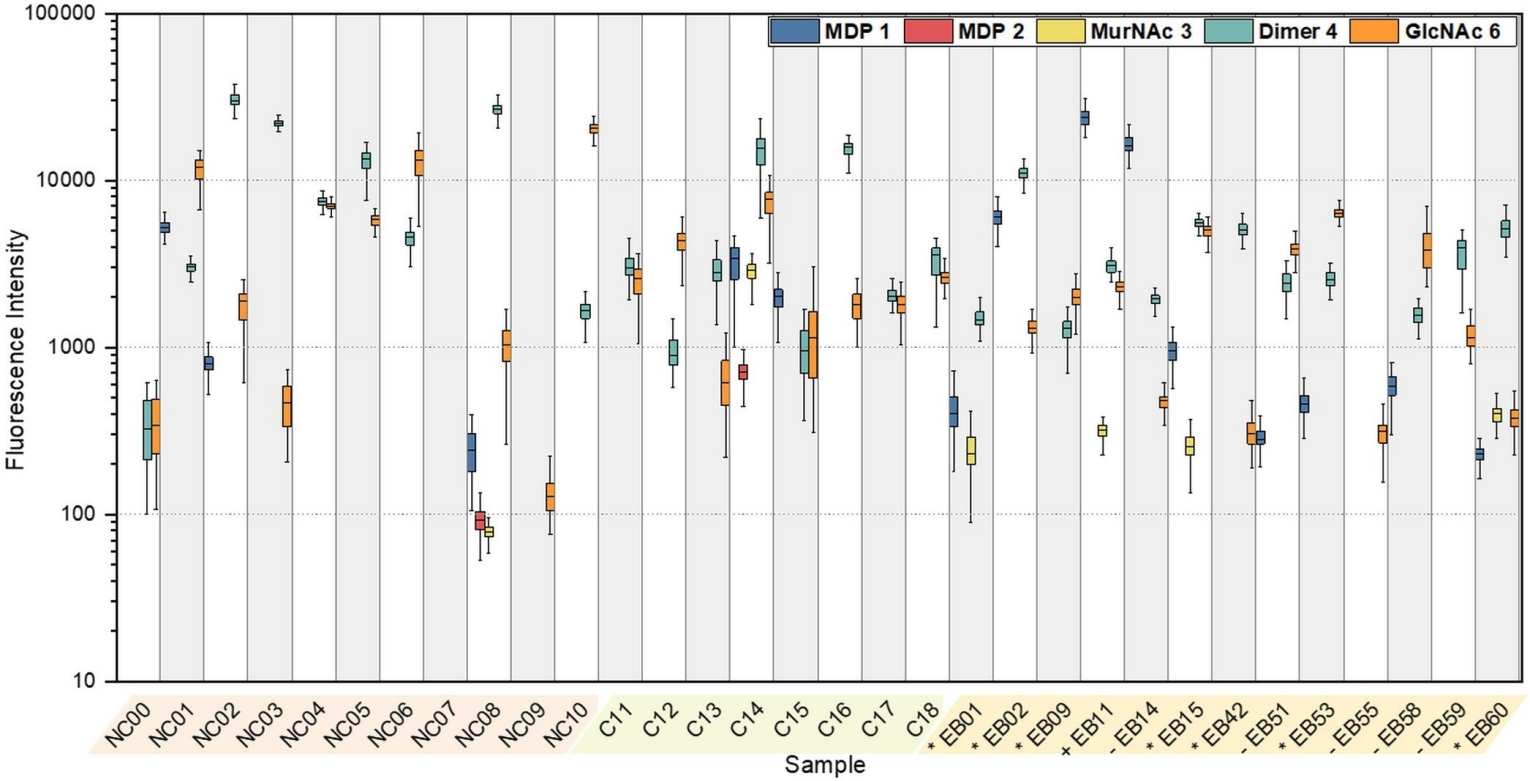
IgG response from healthy non‐carriers (NC), healthy carriers (C) of *S. aureus*, and epidermolysis bullosa (EB) patients on β‐Ala‐l‐Asp surface functionalization. EB sample groups: JEB (*), DEB (−), EBS sample (+). Detection was achieved with a fluorescently labelled goat anti‐human IgG polyclonal antibody. Box plots (center line, median; box limits, upper and lower quartiles; whiskers, outermost data point that falls within 1.5×interquartile range) were calculated from 125 spots.

Specific IgG binding to MurNAc **3** functionalized arrays (Figure 5) was observed in seven out of 13 EB patient samples. In particular, patients with JEB (EB01, EB15, EB60) exhibited an IgG response across all four tested surface functionalizations (l‐Asp, l‐Ala, 8O_2_Oc, 8Aoc, Supporting Information *Section L*). In contrast, EBS patient EB11 without chronic wounds showed an exclusive IgG response on the Asp functionalization. Interestingly, EB51 and EB55 samples did not display any MurNAc‐IgG response on the β‐Ala‐l‐Asp functionalization, whereas responses were detected on the other three functionalizations (Supporting Information *Section L*). The EB14 sample only showed a MurNAc‐IgG response on functionalizations with 8O_2_Oc and 8Aoc These results could suggest that specific surface functionalization of PGN plays a critical role in the IgG responses of patients with different EB subtypes, or that a previously unnoticed EB subtype‐specific selectivity for wound colonization by particular *S. aureus* lineages exists, highlighting the importance of tailored approaches to antibody screening in disease etiology.

All plasma samples, except NC09 and EB55, showed a high IgG response against dimer **5**. Similarly, all samples, except EB01 showed binding to GlcNAc **6**. The response was higher in healthy individuals than in patients. Plasma samples NC08 and C14 showed an IgG reactivity across all structures, but not in the negative control condition. These results may be at least partially attributable to contamination with lipids during sample acquisition, affecting assay performance. No binding was observed on the plain arrays without glycan moieties, which were used as a negative control, and neither for NC07, which may potentially be attributed to hemolysis or an infection.

## Conclusion

In summary, our automated high‐throughput LIFT synthesizer was employed for the generation of arrays comprising PGN fragments of stem peptides and sugars with high spot density and precision. The initial objective was to investigate the impact of surface functionalization on the recognition of PGN glycan fragments by three anti‐PGN mAbs and one anti‐PNAG mAb.

Subsequently, a pipeline was used to optimize the laser‐based synthesis of PGN stem peptide microarrays with the required AA building blocks. Different staining protocols were employed to validate the precision, reproducibility, spot size, and morphology for each structure. Subsequently, combinatorial PGN arrays, comprising 216 distinct pentapeptide variants were generated via LIFT, and glycans **3–5** were immobilized via amide bond formation, enabling the epitope and specificity mapping of four anti‐PGN/PNAG mAbs. The mAb 2E9 was raised against an unknown bacterial strain,^[41]^ and our results show its preference for Gram‐negative bacterial structures. In contrast, mAb 2E7 was raised against MDP,^[13]^ a part of Gram‐positive PGN structures, which we could confirm in our epitope mapping. In addition, we investigated IgG reactivity in plasma from *S. aureus* carriers, non‐carriers, and patients with EB by employing glycan‐based PGN fragment arrays with different functionalizations. Our findings indicate that the surface functionalization influences the IgG recognition, which may be associated with the various subtypes of the disease. Further investigations are necessary to confirm potential previous bacterial infections or a cross‐reactivity to MDP **1** in EB and NC plasma samples. In addition, IgG subclass reactivity should be investigated. The same approach should be implemented in samples of diseases where large mucosal surfaces are in contact with a complex microbiome, such as the gastrointestinal tract. Here, also IgA or IgM antibody titers may reveal important diagnostic or prognostic information. Moreover, the generation of more complex PGN arrays with longer glycan moieties is essential to further investigate the specificities of MAB995 and F598, and to expand the diagnostic portfolio of PGN antibody testing in patients. Finally, the synthesis and implementation of *m*DAP on the arrays should be investigated.

## Conflict of Interests

F.F.L. is named on a patent application related to laser‐based microarray synthesis. P.H.S. declares a significant financial interest in GlycoUniverse GmbH & Co. KGaA, the company that commercialized AGA synthesis instruments, building blocks, and other reagents. All other authors declare that they have no competing interests.

1

## Supporting information

As a service to our authors and readers, this journal provides supporting information supplied by the authors. Such materials are peer reviewed and may be re‐organized for online delivery, but are not copy‐edited or typeset. Technical support issues arising from supporting information (other than missing files) should be addressed to the authors.

Supporting Information

## Data Availability

The data that support the findings of this study are available in the supplementary material of this article.

## References

[1] K. H. Schleifer , O. Kandler , Bacteriol. Rev. 1972, 36, 407–477. 4568761 10.1128/br.36.4.407-477.1972 PMC408328

[2] M. F. Dwayne C. Savage, *Bacterial Adhesion*, Springer US, Boston, MA, **1985**.

[3] R. Wheeler , I. Gomperts Boneca , Gut Microbes 2024, 16, 2395099. 39239828 10.1080/19490976.2024.2395099 PMC11382707

[4] M. Wang , G. Buist , J. M. van Dijl , FEMS Microbiol. Rev. 2022, 46, 1–19. 10.1093/femsre/fuac025 PMC9616470 35675307

[5] S. Garde , P. K. Chodisetti , M. Reddy , EcoSal Plus 2021, 9, 1–35. 10.1128/ecosalplus.ESP-0010-2020 PMC11168573 33470191

[6] A. K. Yadav , A. Espaillat , F. Cava , Front. Microbiol. 2018, 9, 1–9. 30233540 10.3389/fmicb.2018.02064 PMC6127315

[7] W. Vollmer , D. Blanot , M. A. De Pedro , FEMS Microbiol. Rev. 2008, 32, 149–167. 18194336 10.1111/j.1574-6976.2007.00094.x

[8] P. J. Moynihan , D. Sychantha , A. J. Clarke , Bioorg. Chem. 2014, 54, 44–50. 24769153 10.1016/j.bioorg.2014.03.010

[9] M. A. Kohanski , D. J. Dwyer , J. J. Collins , Nat. Rev. Microbiol. 2010, 8, 423–435. 20440275 10.1038/nrmicro2333 PMC2896384

[10] K. Berding , J. F. Cryan , Curr. Opin. Psychiatry 2022, 35, 3–9. 34750307 10.1097/YCO.0000000000000758 PMC8654258

[11] J. D. Laman , B. A.'t Hart , C. Power , R. Dziarski , Trends Mol. Med. 2020, 26, 670–682. 32589935 10.1016/j.molmed.2019.11.006

[12] A. Adamantidis , Science 2022, 376, 248–249. 35420955 10.1126/science.abo7933

[13] Z. Huang , J. Wang , X. Xu , H. Wang , Y. Qiao , W. C. Chu , S. Xu , L. Chai , F. Cottier , N. Pavelka , M. Oosting , L. A. B. Joosten , M. Netea , C. Y. L. Ng , K. P. Leong , P. Kundu , K.-P. Lam , S. Pettersson , Y. Wang , Nat. Microbiol. 2019, 4, 766–773. 30833732 10.1038/s41564-019-0381-1

[14] B. L. Jutras , R. B. Lochhead , Z. A. Kloos , J. Biboy , K. Strle , C. J. Booth , S. K. Govers , J. Gray , P. Schumann , W. Vollmer , L. K. Bockenstedt , A. C. Steere , C. Jacobs-Wagner , Proc. Natl. Acad. Sci. USA 2019, 116, 13498–13507. 31209025 10.1073/pnas.1904170116 PMC6613144

[15] I. A. Schrijver , Y. A. De Man , M. J. Melief , J. M. Van Laar , H. M. Markusse , I. S. Klasen , M. P. Hazenberg , J. D. Laman , Clin. Exp. Immunol. 2001, 123, 140–146. 11168011 10.1046/j.1365-2249.2001.01419.x PMC1905964

[16] Y. Chen , S. M. Vargas , T. C. Smith , S. L. R. Karna , T. MacMackin Ingle , K. L. Wozniak , F. L. Wormley , J. Seshu , PLoS Pathog. 2021, 17, e1009535. 33882111 10.1371/journal.ppat.1009535 PMC8092773

[17] M. M. Davis , A. M. Brock , T. G. DeHart , B. P. Boribong , K. Lee , M. E. McClune , Y. Chang , N. Cramer , J. Liu , C. N. Jones , B. L. Jutras , PLoS Pathog. 2021, 17, e1009546. 33984073 10.1371/journal.ppat.1009546 PMC8118282

[18] J. Miklossy , J. Alzheimer′s Dis. 2008, 13, 381–391. 18487847 10.3233/jad-2008-13404

[19] I. A. Schrijver , Y. A. De Man , M.-J. Melief , J. M. Van Laar , H. M. Markusse , I. S. Klasen , M. P. Hazenberg , J. D. Laman , Clin. Exp. Immunol. 2001, 123, 140–146. 11168011 10.1046/j.1365-2249.2001.01419.x PMC1905964

[20] L. Visser , H. Jan de Heer , L. A. Boven , D. van Riel , M. van Meurs , M.-J. Melief , U. Zähringer , J. van Strijp , B. N. Lambrecht , E. E. Nieuwenhuis , J. D. Laman , J. Immunol. 2005, 174, 808–816. 15634902 10.4049/jimmunol.174.2.808

[21] W. G. Branton , J. Q. Lu , M. G. Surette , R. A. Holt , J. Lind , J. D. Laman , C. Power , Sci. Rep. 2016, 6, 37344. 27892518 10.1038/srep37344 PMC5125007

[22] T. Arentsen , Y. Qian , S. Gkotzis , T. Femenia , T. Wang , K. Udekwu , H. Forssberg , R. Diaz Heijtz , Mol. Psychiatry 2017, 22, 257–266. 27843150 10.1038/mp.2016.182 PMC5285465

[23] I. Gabanyi , G. Lepousez , R. Wheeler , A. Vieites-Prado , A. Nissant , G. Chevalier , S. Wagner , C. Moigneu , S. Dulauroy , S. Hicham , B. Polomack , F. Verny , P. Rosenstiel , N. Renier , I. G. Boneca , G. Eberl , P.-M. Lledo , Science 2022, 376, DOI 10.1126/science.abj3986. 35420957

[24] L. C. Szymczak , H.-Y. Kuo , M. Mrksich , Anal. Chem. 2018, 90, 266–282. 29135227 10.1021/acs.analchem.7b04380 PMC6526727

[25] D. S. Mattes , N. Jung , L. K. Weber , S. Bräse , F. Breitling , Adv. Mater. 2019, 31, 1806656. 10.1002/adma.201806656 31033052

[26] G. Paris , J. Heidepriem , A. Tsouka , Y. Liu , D. S. Mattes , S. Pinzón Martín , P. Dallabernardina , M. Mende , C. Lindner , R. Wawrzinek , C. Rademacher , P. H. Seeberger , F. Breitling , F. R. Bischoff , T. Wolf , F. F. Loeffler , Adv. Mater. 2022, 34, 2200359. 10.1002/adma.202200359 35429012

[27] A. Tsouka , P. Dallabernardina , M. Mende , E. T. Sletten , S. Leichnitz , K. Bienert , K. Le Mai Hoang , P. H. Seeberger , F. F. Loeffler , J. Am. Chem. Soc. 2022, 144, 19832–19837. 36269942 10.1021/jacs.2c07285 PMC9634802

[28] M. Mende , A. Tsouka , J. Heidepriem , G. Paris , D. S. Mattes , S. Eickelmann , V. Bordoni , R. Wawrzinek , F. F. Fuchsberger , P. H. Seeberger , C. Rademacher , M. Delbianco , A. Mallagaray , F. F. Loeffler , Chem. A Eur. J. 2020, 26, 9954–9963. 10.1002/chem.202001291 PMC7496964 32315099

[29] A. Tsouka , M. Mende , J. Heidepriem , P. Dallabernardina , M. Garcia Ricardo , T. Schmidt , K. Bienert , P. H. Seeberger , F. F. Loeffler , Adv. Funct. Mater. 2024, 34, 2310980.

[30] K. Heiss , J. Heidepriem , N. Fischer , L. K. Weber , C. Dahlke , T. Jaenisch , F. F. Loeffler , J. Proteome Res. 2020, 19, 4339–4354. 32892628 10.1021/acs.jproteome.0c00484 PMC7640972

[31] S. Mashayekh , K. L. Bersch , J. Ramsey , T. Harmon , B. Prather , L. A. Genova , C. L. Grimes , J. Org. Chem. 2020, 85, 16243–16253. 33108204 10.1021/acs.joc.0c01852 PMC8115198

[32] S. Inamura , Y. Fujimoto , A. Kawasaki , Z. Shiokawa , E. Woelk , H. Heine , B. Lindner , N. Inohara , S. Kusumoto , K. Fukase , Org. Biomol. Chem. 2006, 4, 232–242. 16391765 10.1039/b511866b

[33] C. M. Vacariu , M. E. Tanner , Chem. A Eur. J. 2022, 28, e202200788. 10.1002/chem.202200788 35560956

[34] N. Wang , A. Hirata , K. Nokihara , K. Fukase , Y. Fujimoto , Pept. Sci. 2016, 106, 422–429. 10.1002/bip.22807 26773558

[35] P. Dallabernardina , V. Benazzi , J. D. Laman , P. H. Seeberger , F. F. Loeffler , Org. Biomol. Chem. 2021, 19, 9829–9832. 34734957 10.1039/d1ob01987b

[36] F. F. Loeffler , T. C. Foertsch , R. Popov , D. S. Mattes , M. Schlageter , M. Sedlmayr , B. Ridder , F. X. Dang , C. Von Bojničić-Kninski , L. K. Weber , A. Fischer , J. Greifenstein , V. Bykovskaya , I. Buliev , F. R. Bischoff , L. Hahn , M. A. R. Meier , S. Bräse , A. K. Powell , T. S. Balaban , F. Breitling , A. Nesterov-Mueller , Nat. Commun. 2016, 7, 1–9. 10.1038/ncomms11844 PMC4911634 27296868

[37] M. M. van der Kooi-Pol , C. P. de Vogel , G. N. Westerhout-Pluister , Y. K. Veenstra-Kyuchukova , J. C. Duipmans , C. Glasner , G. Buist , G. S. Elsinga , H. Westra , H. P. J. Bonarius , H. Groen , W. J. B. van Wamel , H. Grundmann , M. F. Jonkman , J. M. van Dijl , J. Invest. Dermatol. 2013, 133, 847–850. 23014336 10.1038/jid.2012.347

[38] F. Romero Pastrana , J. Neef , D. G. A. M. Koedijk , D. de Graaf , J. Duipmans , M. F. Jonkman , S. Engelmann , J. M. van Dijl , G. Buist , Sci. Rep. 2018, 8, 3234. 29459694 10.1038/s41598-018-21724-z PMC5818649

[39] A. Tsouka , K. Hoetzel , M. Mende , J. Heidepriem , G. Paris , S. Eickelmann , P. H. Seeberger , B. Lepenies , F. F. Loeffler , Front. Chem. 2021, 9, 931. 10.3389/fchem.2021.766932 PMC8589469 34778215

[40] S. E. Girardin , I. G. Boneca , J. Viala , M. Chamaillard , A. Labigne , G. Thomas , D. J. Philpott , P. J. Sansonetti , J. Biol. Chem. 2003, 278, 8869–8872. 12527755 10.1074/jbc.C200651200

[41] M. P. H. J. D. L. Ingrid A. Schrijver , Marjan van Meurs , Marie-José Melief , C. Wim Ang , D. Buljevac , R. Ravid , I. A. Schrijver , M. Van Meurs , M. J. Melief , C. W. Ang , D. Buljevac , R. Ravid , M. P. Hazenberg , J. D. Laman , Brain 2001, 124, 1544–1554. 11459746 10.1093/brain/124.8.1544

[42] C. Soliman , A. K. Walduck , E. Yuriev , J. S. Richards , C. Cywes-Bentley , G. B. Pier , P. A. Ramsland , J. Biol. Chem. 2018, 293, 5079–5089. 29449370 10.1074/jbc.RA117.001170 PMC5892565

[43] L. de Vor , B. van Dijk , K. van Kessel , J. S. Kavanaugh , C. de Haas , P. C. Aerts , M. C. Viveen , E. C. Boel , A. C. Fluit , J. M. Kwiecinski , G. C. Krijger , R. M. Ramakers , F. J. Beekman , E. Dadachova , M. G. E. H. Lam , H. C. Vogely , B. C. H. van der Wal , J. A. G. van Strijp , A. R. Horswill , H. Weinans , S. H. M. Rooijakkers , eLife 2022, 11, e67301. 34989676 10.7554/eLife.67301 PMC8751199

[44] H. C. Blenkinsopp , K. Seidler , M. Barrow , J. Am. Nutr. Assoc. 2023, 43, 59–76. 37294082 10.1080/27697061.2023.2211129

