# Supplementary material for: Synthetic High‐Throughput Microarrays of Peptidoglycan Fragments as a Novel Sero‐Diagnostic Tool for Patient Antibody Profiling
Source: Angew Chem Int Ed Engl. 2025 Feb 28;64(18):e202420874. doi: 10.1002/anie.202420874 (PMC12036811; doi:10.1002/anie.202420874)
Supplement: Supplementary file 1 — Supporting Information [file ANIE-64-e202420874-s001.pdf]

Angewandte Chemie
Eine Zeitschrift der Gesellschaft Deutscher Chemiker

Supporting Information

Synthetic High-Throughput Microarrays of Peptidoglycan Fragments as a Novel Sero-Diagnostic Tool for Patient Antibody Profiling

A. Tsouka, Y. Fu, M. G. Ricardo, P. H. Seeberger, Y. Wang, G. B. Pier, D. Schuppan, L. Boon, J. M. van Dijl, M. C. Bolling, G. Buist, F. F. Loeffler*, J. D. Laman**

Supporting Information

Synthetic high-throughput microarrays of peptidoglycan fragments as a novel sero-diagnostic tool for patient antibody profiling

Alexandra Tsouka,[a] Yanyan Fu,[b] Manuel G. Ricardo,[a] Peter H. Seeberger,[a,c] Yue Wang,[d] Gerald B. Pier,[e] Detlef Schuppan,[f,g] Louis Boon,[h] Jan Maarten van Dijl,[b] Maria C. Bolling,[i] Girbe Buist,[b]* Felix F. Loeffler,[a]* and Jon D. Laman[j]*

[a] Department of Biomolecular Systems, Max-Planck-Institute of Colloids and Interfaces, 14476 Potsdam, Germany

[b] University of Groningen, University Medical Center Groningen, Department of Medical Microbiology and Infection Prevention, Groningen, The Netherlands

[c] Institute of Chemistry and Biochemistry, Freie Universität Berlin, 14195, Berlin, Germany

[d] A*STAR Infectious Diseases Labs, Agency for Science, Technology and Research (A*STAR), Singapore 138648, Singapore

[e] Mass General Brigham, Harvard Medical School, Boston, MA 02115, USA

[f] Institute of Translational Immunology and Celiac Center, Medical Center, Johannes-Gutenberg University, 55099 Mainz, Germany

[g] Division of Gastroenterology, Beth Israel Deaconess Medical Center, Harvard Medical School, Boston, MA 02115, USA

[h] JJP Biologics, Warsaw, Poland

[i] University of Groningen, Department of Dermatology, UMCG Center of Expertise for Blistering Diseases, University Medical Center Groningen, The Netherlands

[j] University of Groningen, University Medical Center Groningen, Department of Pathology and Medical Biology, Groningen, The Netherlands

***Corresponding authors:**

Girbe Buist

E-mail: g.buist@umcg.nl

Felix F. Loeffler

E-mail: felix.loeffler@mpikg.mpg.de

Jon D. Laman

E-mail: j.d.laman@umcg.nl

Contents

A. General remarks ... 3

B. Preparation of solutions ... 4

C. Quantification of variations found in the peptide stems and the glycan core of PGNs ... 5

D. Proof of concept ... 6

D1. In-solution synthesis of MDP-like structure ... 6

D2. In-solution synthesis of pentapeptide disaccharide ... 7

E. Functionalization of acceptor glass slides for peptidoglycan synthesis ... 10

E1. *PEGMA/MMA β-Ala-X-β-Ala-NHR* ... 10

E2. *PEGMA/MMA β-Ala-X-β-Ala-Pra-NHAc* ... 11

F. Building block synthesis ... 11

G. Modules of synthesis ... 46

H. Synthesis & validation of PGN arrays by mAbs on different surface functionalizations ... 48

I. Optimization of LIFT conditions ... 53

J. Generation & screening of combinatorial PGN arrays via mAbs ... 55

K. Microarray with PGN oligosaccharide fragments ... 57

L. Epidermolysis bullosa (EB) patient sample screening ... 59

M. Fluorescence scan images of EB samples ... 65

P. References ... 73

Ethical Approval ... 74

Author contribution ... 74

A. General remarks

All applied solvents, deuterated solvents (99.5 atom% D), and chemicals were purchased from common suppliers such as Sigma-Aldrich, Alfa Aesar, Tokio Chemical Industry (TCI), Thermo Fischer Scientific Inc., Acros Organics B.V.B.A, Fluka BioChemika, Iris Biotech, and used without further purification. For High-Performance Liquid Chromatography (HPLC) solvents with corresponding quality were used. All carbohydrate starting materials used for the synthesis of the targeted compounds were purchased from GlycoUniverse GmbH & Co KGaA, 2-Azidoethyl 2-acetamido-2-deoxy-β-D-glucopyranoside (GlcNAc-N_3) from Synthose, while all amino acids from TCI, Novabiochem, Iris Biotech, Sigma-Aldrich and Bachem AG. Muramyl dipeptides (MDPs) **1** (L, D isomer, active) and **2** (L, L isomer, inactive) were obtained from InvivoGen. Monoclonal antibodies and secondary fluorescently labeled polyclonal antibodies were purchased from Bethyl Laboratories Inc, Biorad, Rockland Immunochemicals, Sigma-Aldrich, Thermo Fisher, if not stated otherwise. CF633 streptavidin was acquired from Biotium, Inc., and DyLight 633 *N*-hydroxysuccinimide ester (NHS), from Thermo Fisher Scientific Inc. Functionalized PEGMA/MMA-β-Ala-NHFmoc glass slides were acquired from PEPperPRINT GmbH, Heidelberg, Germany, while the SLEC PLT 7552 (styrene-butylacrylic copolymer) polymer matrix was acquired from Sekisui Chemical GmbH, Germany. If not mentioned otherwise, saturated aqueous solutions of inorganic salts were used. Thin layer chromatography (TLC) using silica gel coated aluminium plates (MACHEREY-NAGEL, pre-coated TLC sheets ALUGRAM® Xtra SIL G/UV254) was applied to monitor reactions until completion. Compounds were visualized by UV light (λ = 254 nm) or stained with Seebach (phosphomolybdic acid hydrate, cerium (IV) sulfate tetrahydrate, sulfuric acid and water. Flash column chromatography was carried out by using MACHEREY-NAGEL silica gel 60 (0.040 × 0.063 mm) and quartz sand. Deprotected polar products were lyophilized using a Christ Alpha 2–4 LD plus freeze dryer. The spectra were recorded on Varian 400-MR (400 MHz) or Bruker Ascend 400 (400 MHz) spectrometer. Chemical shifts δ are reported in ppm and are adjusted to internal standards of the residual proton signal of the deuterated solvent ($CDCl_3$: 7.26 ppm for ^1H and 77.0 ppm for ^{13}C, DMSO-*d6:* 2.50 ppm for ^1H and 39.5 ppm for ^{13}C, D_2O: 4.79 ppm for ^1H). The spectra were measured at room temperature (rt). IR spectra were recorded on a FT-IR spectrometer from Perkin-Elmer. High-resolution mass spectrometry (HR-MS) was conducted on a Waters Xevo G2-XS QTof device using ESI (electrospray ionization). Low-resolution mass spectrometry (LR-MS) were obtained using an HPLC-System Serie 1100 coupled with ESI-single quadrupole from Agilent. The abbreviation $[M+Na]^+$ refers to the product–sodium adduct. ESI mass spectra were run on IonSpec Ultima instruments and MALDI-ToF autoflexTM (Bruker) instrument. Analytical normal phase HPLC was performed on an HPLC-System Serie 1100 from Agilent using YMC-Diol-300 column (150 × 4.6 mm). Preparative normal phase HPLC was performed on an Agilent 1200 using a preparative YMC-Diol-300 column (150 × 20 mm). Analytical reverse phase HPLC was performed on an HPLC-System Series 1200 from Agilent using Hypercarb column (150 × 4.6 mm). Preparative reverse phase HPLC was performed on an Agilent 1200 using a preparative Hypercarb column (150 × 10 mm). UV-cleavage of the photo labile linker was performed in a Vilber Lourmat black light (VL.208.BL) lamp emitting 365 nm UV light with fractions of visible light (λ = 365 nm, filter size = 230 × 60 mm, power [W]: 2 x 8). A Molecular Devices microarray scanner, GenePix 4000B, San Jose, CA, was used for the analysis of all arrays. The detection wavelength was λ = 635 nm, with PMT gain 600, for all used secondary fluorescently labeled antibodies. The laser power was 33% for every measurement and the pixel size was 5 μm for high-resolution scans. The pH was determined using a FiveEasy pH/mV meter F20, equipped with a plastic pH electrode LE438 from Mettler Toledo.

Automated laser transfer system

The lasing system consists of a 405 nm wavelength diode laser with a Gaussian beam profile and a maximum of 300 mW power (iBeam smart 405-S, TOPTICA Photonics AG), led through a laser scanning system (intelliSCAN III 10, SCANLAB), linked to an f-theta- lens (JENar 170-355-140, JENOPTIK Optical Systems GmbH). The measured maximum power in the lasing area is 210 mW. Transport of donor slides between the slide holder and the lasing area is achieved with a KUKA AGILUS six KR 3 R540 robot (KUKA AG), with 20 µm precision. A robot tool, a gripper (with four 2 mm diameter rubber suction cups) is incorporated connected to a pneumatics system that initiates and releases vacuum for transportation. Within the lasing area, simple pressure is produced to ensure mechanical alignment, controlled by the pneumatics system. A strong vacuum (-80 kPa) suction is applied to keep the acceptor slide in place during the process.[1]

B. Preparation of solutions

- **Donor slide preparation:**

Pre-activated amino acids (3.00 mg) were dissolved in 50 µL anhydr. dimethylformamide (DMF), while SLEC (27.0 mg) was dissolved in 450 µL anhydr. dichloromethane (DCM). The first solution containing the activated amino acid was added into the second matrix solution resulting in 500 µL of spin coating solution. The final mixture was shaken for 2 min (vibrating orbital shaker) and afterwards the solution was spin-coated on top of the polyimide foil of the microscope glass slide, forming the thin layer of the transfer material.

Non-pre-activated amino acids (3.00 mg, 1.00 equiv.), *N,N'*-Diisopropylcarbodiimide (DIC) (1.00 equiv.), Pentafluorophenol (PfpOH) (1.00 equiv.) were dissolved in 50 µL anhydr. DMF, while SLEC (27.0 mg) were dissolved in 450 µL anhydr. DCM. The first solution containing the freshly activated amino acid was added into the second matrix solution. The final mixture was shaken for 2 min (vibrating orbital shaker) and afterwards the solution was spin-coated on top of the polyimide foil of the microscope glass slide, forming the thin layer of the transfer material. Non-pre-activated amino acid were prepared in-situ during the preparation of the spin-coating solution and were used without isolation or further characterization.

- **Fmoc-deprotection:** A solution of 20% piperidine in DMF (v/v) was prepared.
- **Capping:** A solution containing 10% Ac_2O and 20% *N,N*-diisopropylethylamine (DIPEA) in 70% DMF (v/v) was prepared.
- **Washing steps:** DMF (3 × 10 mL, 3 min), methanol (MeOH) (1 × 10 mL, 2 min), and dichloromethane (DCM) (1 × 10 mL, 1 min).
- **TFA-deprotection solution:** A solution containing 51% trifluoroacetic acid (TFA), 44% DCM, 3% triisobutylsilane (TIPS), and 2% water (v/v) was prepared.

C. Quantification of variations found in the peptide stems and the glycan core of PGNs

To estimate the number of potential natural and unnatural epitopes for antibody specificity screening, we quantified the variations that can exist in the stem peptides and the glycan core of peptidoglycans. This process involved considering the different modifications and variations observed in peptidoglycan structures across various bacterial species[1] omitting cross-linking variations of the interpeptide bridge, structures bearing only a single MurNAc glycan moiety, and glycan structures composed of more than two alternating monosaccharide units.

For this quantification, the core disaccharide unit of GlcNAc and MurNAc linked by a β-1,4 glycosidic bond was assumed to be constant. However, as reported in the literature, various modifications such as *N*-deacetylation and *O*-acetylation can occur in both sugar building blocks. Additional modifications on the MurNAc moiety, including phosphorylation, *N*-glycosylation, δ-lactam formation, and linkage with GlcNAc, were taken into consideration (**Figure S1**). In total, apart from the normal dimer structure, we considered that 10 different modifications can be observed in the GlcNAc and MurNAc moieties. This number was doubled to account for the dimer being in reverse order, leading to 22 different modifications

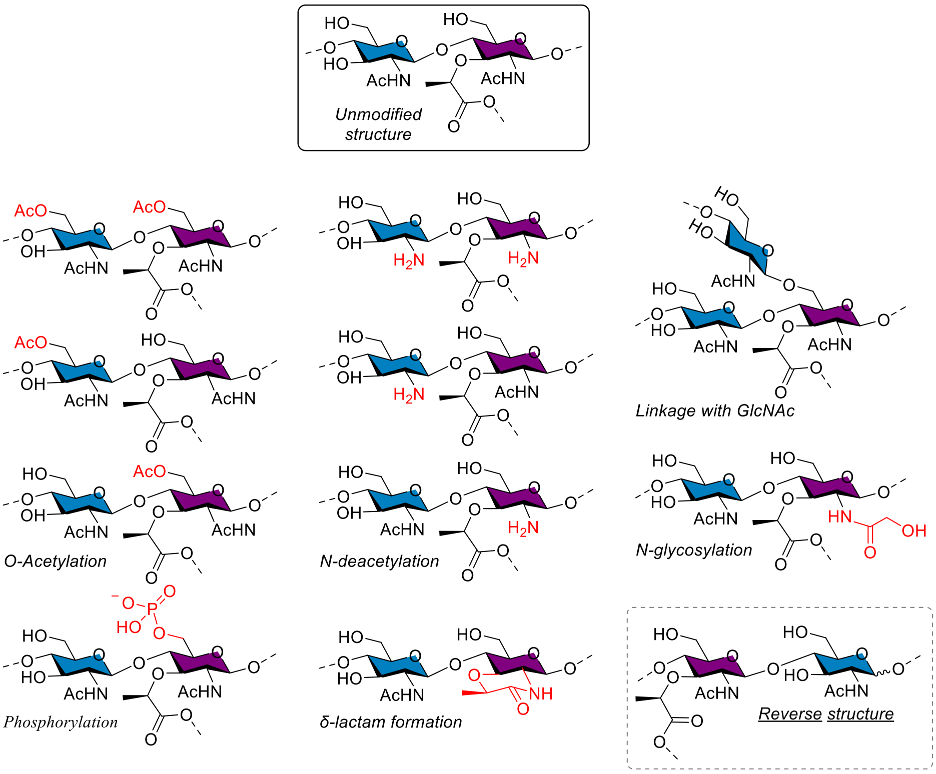

Figure S1. Modifications that can be detected on the sugar core.

Regarding the variations observed in the stem pentapeptides, we incorporated all variations indicated in the literature.[1] As reported by Vollmer *et al.*, there are 3 different amino acids that can be found in the 1st position, with L-alanine being the dominant one in most bacterial species. In the 2nd position, apart from D-isoGln and D-isoGlu found in Gram-positive and Gram-negative bacteria respectively, *threo*-3-hydroxyglutamate can also be found in *Microbacterium lacticum*. The 3rd position shows the greatest variation with up to 17 different amino acids. Lastly, the amino acids at positions 4 and 5 are typically both D-Ala, with the 5th position varying among strains to either D-lactate (D-Lac) or D-Ser. Thus, we calculated the number of stem peptide variants as follows:

Number of Stem Peptide Variants: **3** (AAs Pos.1) × **3** (AAs Pos.2) × **17** (AAs Pos.3) × **1** (AA Pos.4) × **3** (AAs Pos.5) = **459**. The number of interpeptide bridge variations has been excluded from this calculation.

Thus, the conservative number of variants that need to be screened for antibody specificity assessment can be generated from the following equation:

Total Variations = **459** *(stem pentapeptides)* × **22** *(sugar modifications)* = ***10.098*** different PGN fragments.

D. Proof of concept

D1. In-solution synthesis of MDP-like structure

Prior to laser synthesis of the desired PGN microarrays, the importance of the protecting groups on the carbohydrate moieties was investigated. For this purpose, the commercial photocleavable linker (**Scheme S1**) was covalently attached to the slide. A hydrophilic polyethylene glycol (PEG) linker, Fmoc-TTDS-OH, was then attached via amide coupling.[4] Subsequent removal of the Fmoc at the amine moiety allowed the synthesis of a MDP-like structure. L-Gln(Trt)-OPfp, L-Ala-OPfp, and deprotected MurNAc were sequentially used to synthesize the desired structure (**Scheme S1**). Between each synthesized layer, capping and Fmoc-deprotection on the N-terminus were performed, while coupling of each layer was achieved overnight at rt using the aforementioned sandwich method. Successful detection of the desired structure was achieved after cleavage under UV-light irradiation (365 nm), and MALDI-ToF mass spectrometry. The purity of the crude product was additionally analyzed using an analytical HPLC (Hypercarb column, 150 x 4.6 mm, 3 µm) flow rate of 0.7 mL/min with H_2O (0.1% formic acid) as eluents [isocratic (5 min), linear gradient to 20% ACN (30 min), linear gradient to 100% ACN (5 min)].

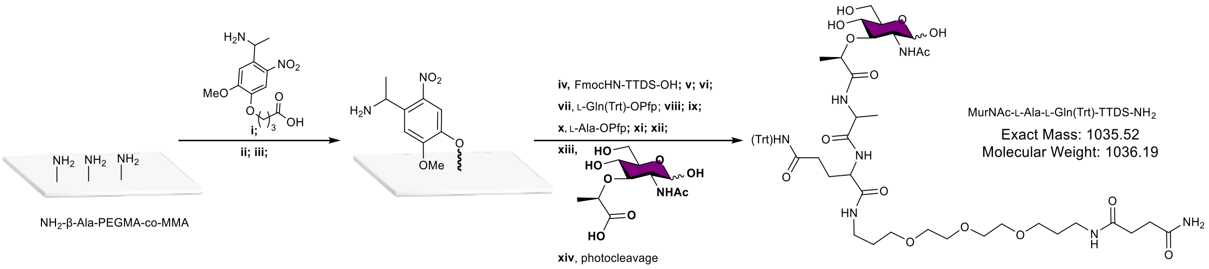

Scheme S1. Synthesis of MDP-like structure in solution. Reagents and conditions: i) Attachment of photo-linker with DIC, HOBt in anhydr. DMF at rt, overnight; ii) 10% Ac_2O, 20% DIPEA in DMF (v/v), rt, 30 min (×2); iii) 20% piperidine in DMF (v/v), iv) Attachment of PEG-spacer with DIC, HOBt in anhydr. DMF at rt, overnight; v) 10% Ac_2O, 20% DIPEA in DMF (v/v), rt, 30 min (×2); vi) 20% piperidine in DMF (v/v), vii) Attachment of L-Gln(Trt)-OPfp, with DIC, HOBt in anhydr. DMF at rt, overnight; viii) 10% Ac_2O, 20% DIPEA in DMF (v/v), rt, 30 min (×2); ix) 20% piperidine in DMF (v/v), x) Attachment of L-Ala-OPfp, with DIC, HOBt in anhydr. DMF at rt, overnight; xi) 10% Ac_2O, 20% DIPEA in DMF (v/v), rt, 30 min (×2); xii) 20% piperidine in DMF (v/v), xiii) Attachment of MurNAc, with DIC, HOBt in anhydr. DMF at rt, overnight; xiv) Photocleavage under UV-light (365 nm), 30 min.

MALDI-ToF

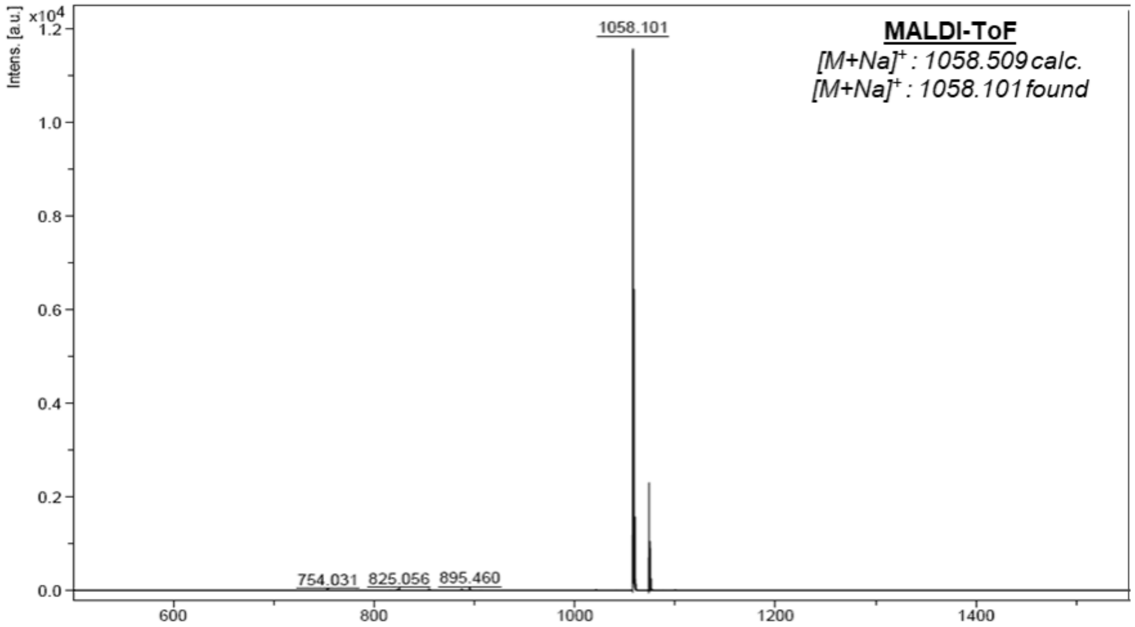

RP-HPLC (ELSD trace, method B, t_R = 45.1 min, t_R = 45.2 min of anomers)

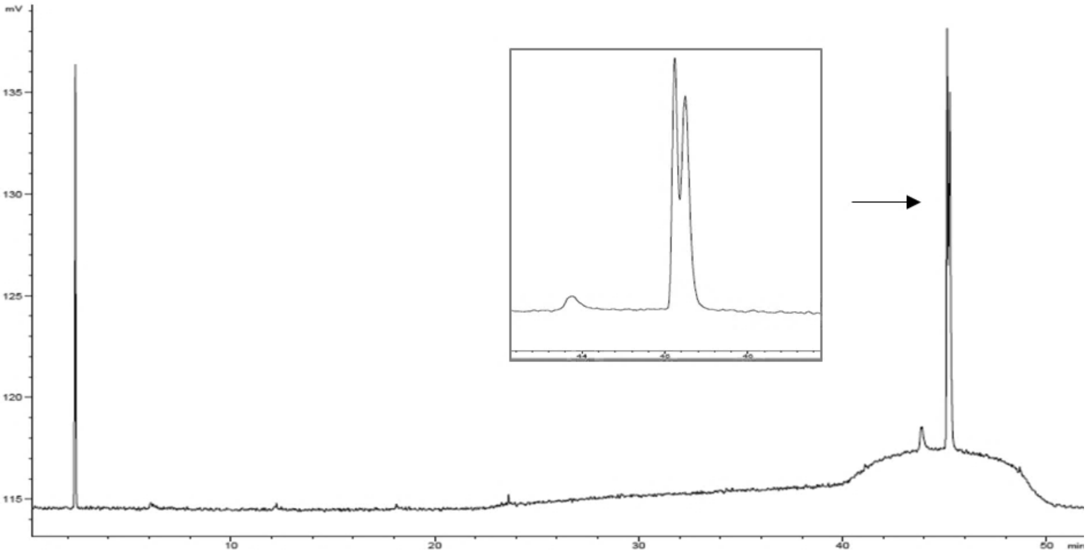

D2. In-solution synthesis of pentapeptide disaccharide

First, the commercial photocleavable linker (**Scheme S2**) was covalently attached to the slide and the Fmoc at the amine was removed for the synthesis of a pentapeptide stem. D-Ala-OH, L-Lys(Boc)-OPfp, D-iGln-OH, L-Ala-OPfp, and the deprotected dimer **4** were sequentially used to synthesize the desired structure. For the non-activated (-OH) building blocks, activation with PfpOH and DIC was achieved *in-situ* and used without purification. Between each building block layer, capping and Fmoc-deprotection of the N-terminus were performed, while coupling of each building block was achieved

overnight at rt using the previously introduced sandwich method. Subsequent treatment of the arrays with the TFA-deprotection solution, cleavage under UV-light irradiation (365 nm), and MALDI-ToF mass spectrometry revealed the successful synthesis of the desired pentapeptide disaccharide without any noticeable traces of a (hydrolyzed) pentapeptide monosaccharide. Very carefully following the protocols, this was the first time that we were able to detect a pentapeptide bearing a sugar (dimer) moiety *via* mass spectrometry after cleavage from a microarray slide. In addition, despite the expected very low yield of cleaved compound, we succeeded in identifying the crude product using analytical HPLC, Synergi Hydro RP18 column, 250 x 4.6 mm, flow rate of 1 mL/min with H_2O (0.1% formic acid) as eluents [isocratic (5 min), linear gradient to 20% MeCN (35 min), linear gradient to 100% MeCN (5 min)]. Although the signals were low, the results seem promising, showing two peaks for the two anticipated anomers of our pentapeptide bearing a sugar dimer.

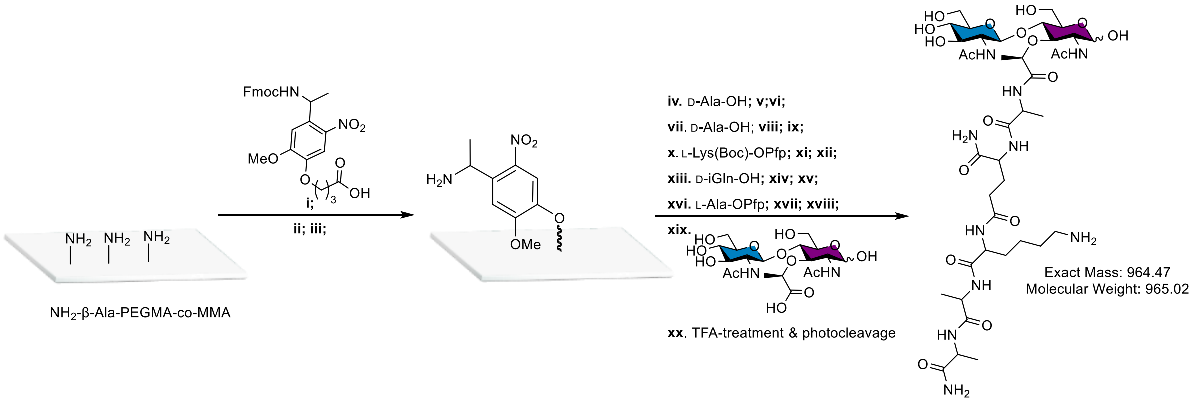

Scheme S2. Synthesis of MDP-like structure in-solution. Reagents and conditions: i) Attachment of photo-linker with DIC, HOBt in anhydr. DMF at rt, overnight; ii) 10% Ac_2O, 20% DIPEA in DMF (v/v), rt, 30 min (×2); iii) 20% piperidine in DMF (v/v), iv) Attachment of D-Ala-OH, with DIC, PfpOH in anhydr. DMF at rt, overnight; v) 10% Ac_2O, 20% DIPEA in DMF (v/v), rt, 30 min (×2); vi) 20% piperidine in DMF (v/v), vii) Attachment of D-Ala-OH, with DIC, PfpOH in anhydr. DMF at rt, overnight; viii) 10% Ac_2O, 20% DIPEA in DMF (v/v), rt, 30 min (×2); ix) 20% piperidine in DMF (v/v), x) Attachment of L-Lys(Boc)-OPfp in anhydr. DMF at rt, overnight; xi) 10% Ac_2O, 20% DIPEA in DMF (v/v), rt, 30 min (×2); xii) 20% piperidine in DMF (v/v), xiii) Attachment of D-iGln-OH with DIC, PfpOH in anhydr. DMF at rt, overnight; xiv) 10% Ac_2O, 20% DIPEA in DMF (v/v), rt, 30 min (×2); xv) 20% piperidine in DMF (v/v), xvi) Attachment of L-Ala-OPfp in anhydr. DMF at rt, overnight; xvii) 10% Ac_2O, 20% DIPEA in DMF (v/v), rt, 30 min (×2); xviii) 20% piperidine in DMF (v/v), xix) Attachment of dimer **4** with DIC, PfpOH in anhydr. DMF at rt, overnight; xx) TFA-treatment and photocleavage under UV-light (365 nm), 30 min.

MALDI-ToF

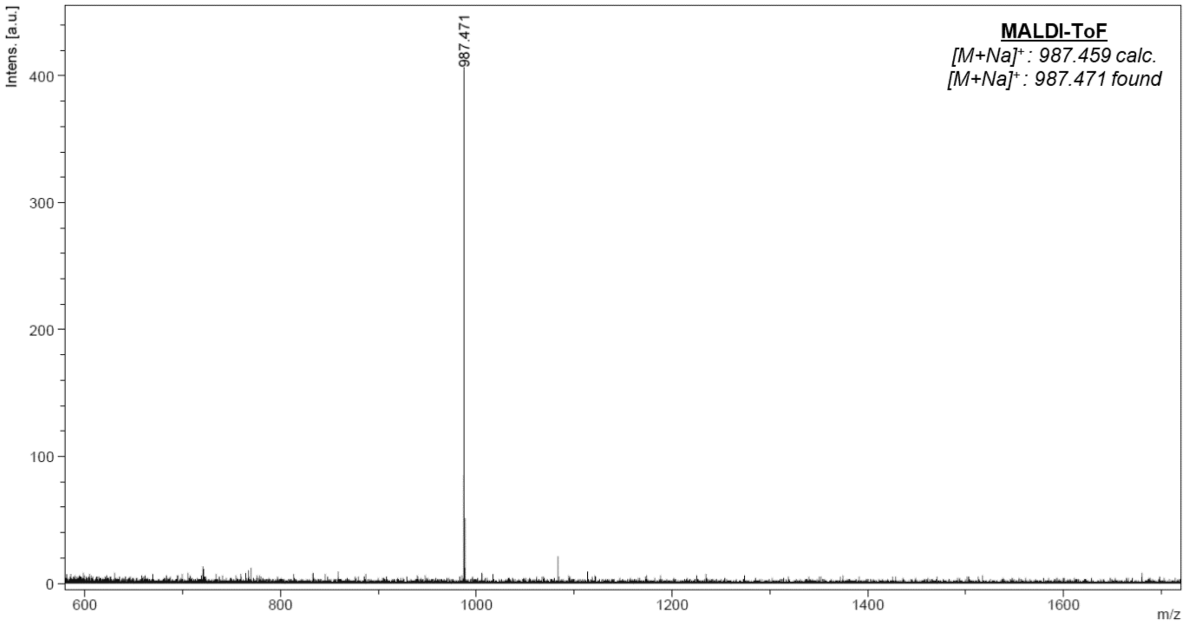

RP-HPLC (ELSD trace, t_R = 16.1 min, t_R = 17.4 min of anomers)

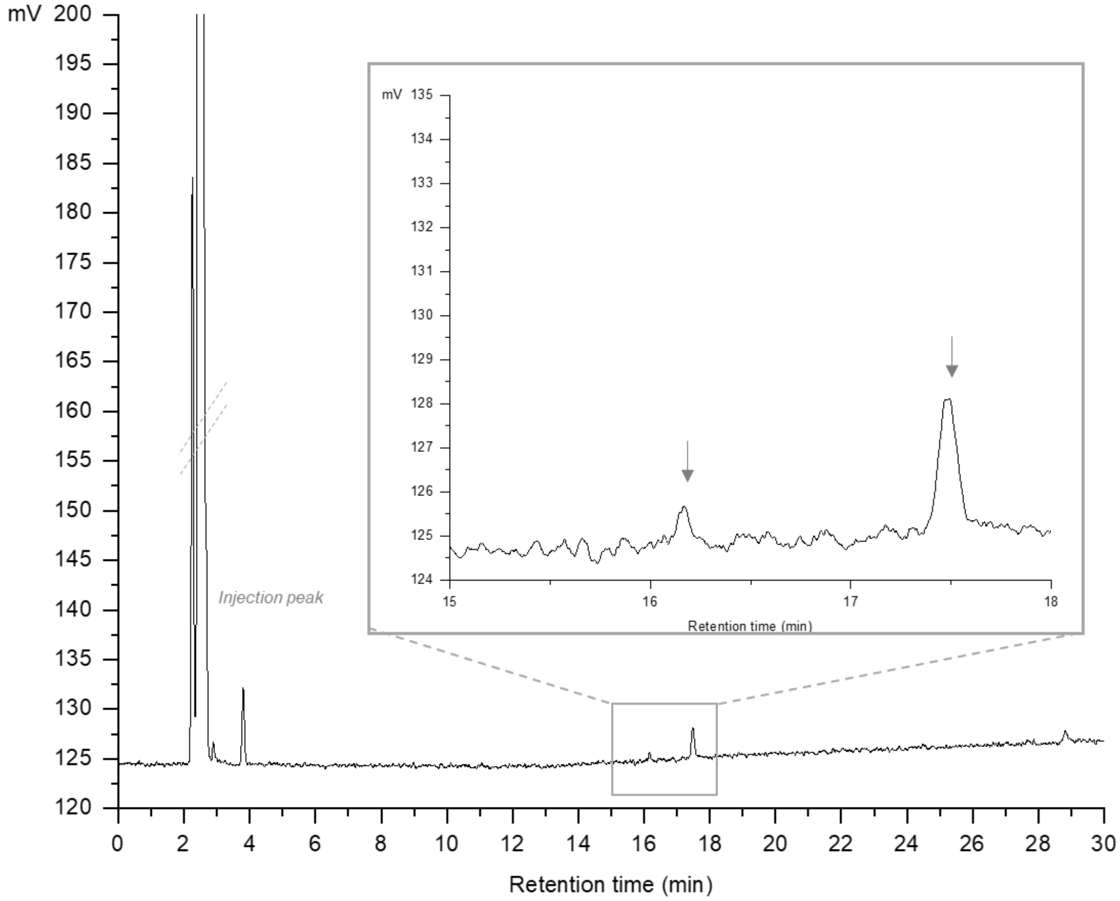

RP-HPLC (MSD trace, t_R = 16.1 min, t_R = 17.4 min of anomers)

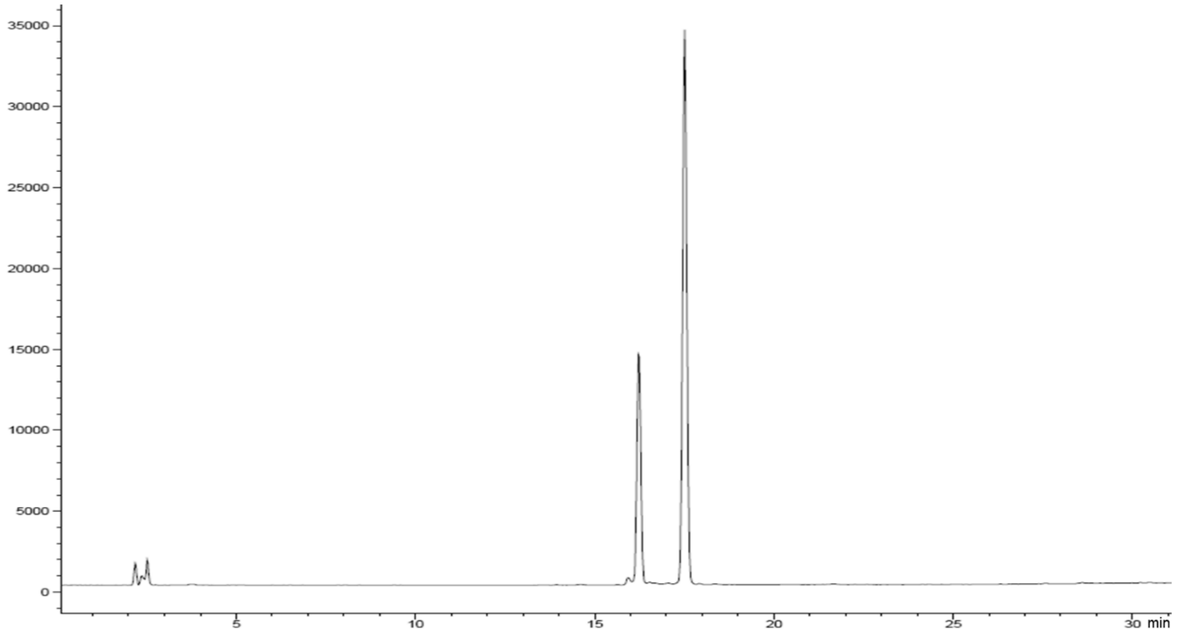

E. Functionalization of acceptor glass slides for peptidoglycan synthesis

E1. *PEGMA/MMA β-Ala-X-β-Ala-NHR*

The pre-functionalization was performed as reported in the literature.[2]

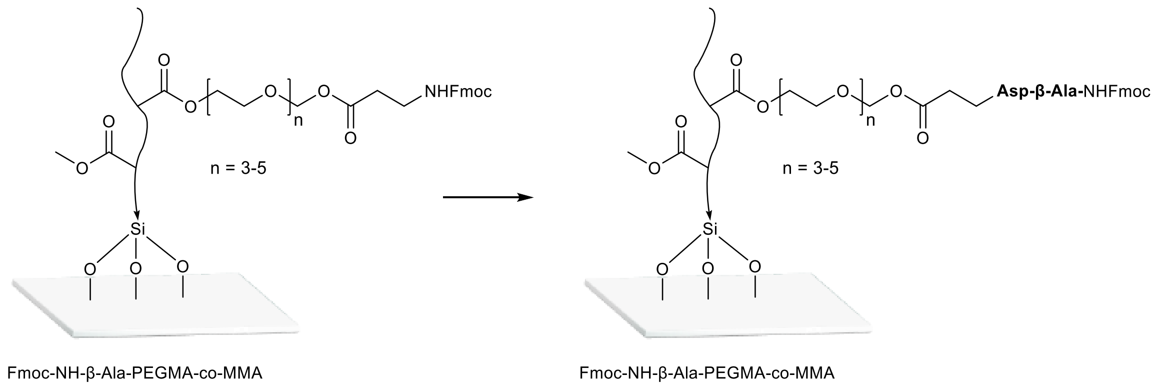

Fmoc-deprotection of commercially available Fmoc-protected acceptor slides (42 min):

The Fmoc-protected glass slides were pre-swelled in DMF for 10 min on a shaker in a petri dish. Afterwards, the slides were immersed in Fmoc-deprotection solution for 20 min on a shaker. The slides were washed consecutively with DMF (3×3 min), MeOH (1×2 min), DCM (1×1 min) and dried in a jet of air to obtain free amino glass slides.

Laser Pre-patterning with Fmoc–Asp(OtBu)–OPfp amino acid *(110 min):*

Fmoc–Asp(OtBu)–OPfp amino acid (see *Preparation of Solutions*, *Donor Slides*), was transferred via cLIFT to generate the desired pattern on the acceptor slide. The coupling reaction was accomplished under heat in an oven under nitrogen atmosphere at 95°C for 10 min. Subsequently the slides were washed with acetone twice. Initially for 2 min in an ultrasonic bath and then for another 2 min in a petri dish on a shaker (450 rps). Then, slides were

dried in a jet of air. The cLIFT transfer of the same amino acid pattern, the coupling, and the acetone wash steps were repeated twice. Each time a new donor slide was used for every transfer and coupling cycle. The remaining unreacted amino groups on the acceptor slides were subjected for acetylation for 2 min and then for 30 min. The same process was repeated with a freshly prepared capping solution for another 30 min at rt (300 rpm). The slides were washed with DMF (3×3 min), MeOH (1×2 min), DCM (1×1 min), and dried in a jet of air.

Attachment of Fmoc–β–Ala-OH (284 min):

Deprotection of the terminal Fmoc-groups was performed for 20 min with the Fmoc-deprotection solution. The slides were washed with DMF (3×3 min), MeOH (1×2 min), DCM (1×1 min), consecutively, and dried by a jet of air. Subsequently, Fmoc-β-Ala-OH (3.10 mg, 10 µmol, 1.00 equiv.), was dissolved in 250 µL of anhydrous DMF in a vial. DIC (1.56 µL, 10.0 µmol, 1.00 equiv.) and HOBt (1.40 mg, 10.0 µmol, 1.00 equiv.), were added consecutively and the vial was shaken for a few seconds. The resulting solution was pipetted on the amino glass slides and another slide was placed on top (sandwich functionalization method). The slides were to react for 4h in a petri dish. Then, the slides were washed consecutively with DMF (3×3 min), MeOH (1×2 min), and DCM (1×1 min), and dried by a jet of air. The remaining unreacted free NH_2 groups on the slides were subjected for acetylation using the capping solution (see *Preparation of Solutions*) for 2 min and then for 30 min. The same process was repeated with a freshly prepared capping solution for another 30 min at rt (300 rpm). Then, the slides were washed consecutively with DMF (3×3 min), MeOH (1 ×2 min), and DCM (1 × 1 min), and dried by a jet of air.

E2. *PEGMA/MMA β-Ala-X-β-Ala-Pra-NHAc*

For the functionalization of these slides, after the attachment of the second Fmoc–β–Ala-OH the slides were subjected to acetylation and Fmoc-deprotection as reported in previous sections using the respective solutions (see *Preparation of Solutions*).

Laser patterning with Fmoc–Pra–OH amino acid *(110 min):*

The Fmoc-L-propargylglycine, (Fmoc-propargyl-Gly-OH) amino acid building block (pre-activated with Pfp-OH, as described above) was transferred *via* cLIFT to generate the desired pattern on the acceptor slide as reported in the literature. Then, deprotection of the terminal Fmoc-groups and acetylation of the free NH_2 groups was performed as previously reported.[4]

F. Building block synthesis

Synthesis of 2-acetamido-2-deoxy-β-D-glucopyranosyl-(1→4)-2-acetamido-3-*O*-[(R)-1-carboxyethoxy]-2-deoxy-β-D-glucopyranoside 4

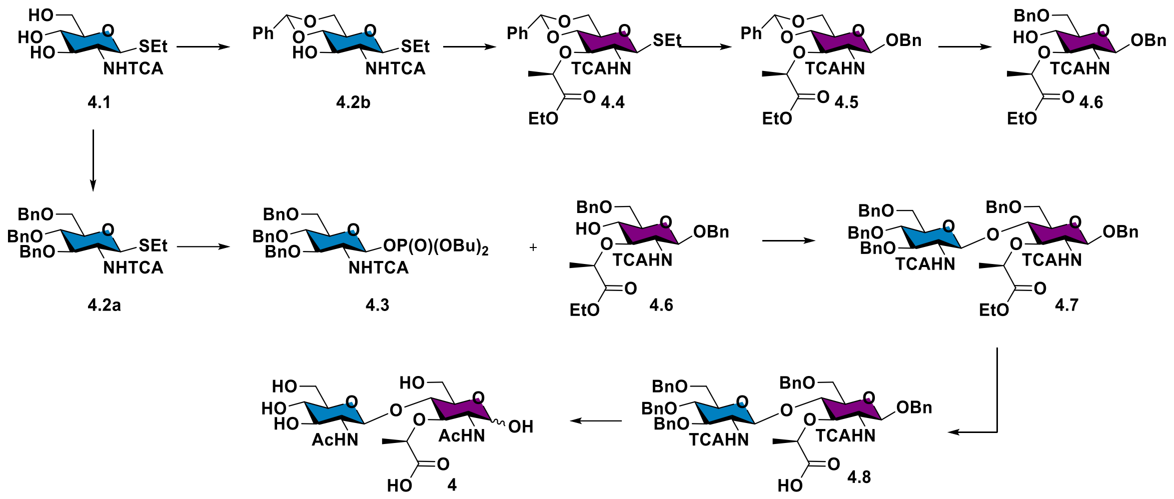

*Synthesis of Ethyl 2-deoxy-3,4,6-tri-O-benzyl-1-thio-2-trichloroacetamido-β-D-glucopyranoside **4.2a***

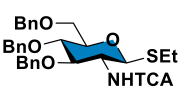

4.2a

To a solution of **4.1** (2.00 g, 5.40 mmol) in DMF (30 mL), benzyl bromide (BnBr) (3.22 mL, 27.2 mmol) was added. The mixture was cooled down at -10 °C and NaH (60% in mineral oil, 0.87 g, 21.7 mmol) was added. After 30 min, the reaction was quenched with 10% citric acid solution (20 mL) and diluted with ethyl acetate (80 mL). The organic layer was separated and washed with water (2×20 mL) and NaCl solution (2×20 mL). The solution was dried over Na_2SO_4 and the solvent was removed under reduced pressure. The crude product was purified by flash column chromatography using a mixture of hexane/dichloromethane/ethyl acetate (15:5:1) as eluent. The product was obtained as a white solid in 81% yield (2.80 g, 4.40 mmol). R_f (hex/DCM/EA 6:3:1): 0.6. ^{1}H NMR (400 MHz, $CDCl_3$): δ 7.42 – 7.29 (m, 15H), 7.21 (dd, *J* = 7.3, 2.4 Hz, 2H), 6.87 (d, *J* = 8.3 Hz, 1H), 4.91 – 4.72 (m, 4H), 4.67 – 4.55 (m, 3H), 4.03 (dd, *J* = 9.7, 8.5 Hz, 1H), 3.83 – 3.70 (m, 4H), 3.61 (ddd, *J* = 9.5, 4.2, 2.4 Hz, 1H), 2.85 – 2.66 (m, 2H), 1.31 (t, *J* = 7.4 Hz, 3H) ppm. ^{13}C NMR (101 MHz, $CDCl_3$): δ 161.5, 138.0, 137.8, 137.7, 128.5, 128.4, 128.4, 127.9, 127.9, 127.8, 127.7, 127.6, 82.6, 81.8, 79.3, 78.4, 75.2, 74.8, 73.5, 68.9, 57.3, 24.4, 15.1 ppm. ESI-HRMS: m/z [M-H]$^-$ calcd. for $C_{31}H_{33}Cl_3NO_5S$ 635.5702 found 635.5691.

^{1}H NMR ($CDCl_3$)

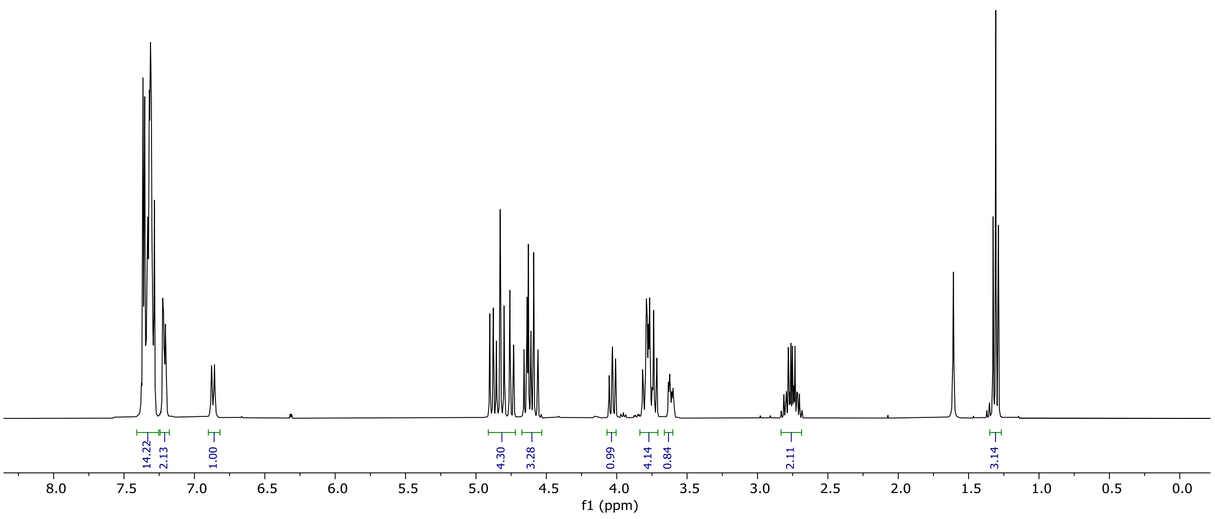

^{13}C APT NMR ($CDCl_3$)

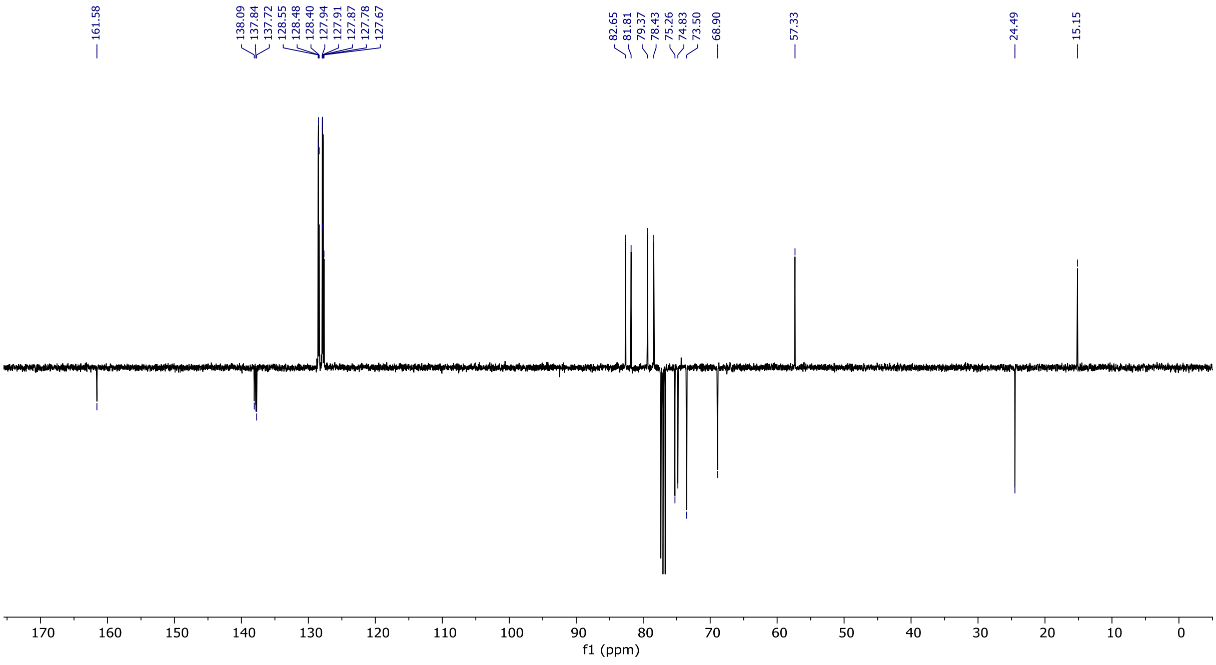

HSQC NMR ($CDCl_3$)

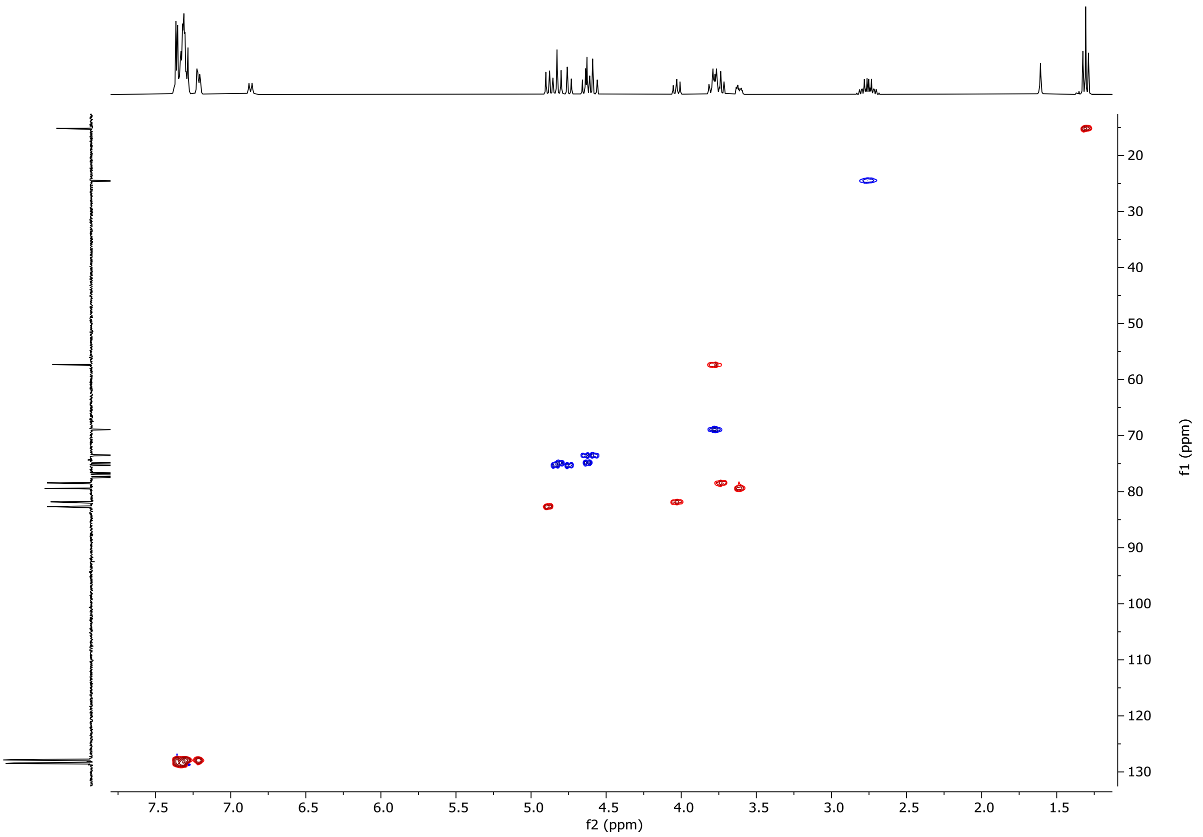

Synthesis of Dibutylphosphoryloxy 2-deoxy-3,4,6-tri-O-benzyl-2-trichloroacetamido-β-D-glucopyranoside ***4.3***

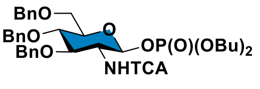

4.3

4.2a (1.00 g, 1.57 mmol) was dissolved in anhydrous DCM (20 mL), under argon atmosphere. Dibutylphosphate (0.62 mL, 3.34 mmol) and 4Å molecular sieves were added, and the suspension was stirred for 1 h. The mixture was cooled down to -15 °C, NIS (0.59 g, 2.35 mmol) and TfOH (14 µL, 0.16 mmol) were added dropwise and stirring was continued for further 60 min at -15 °C. After that the mixture was allowed to warm over 1 h to 0 °C and stirred for an additional 60 min. The reaction was then quenched with $NaHCO_3$ solution (10 mL), diluted with chloroform, and allowed to reach rt. The molecular sieves were filtered off and the mixture was washed with 10% $Na_2S_2O_3$ solution (20 mL) and NaCl solution (20 mL). The solution was dried over Na_2SO_4 and the solvent was removed under reduced pressure. The crude product was purified by flash column chromatography using a mixture of hexane/dichloromethane/ethyl acetate (6:3:1) as eluent. The product was obtained as a colorless foam in 90% yield (1.10 g, 1.40 mmol). R_f (hex/DCM/EA 4:2:1): 0.6. ^{1}H NMR (400 MHz, $CDCl_3$): δ 7.38 – 7.27 (m, 15H), 7.20 (dd, *J* = 7.3, 2.3 Hz, 2H), 6.86 (d, *J* = 8.9 Hz, 1H), 5.75 (dd, *J* = 5.8, 3.3 Hz, 1H), 4.85 – 4.74 (m, 3H), 4.66 – 4.50 (m, 3H), 4.37 – 4.28 (m, 1H), 4.14 – 4.01 (m, 5H), 3.92 – 3.86 (m, 2H), 3.81 (dd, *J* = 10.9, 3.6 Hz, 1H), 3.69 (dd, *J* = 10.9, 2.0 Hz, 1H), 1.70 – 1.58 (m, 4H), 1.46 – 1.31 (m, 4H), 0.97 – 0.89 (m, 6H) ppm. ^{13}C NMR (101 MHz, $CDCl_3$): δ 161.9, 137.7, 137.6, 137.5, 128.6, 128.5, 128.4, 128.4, 127.9, 127.9, 127.8, 127.8, 96.1, 96.0, 78.9, 77.6, 75.3, 75.0, 73.5, 73.5, 73.0, 68.2, 68.1, 68.1, 68.1, 67.9, 54.8, 54.7, 32.2, 32.2, 32.1, 32.1, 18.6, 13.6, 13.5 ppm. ESI-HRMS: m/z $[M-H]^+$ calcd. for $C_{37}H_{47}{}^{35}Cl_3NNaO_9P$ 802.2026 found 808.1952.

^{1}H NMR ($CDCl_3$)

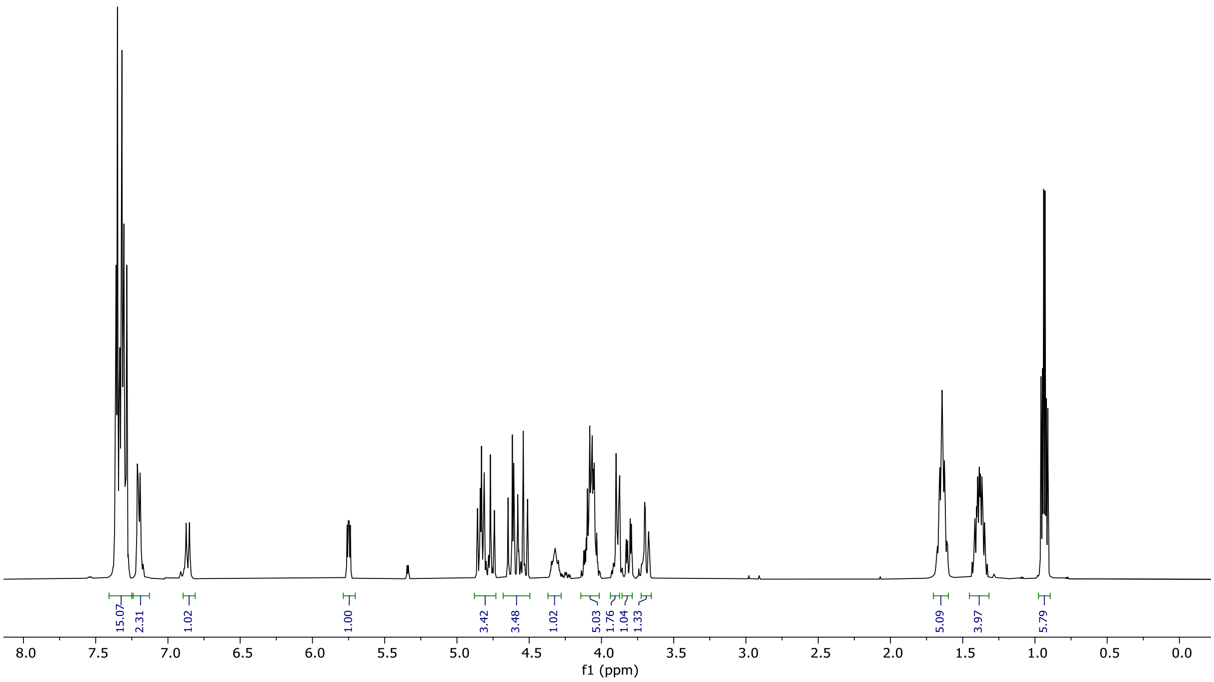

^{13}C APT NMR (CDCl3)

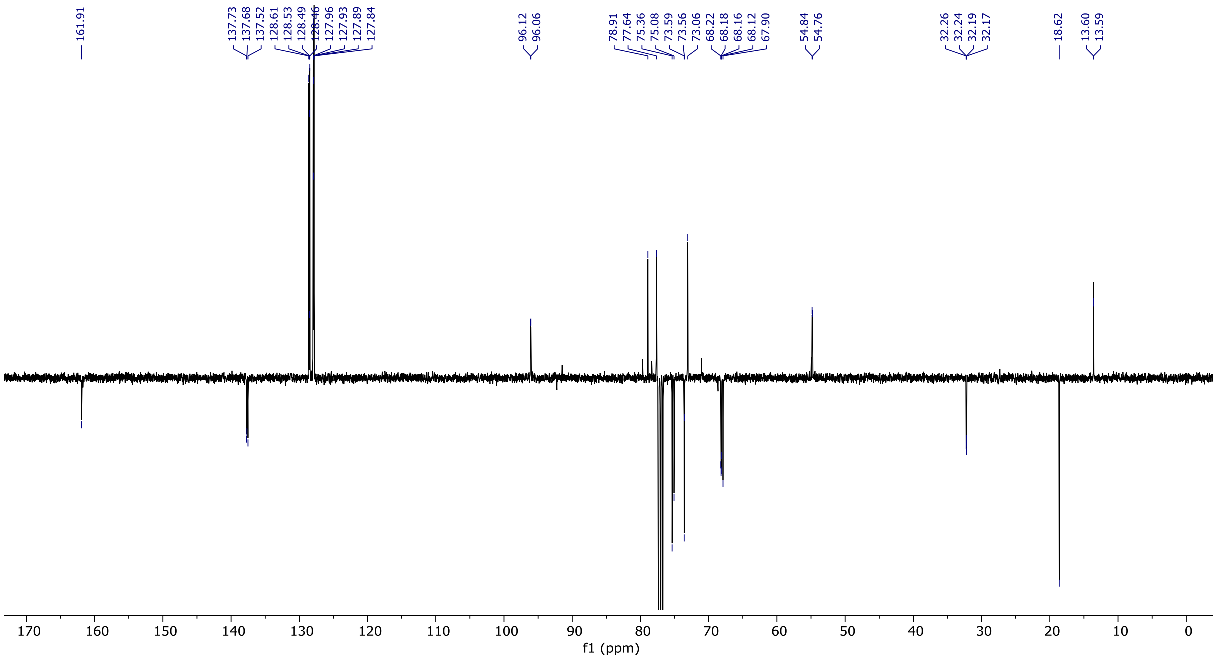

HSQC NMR ($CDCl_3$)

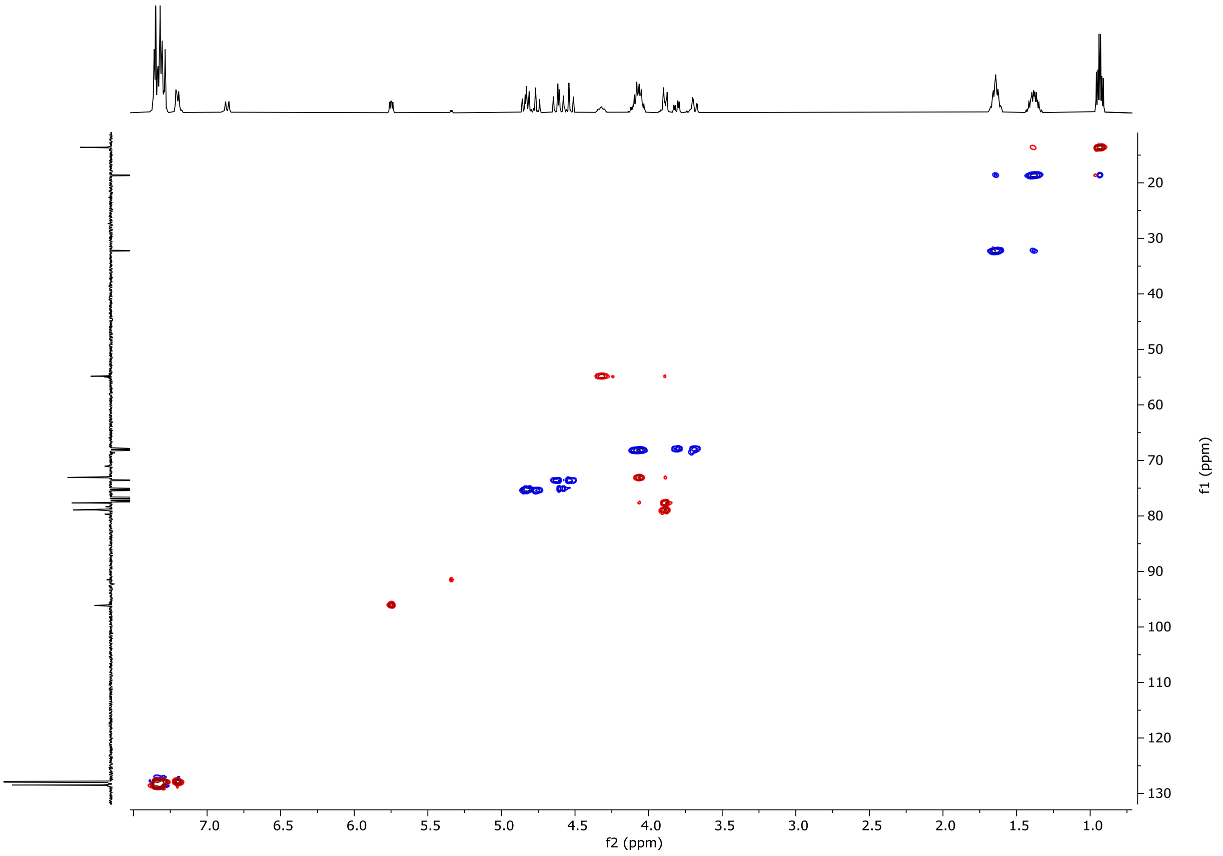

Ethyl 2-deoxy-4,6-O-benzylidene-1-thio-2-trichloroacetamido-β-D-glucopyranoside ***4.2b*** *and ethyl 2-deoxy-3-O-((R)-1'-ethoxycarbonylethyl)-4,6-O-benzylidene-1-thio-2-trichloro-acetamido-β-D-glucopyranoside* ***4.4*** were synthesized as reported in the literature.[3,4]

Synthesis of Benzyl 2-deoxy-3-O-((R)-1'-ethoxycarbonylethyl)-4,6-O-benzylidene-2-trichloro-acetamido-β-D-glucopyranoside ***4.5***

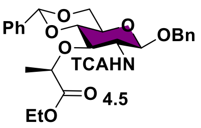

4.4 (0.72 g, 1.30 mmol) was dissolved in anhydr. DCM (20 mL) under argon atmosphere. Benzylic alcohol (0.27 mL, 2.60 mmol) and 4Å molecular sieves were added, and the suspension was stirred for 1 h at rt. The mixture was cooled down to -15 °C, NIS (0.50 g, 1.90 mmol) and TfOH (11.0 µL, 0.13 mmol) were added dropwise and stirring was continued for further 60 min at -15 °C. After that the mixture was allowed to warm up to 10 °C and stirred for additional 6 h. The reaction was then quenched with NaHCO3 solution (10 mL), diluted with chloroform, and allowed to reach rt. The molecular sieves were filtered off and the mixture was washed with 10% $Na_2S_2O_3$ solution (20 mL) and NaCl solution (20 mL). The solution was dried over Na_2SO_4 and the solvent was removed under reduced pressure. The crude product was purified by flash column chromatography using a mixture of hexane/dichloromethane/ethyl acetate (10:5:1) as eluent. The product was obtained as a white solid in 78% yield (0.61 g, 1.00 mmol). R_f (Hex/DCM/EA 6:3:1): 0.5. α_D^{25} = -47.8 (c 1.0, $CHCl_3$). ^1H NMR (400 MHz, $CDCl_3$): δ 7.91 (d, *J* = 6.0 Hz, 1H), 7.51 – 7.31 (m, 10H), 5.61 (s, 1H), 4.95 (d, *J* = 12.3 Hz, 1H), 4.70 – 4.61 (m, 2H), 4.56 (q, *J* = 6.9 Hz, 1H), 4.41 (dd, *J* = 10.5, 5.0 Hz, 1H), 4.25 – 4.17 (m, 1H), 4.17 – 4.07 (m, 1H), 4.01 – 3.92 (m, 2H), 3.88 (t, *J* = 10.3 Hz, 1H), 3.79 – 3.72 (m, 1H), 3.45 (td, *J* = 9.7, 5.0 Hz, 1H), 1.40 (d, *J* = 6.9 Hz, 3H), 1.26 (t, *J* = 7.1 Hz, 3H) ppm. ^{13}C NMR (101 MHz,

CDCl3): δ 129.1, 128.4, 128.3, 127.8, 127.7, 125.9, 101.2, 101.0, 82.5, 75.6, 74.7, 70.8, 68.6, 66.4, 61.2, 56.8, 18.7, 14.1 ppm. ESI-HRMS: m/z [M-H]$^-$ calcd. for $C_{27}H_{29}Cl_3NO_8$ 600.0959 found 600.0994

^{1}H NMR ($CDCl_3$)

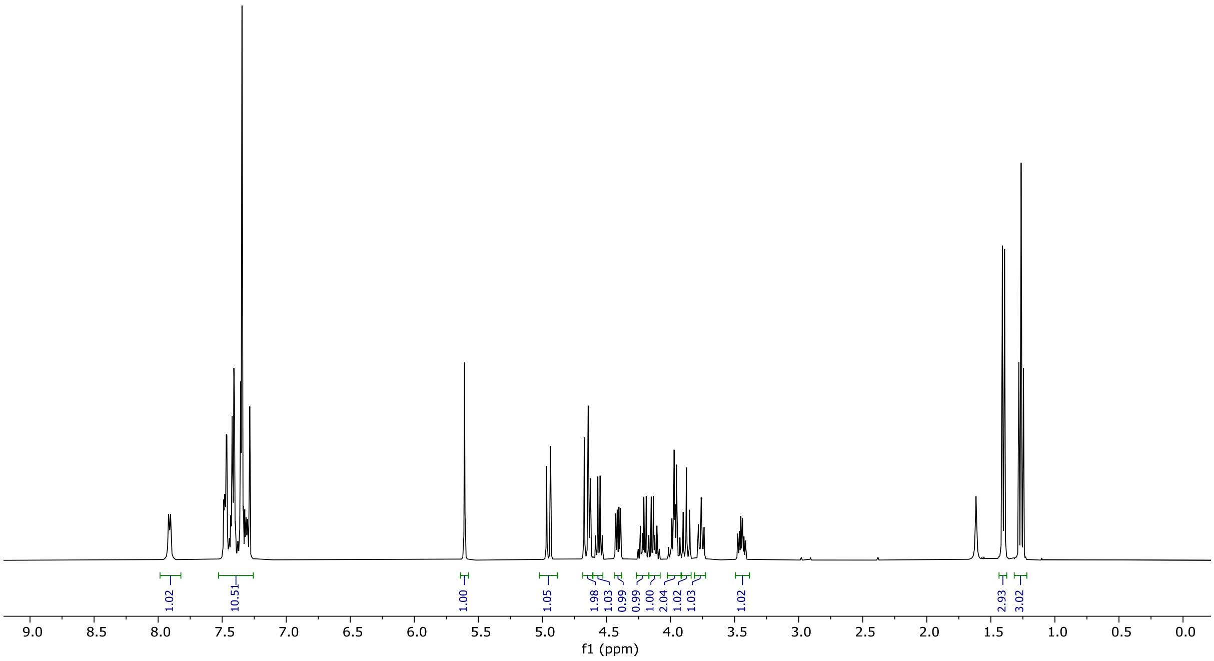

^{13}C APT NMR ($CDCl_3$)

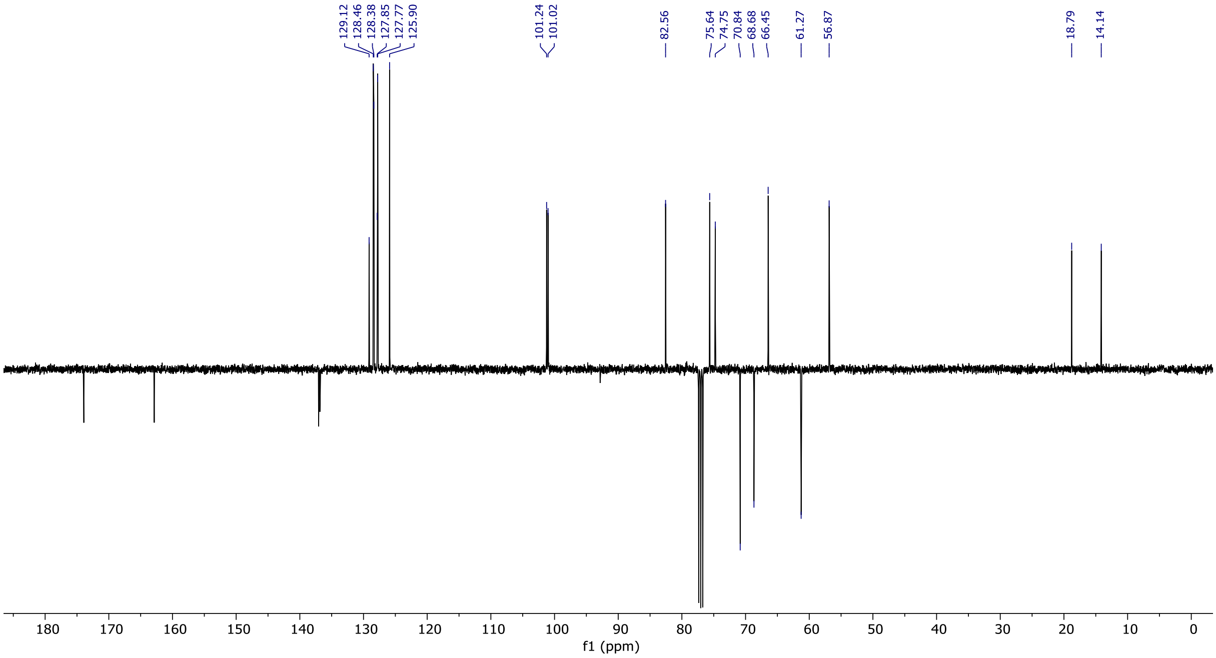

HSQC NMR ($CDCl_3$)

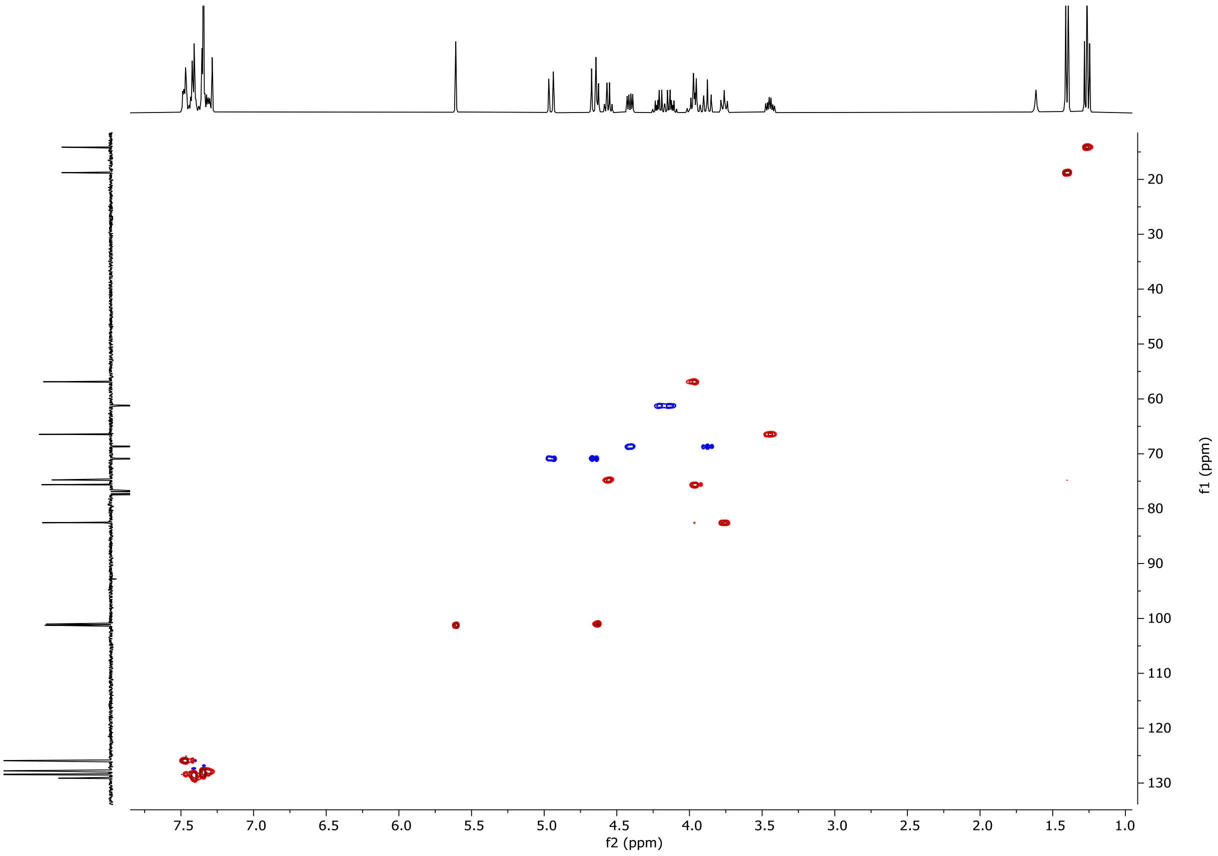

*Synthesis of Benzyl 2-deoxy-3-O-((R)-1'-ethoxycarbonylethyl)-6-O-benzy-2-trichloro-acetamido-β-D-glucopyranoside (**4.6**)*

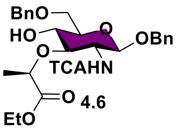

To a solution of **4.5** (0.60 g, 1.00 mmol) in anhydr. acetonitrile (50 mL), $Me_3N•BH_3$ (0.22 mg, 3.00 mmol) and $BF_3•OEt_2$ (0.37 mL, 3.00 mmol) were sequentially added under argon atmosphere at 0 °C. The mixture was stirred for 1 h and then allowed to reach rt. Then, it was diluted with ethyl acetate (80 mL) and quenched with $NaHCO_3$ solution (10 mL). The organic layer was separated and washed with 10% citric acid (20 mL), $NaHCO_3$ solution (20 mL), and NaCl solution (20 mL). The solution was dried over Na_2SO_4 and the solvent was removed under reduced pressure. The crude product was purified by flash column chromatography using a mixture of toluene/acetonitrile (10:1) as eluent. The product was obtained as a white solid in 55% yield (0.33 g, 0.55 mmol). R_f (toluene/MeCN 20:1): 0.25. α_D^{25} = -35.8 (*c* 1.0, $CHCl_3$). ^1H NMR (400 MHz, $CDCl_3$): δ 7.89 (d, *J* = 7.2 Hz, 1H), 7.44 – 7.26 (m, 10H), 4.91 (d, *J* = 12.4 Hz, 1H), 4.71 (q, *J* = 7.0 Hz, 1H), 4.67 – 4.62 (m, 2H), 4.63 – 4.56 (m, 1H), 4.50 (d, *J* = 8.1 Hz, 1H), 4.27 – 4.10 (m, 2H), 3.95 – 3.84 (m, 2H), 3.81 – 3.74 (m, 2H), 3.69 (dd, *J* = 10.8, 8.7 Hz, 1H), 3.47 (ddd, *J* = 9.1, 6.5, 4.6 Hz, 1H), 1.40 (d, *J* = 6.9 Hz, 3H), 1.28 (t, *J* = 7.1 Hz, 3H) ppm. ^{13}C NMR (101 MHz, $CDCl_3$): δ 174.6, 162.7, 137.2, 137.1, 128.6, 128.3, 128.1, 127.8, 127.7, 127.6, 100.5, 78.4, 74.9, 73.9, 73.8, 72.9, 71.3, 70.4, 61.3, 55.6, 18.9, 14.1 ppm. ESI-HRMS: m/z $[M-Na]^+$ calcd. for $C_{27}H_{32}Cl_3NNaO_8$ 626.1091 found 626.1136.

^{1}H NMR ($CDCl_3$)

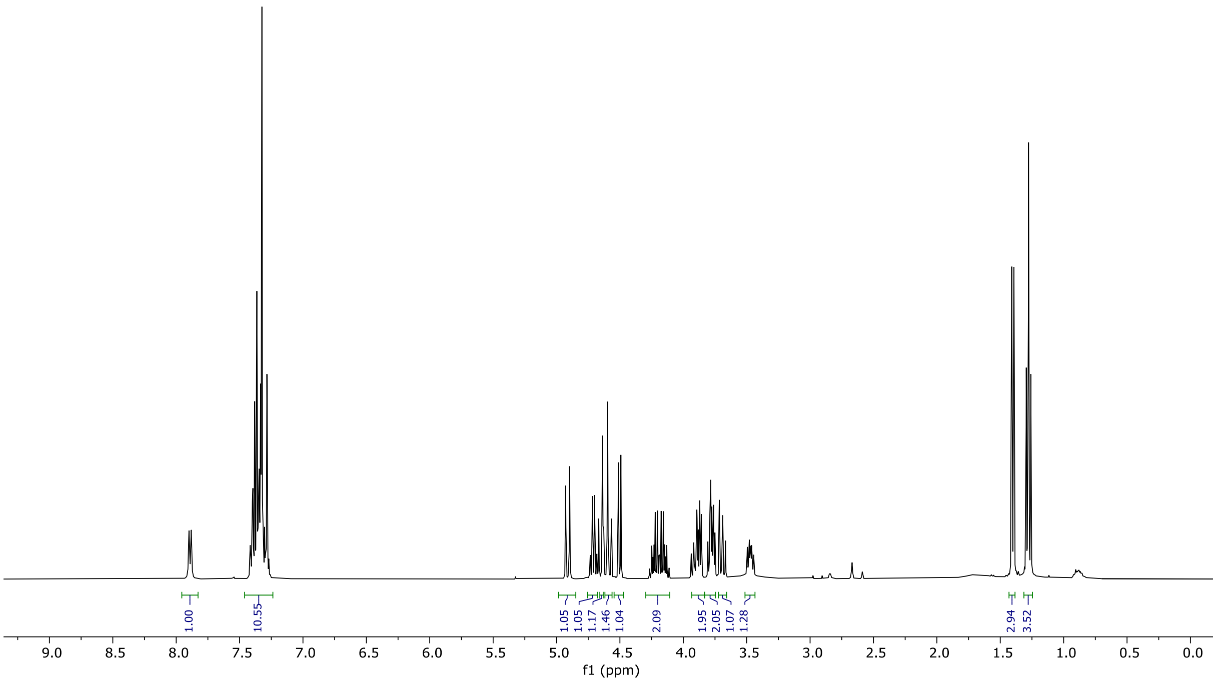

^{13}C APT NMR ($CDCl_3$)

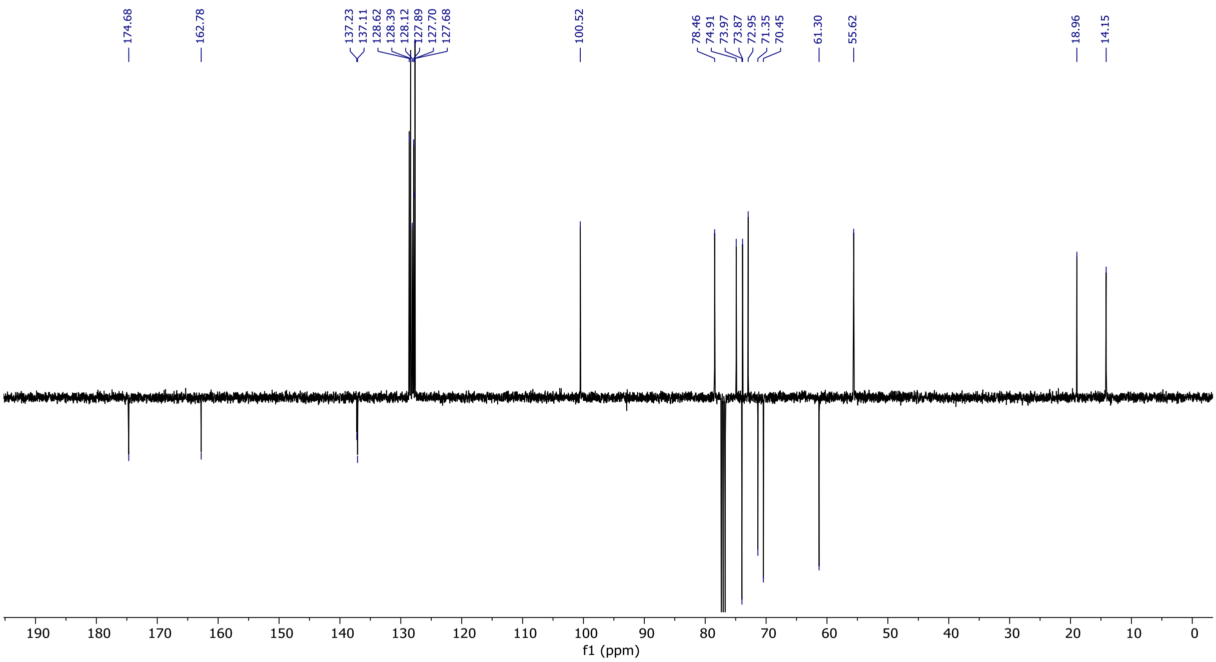

HSQC NMR ($CDCl_3$)

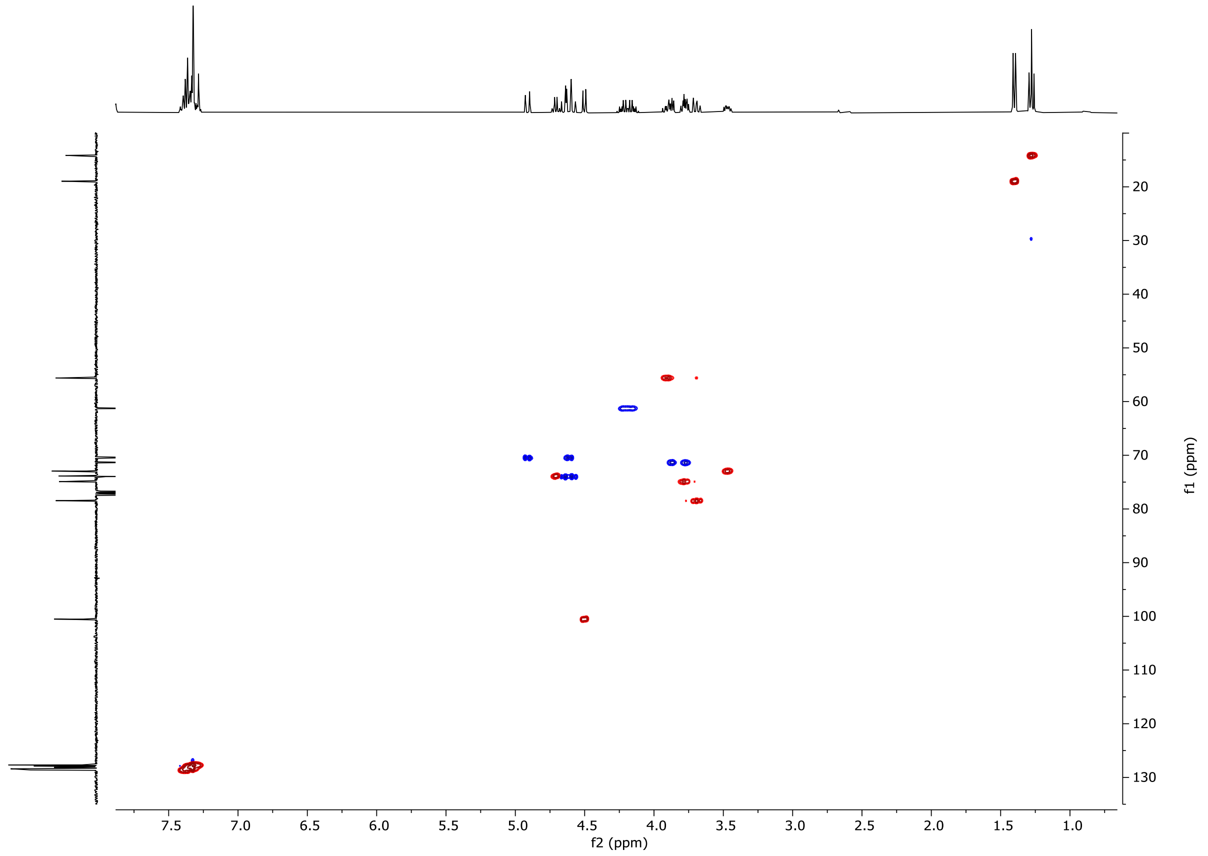

Synthesis of Benzyl 2-deoxy-3,4,6-tri-O-benzyl-2- trichloro-acetamido-β-D-glucopyranosyl-(1→4)-2-deoxy-3-O-[(R)-1`-ethoxycarbonylethyl]-6-O-benzyl-2-trichloro-acetamido-β-D-glucopyranoside ***4.7***

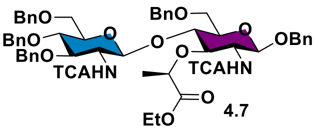

4.3 (0.48 g, 0.62 mmol) and **4.6** (0.25 g, 0.41 mmol) were dissolved in anhydr. DCM (10 mL), 4Å molecular sieves were added under argon atmosphere, and the suspension was stirred for 60 min at rt. The mixture was cooled down to -30 °C and TMSOTf (37.0 µL, 0.21 mmol) dissolved in anhydr. DCM (0.50 mL) was added dropwise. After 60 min, the reaction left to reach -10 °C, and additional TMSOTf (37.0 µL, 0.21 mmol) was added. Until completion, the reaction mixture was quenched with $NaHCO_3$ solution (5 mL), diluted with chloroform, left to reach rt. The molecular sieves were filtered off. The organic layer was washed with NaCl solution (2×10 mL), and dried over Na_2SO_4 and the solvent was removed under reduced pressure. The crude product was purified by flash column chromatography using a mixture of hexane/dichloromethane/ethyl acetate (6:3:1) as eluent. The product was obtained as a white solid in 66% yield (0.29 g, 0.24 mmol). R_f (hex/EA 4:2:1): 0.5. ^{1}H NMR (400 MHz, $CDCl_3$): δ 8.36 (d, J = 6.9 Hz, 1H), 7.32 – 7.08 (m, 25H), 6.37 (d, J = 8.5 Hz, 1H), 4.84 (d, J = 12.6 Hz, 1H), 4.73 – 4.67 (m, 3H), 4.62 (q, J = 7.0 Hz, 1H), 4.55 – 4.47 (m, 5H), 4.41 (d, J = 8.1 Hz, 1H), 4.38 (d, J = 8.3 Hz, 1H), 4.29 (d, J = 8.0 Hz, 1H), 4.18 – 4.06 (m, 1H), 4.06 – 3.95 (m, 2H), 3.96 – 3.85 (m, 1H), 3.75 – 3.65 (m, 4H), 3.64 – 3.46 (m, 5H), 3.24 (tt, J = 9.2, 2.9 Hz, 2H), 1.21 (d, J = 6.9 Hz, 3H), 1.17 (t, J = 7.2 Hz, 3H) ppm. ^{13}C NMR (101 MHz, $CDCl_3$): δ 175.2, 163.0, 161.5, 138.0, 137.7, 137.7, 137.7, 137.6, 137.3, 128.8, 128.7, 128.6, 128.5, 128.5, 128.4, 128.3, 128.3, 128.2, 127.9, 127.9, 127.8, 127.8, 127.7, 127.6, 127.5, 127.4, 101.1, 99.0, 93.1, 92.5, 80.9, 78.2, 76.9,

76.2, 75.1, 74.7, 74.7, 74.6, 74.5, 73.6, 73.4, 73.2, 70.2, 68.4, 68.1, 61.2, 58.0, 56.1, 18.2, 14.1 ppm. ESI-HRMS: m/z $[M-H]^-$ calcd. for $C_{56}H_{59}Cl_6N_2O_{13}$ 1177.2148 found 1177.2329.

^{1}H NMR ($CDCl_3$)

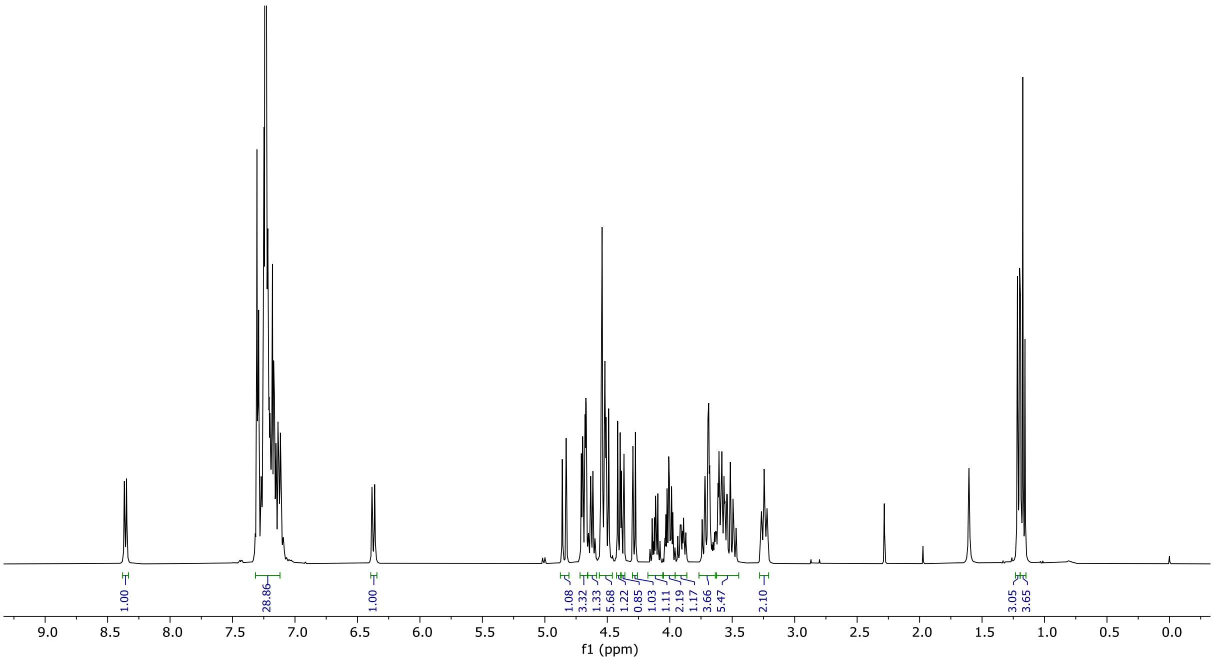

^{13}C APT NMR ($CDCl_3$)

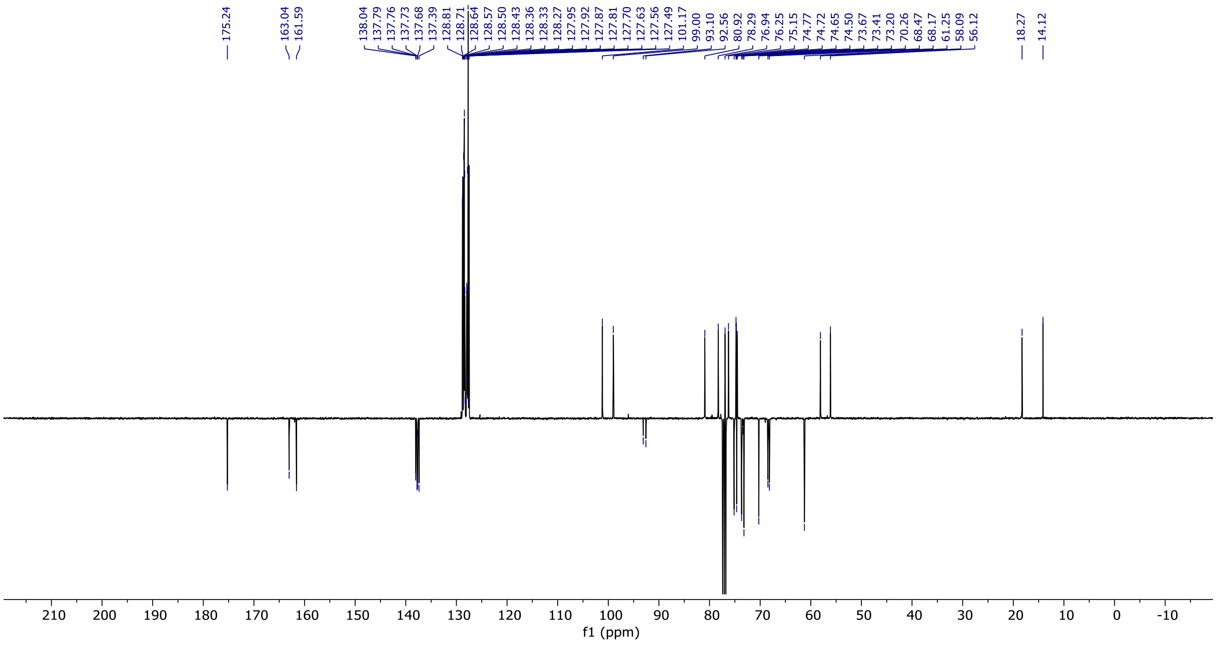

HSQC NMR ($CDCl_3$)

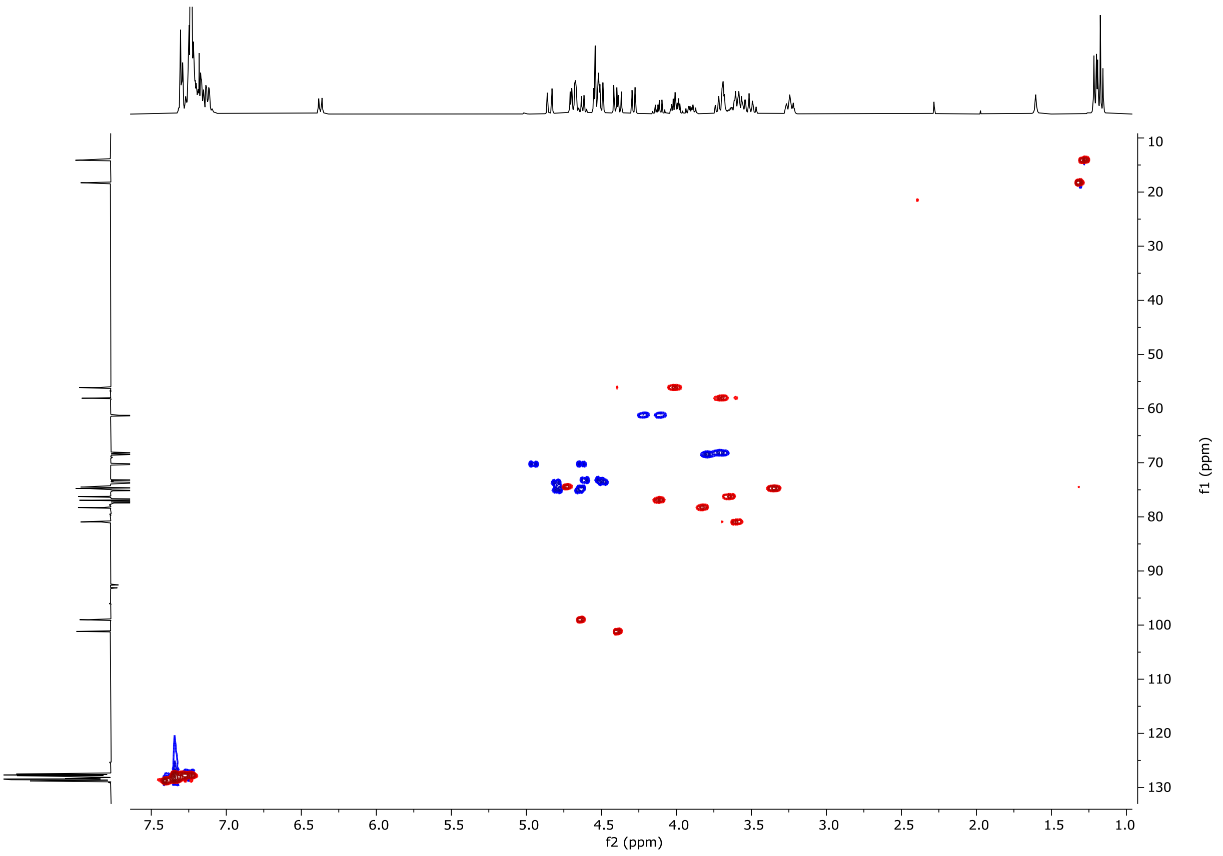

Synthesis of Benzyl 2-deoxy-3,4,6-tri-O-benzyl-2- trichloro-acetamido-β-D-glucopyranosyl-(1→4)-2-deoxy-3-O-[(R)-1`-carboxyethyl]-6-O-benzyl-2-trichloro-acetamido-β-D-glucopyrano-side ***4.8***

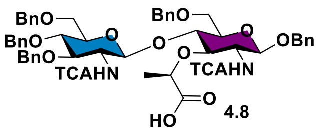

To a solution of protected disaccharide **4.7** (0.25 g, 1.30 mmol) in $THF/H_2O/MeOH$ (2:2:1, 10 mL), was added LiOH (15.0 mg, 0.64 mmol) and the mixture was stirred for 4 h. Then, the reaction was quenched with acetic acid (0.50 mL), diluted with ethyl acetate (50 mL), washed with water (2×10 mL) and NaCl solution (2×10 mL), dried over Na_2SO_4 and the solvent was removed under reduced pressure. The residue was purified by flash column chromatography using a mixture of dichloromethane/acetonitrile (5:1). The product was obtained as a white solid in 92% yield (0.22 g, 0.19 mmol). R_f (DCM/MeCN 2:1): 0.6. ^{1}H NMR (600 MHz, $CDCl_3$): δ 7.90 (d, J = 7.4 Hz, 1H), 7.41 – 7.19 (m, 25H), 6.52 (d, J = 7.7 Hz, 1H), 4.91 (d, J = 12.3 Hz, 1H), 4.78 (d, J = 11.1 Hz, 2H), 4.76 (d, J = 12.0 Hz, 1H), 4.70 (t, J = 6.9 Hz, 1H), 4.67 (d, J = 7.8 Hz, 1H), 4.65 – 4.60 (m, 2H), 4.60 – 4.58 (m, 1H), 4.57 – 4.54 (m, 2H), 4.52 – 4.46 (m, 2H), 4.11 (t, J = 8.5 Hz, 1H), 3.96 (dt, J = 10.1, 7.5 Hz, 1H), 3.80 – 3.73 (m, 4H), 3.72 – 3.62 (m, 4H), 3.43 (dt, J = 8.8, 3.2 Hz, 1H), 3.38 (ddd, J = 9.6, 4.0, 2.3 Hz, 1H), 1.34 (d, J = 7.0 Hz, 3H) ppm. ^{13}C NMR (151 MHz, $CDCl_3$): δ 162.6, 161.7, 137.9, 137.7, 137.5, 137.0, 128.7, 128.6, 128.5, 128.5, 128.4, 128.3, 128.2, 127.9, 127.9, 127.8, 127.8, 127.6, 127.6, 100.3, 99.1, 92.8, 92.4, 80.5, 78.3, 76.3, 75.8, 75.1, 74.7, 74.7, 74.5, 74.1, 73.6, 73.3, 70.6, 68.4, 68.2, 58.0, 55.8, 18.0 ppm. ESI-HRMS: m/z $[M-H]^-$ calcd. for $C_{54}H_{55}Cl_6N_2O_{13}$ 1149.1835 found 1149.1455.

^{1}H NMR ($CDCl_3$)

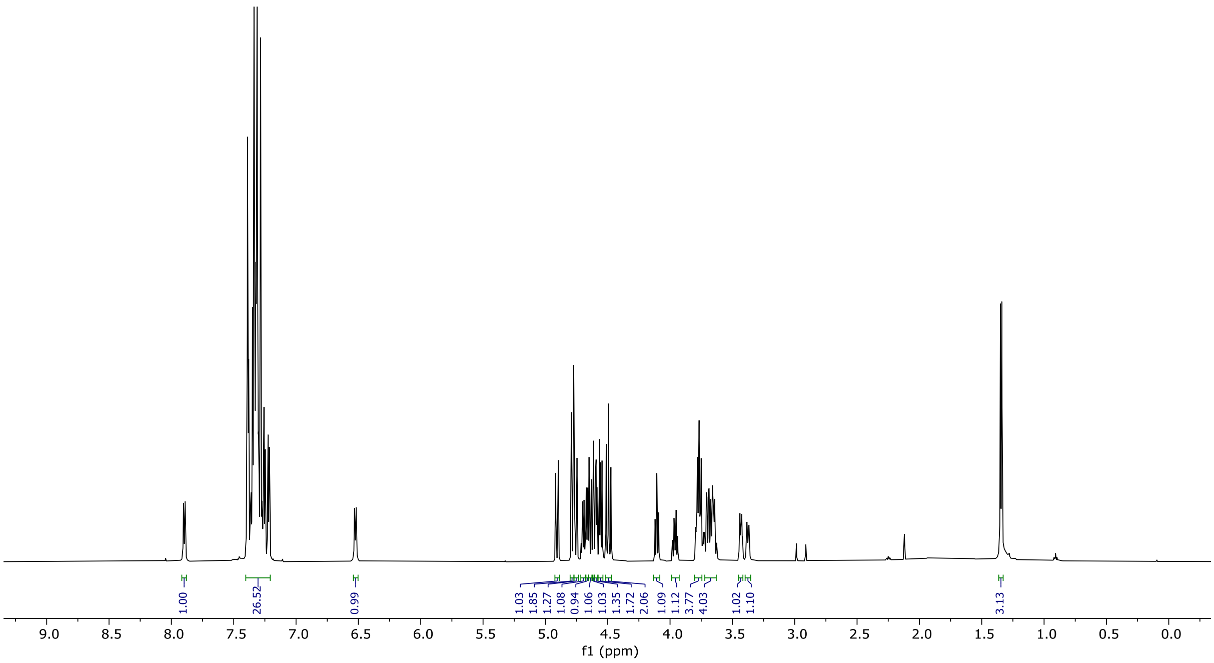

^{13}C APT NMR ($CDCl_3$)

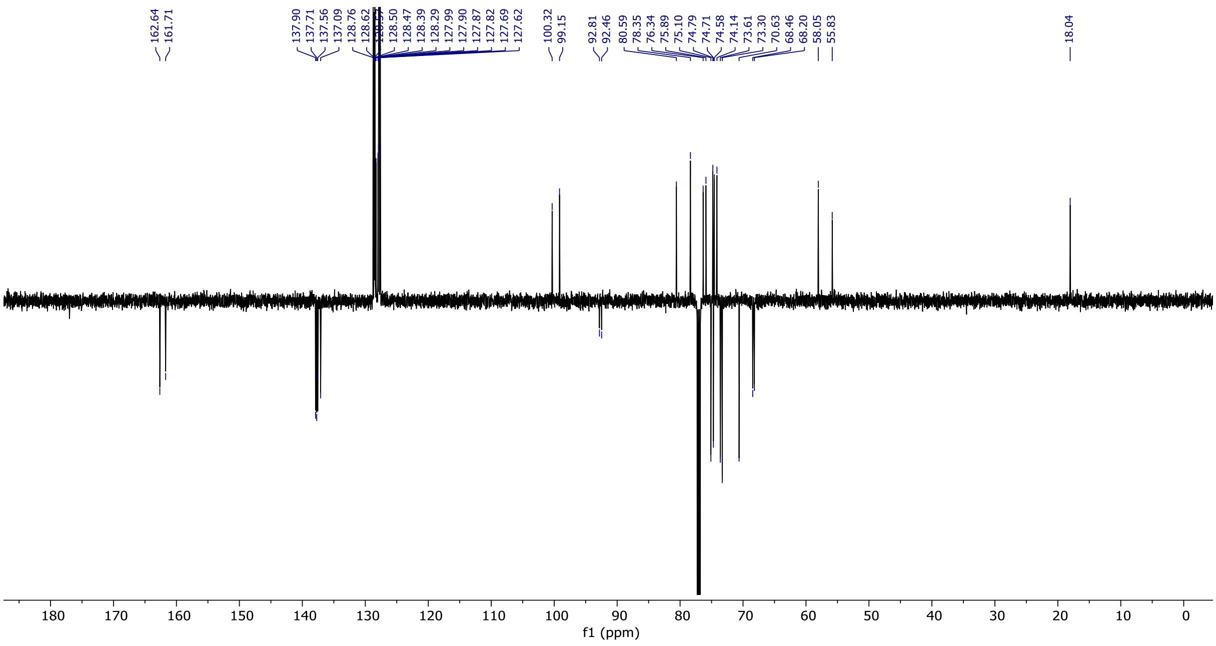

HSQC NMR ($CDCl_3$)

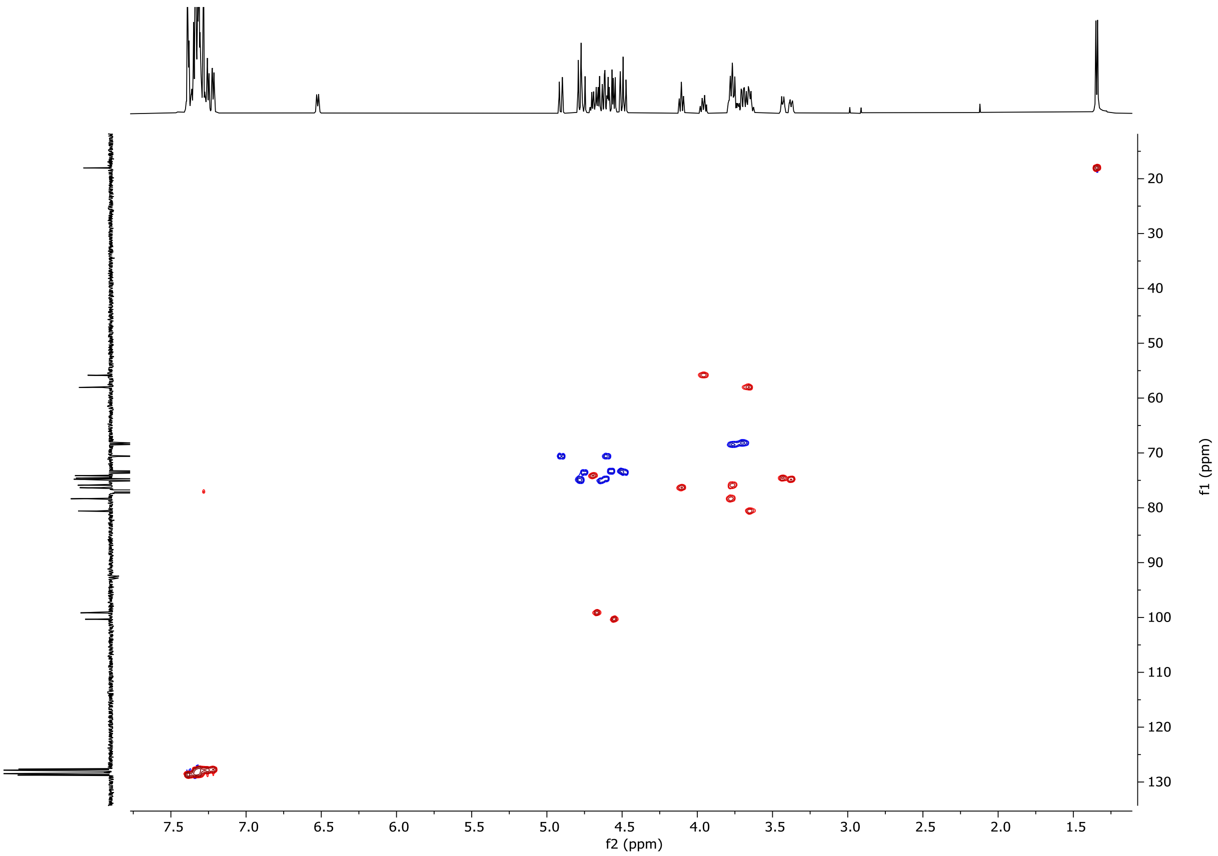

*Synthesis of 2-acetamido-2-deoxy-β-D-glucopyranosyl-(1→4)-2-acetamido-3-O-[(R)-1-carboxyethyl]-2-deoxy-β-D-glucopyranoside **4***

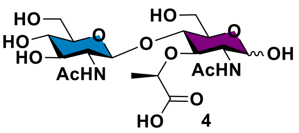

The perbenzylated disaccharide **4.8** (149 mg) was dissolved in THF/H_2O/AcOH/ (2:2:1, 2 mL) and 150 mg of Pd(C) were added. The resulting mixture was stirred under hydrogen atmosphere for 1 week. After filtering the catalyst out, the resulting crude was evaporated under reduced pressure until dryness. The crude product was purified using preparative reverse phase HPLC (Hypercarb column, 150 x 10 mm, 5 μm), flow rate of 3 mL/min with H_2O (0.1% formic acid) as eluents [isocratic (5 min), linear gradient to 20% ACN (30 min), linear gradient to 100% ACN (5 min)]. The pure compound was analyzed using an analytical HPLC (Hypercarb column, 150 x 4.6 mm, 3 μm) flow rate of 0.7 mL/min with H_2O (0.1% formic acid) as eluents [isocratic (5 min), linear gradient to 20% ACN (30 min), linear gradient to 100% ACN (5 min)]. The desired product was obtained after lyophilization as a white solid in 44% yield (28.0 mg, 0.05 mmol). ^1H NMR (400 MHz, D_2O): δ 8.38 (d, J = 9.5 Hz, 2H), 5.34 (d, J = 3.1 Hz, 1H), 4.73 – 4.60 (m, 1H), 4.50 (dd, J = 8.3, 3.5 Hz, 1H), 3.95 – 3.49 (m, 9H), 3.46 – 3.34 (m, 2H), 2.00 (d, J = 8.7 Hz, 6H), 1.42 (dd, J = 6.9, 3.5 Hz, 3H) ppm. ^{13}C NMR (101 MHz, D_2O): δ 174.5, 174.3, 100.2, 95.0, 89.9, 76.1, 75.9, 75.4, 74.9, 73.4, 71.0, 70.3, 61.1, 59.6, 56.0, 53.9, 21.9, 18.1 ppm. ESI-HRMS: m/z $[M-H]^-$ calcd. for $C_{19}H_{31}N_2O_{13}$ 495.1826 found 495.1844.

^{1}H NMR (D_2O)

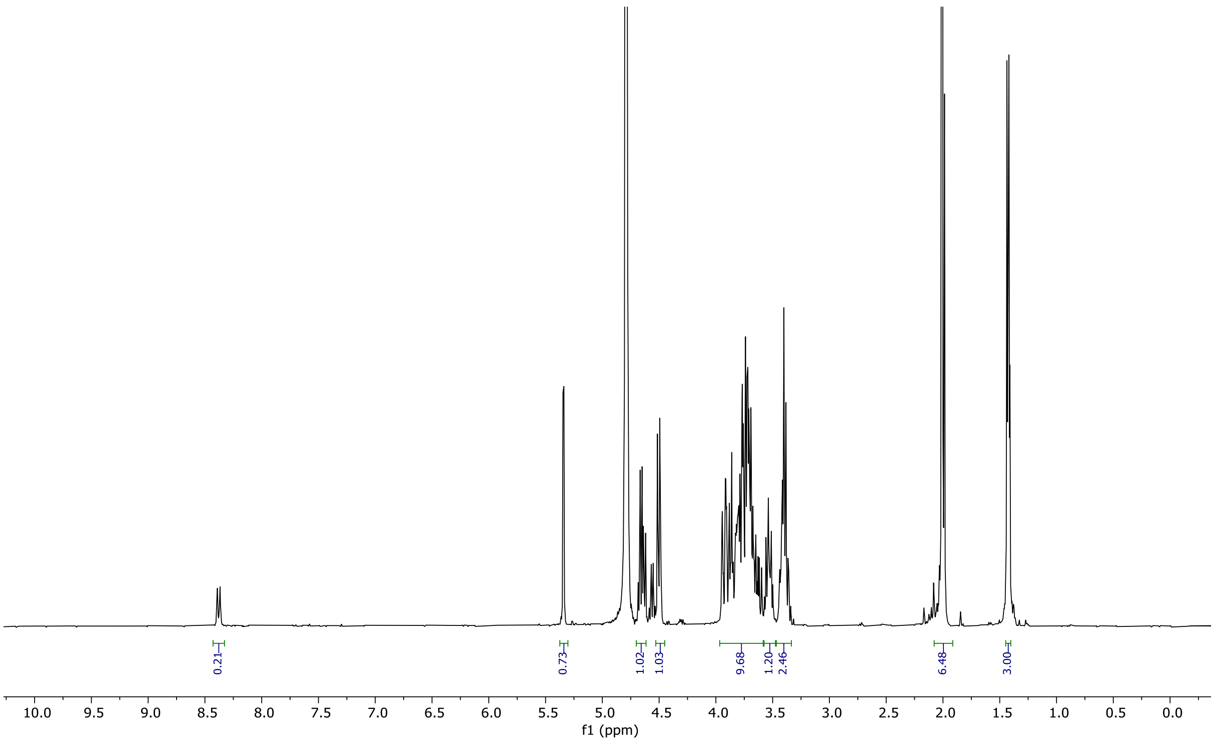

^{13}C NMR (D_2O)

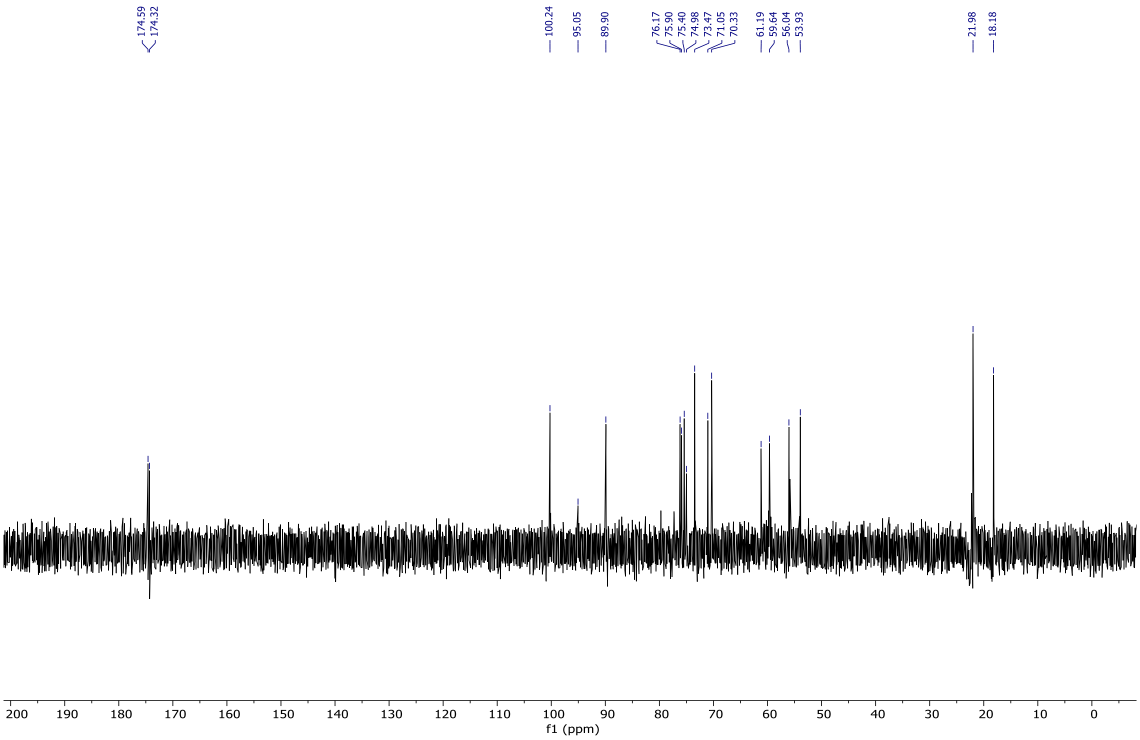

HSQC (D_2O)

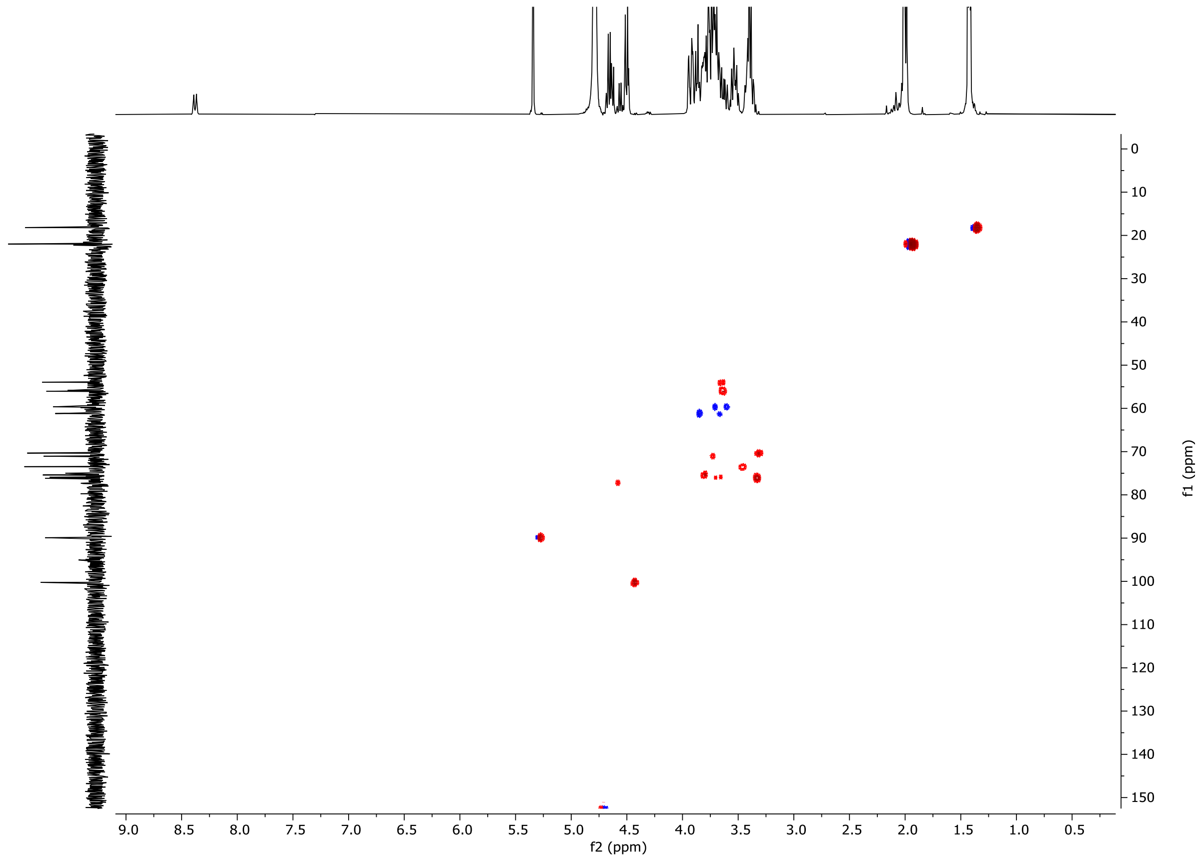

RP-HPLC (ELSD trace, method B, t_R = 22.5 min, t_R = 23.2 min of anomers)

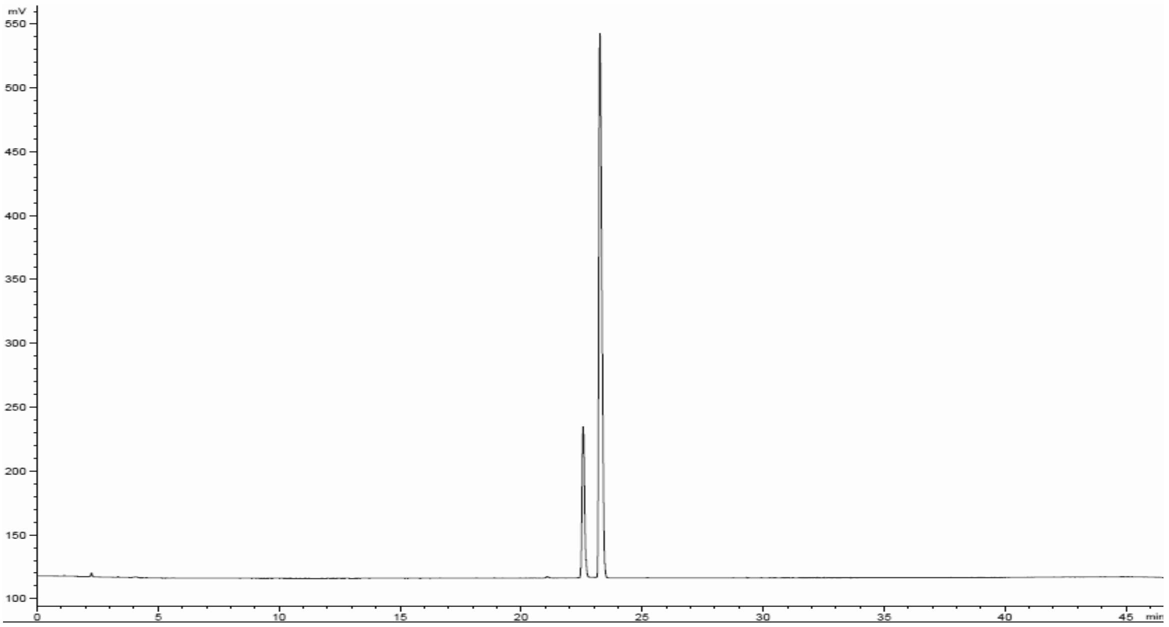

Synthesis of 2-acetamido-3-*O*-[(R)-1`-carboxyethyl]-2-deoxy-β-D-glucopyranosyl-(1→4)-2-acetamido-2-deoxy-β-D-glucopyranoside 5

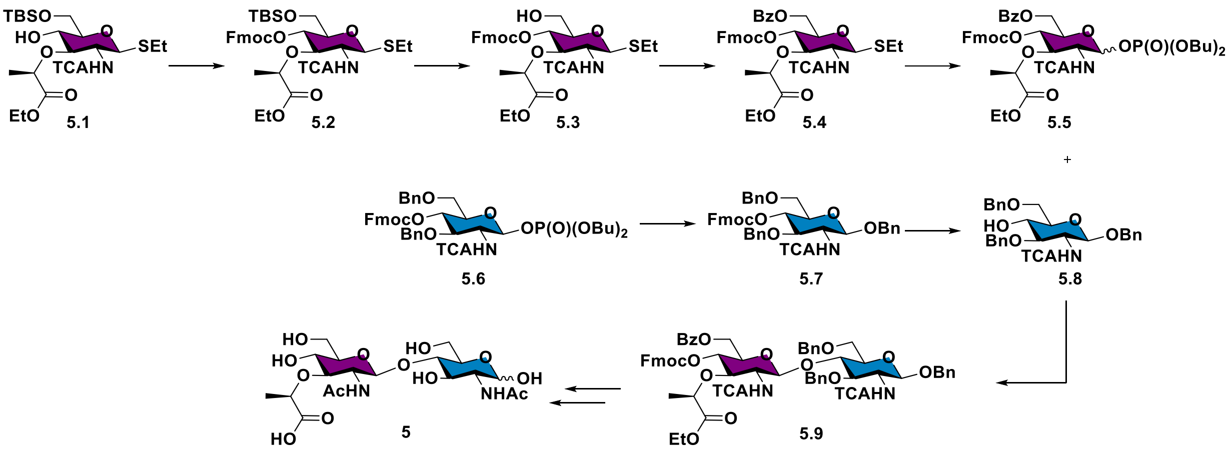

*Ethyl 6-O-tert-butyl dimethyl silyl-2-deoxy-3-O-((R)-1'-ethoxycarbonylethyl)-4-O-fluorenylme-thoxycarbonyl-1-thio-2-trichloroacetamido-β- D -glucopyranoside **5.2***

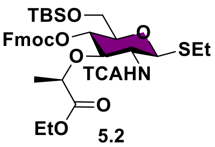

5.2

Building block **5.1**, purchaced from GlycoUniverse GmbH (2.87 g, 4.92 mmol, 1.00 equiv.) was dissolved in anhydr. DCM (25 mL). Then, pyridine (2.40 mL, 29.5 mmol, 6.00 equiv.) and FmocCl (2.55 g, 9.84 mmol, 2.00 equiv.) were added consecutively. The reaction mixture was stirred for 30 min at rt and washed with aq. 1 M HCl solution (50 mL) and NaCl (50 mL). The organic layer was dried over Na_2SO_4 and the solvent was removed under reduced pressure. The residue was purified by flash column chromatography using a mixture of hexane/ethyl acetate (4:1) as eluent. The product was obtained as white solid in 71% yield (2.82 g, 3.50 mmol). R_f (Hex/EA 5:1): 0.57; ^{1}H NMR (400 MHz, $CDCl_3$): δ 9.37 (d, *J* = 4.2 Hz, 1H), 8.14 (d, *J* = 7.2 Hz, 1H), 7.79 (dt, *J* = 7.5, 2.6 Hz, 2H), 7.66 – 7.55 (m, 2H), 7.43 (td, *J* = 7.5, 4.1 Hz, 2H), 7.33 (m, *J* = 8.7, 7.5, 4.5, 1.2 Hz, 2H), 5.11 (dd, *J* = 10.1, 9.2 Hz, 1H), 4.89 (t, *J* = 9.5 Hz, 1H), 4.62 (d, *J* = 10.0 Hz, 1H), 4.58 – 4.10 (m, 6H), 4.09 – 3.96 (m, 1H), 3.86 (td, *J* = 10.2, 7.2 Hz, 1H), 3.79 – 3.70 (m, 2H), 3.52 (ddd, *J* = 9.8, 4.9, 3.2 Hz, 1H), 2.79 – 2.66 (m, 2H), 1.32 – 1.22 (m, 6H), 0.88 (d, *J* = 1.0 Hz, 9H), 0.08 – 0.03 (m, 6H) ppm; ^{13}C NMR (101 MHz, $CDCl_3$): δ 174.5, 173.7, 163.1, 162.8, 154.1, 153.9, 143.4, 143.0, 141.4, 128.1, 127.3, 125.1, 124.9, 120.3, 92.7, 91.3, 84.5, 78.9, 78.4, 77.3, 75.6, 75.2, 74.0, 73.5, 70.5, 62.8, 62.2, 61.7, 61.4, 57.1, 56.2, 46.8, 33.0, 25.9, 24.3, 18.9, 18.4, 15., 14.6, 14.3, -5.24 ppm; IR (neat) νmax: 3300, 2253, 1444, 1418, 1375, 1039 cm^{-1};ESI-HRMS: m/z $[M-Na]^+$ calcd. for $C_{36}H_{48}Cl_3NO_9SSiNa$ 826.1792 found 826.1779.

^{1}H NMR ($CDCl_3$)

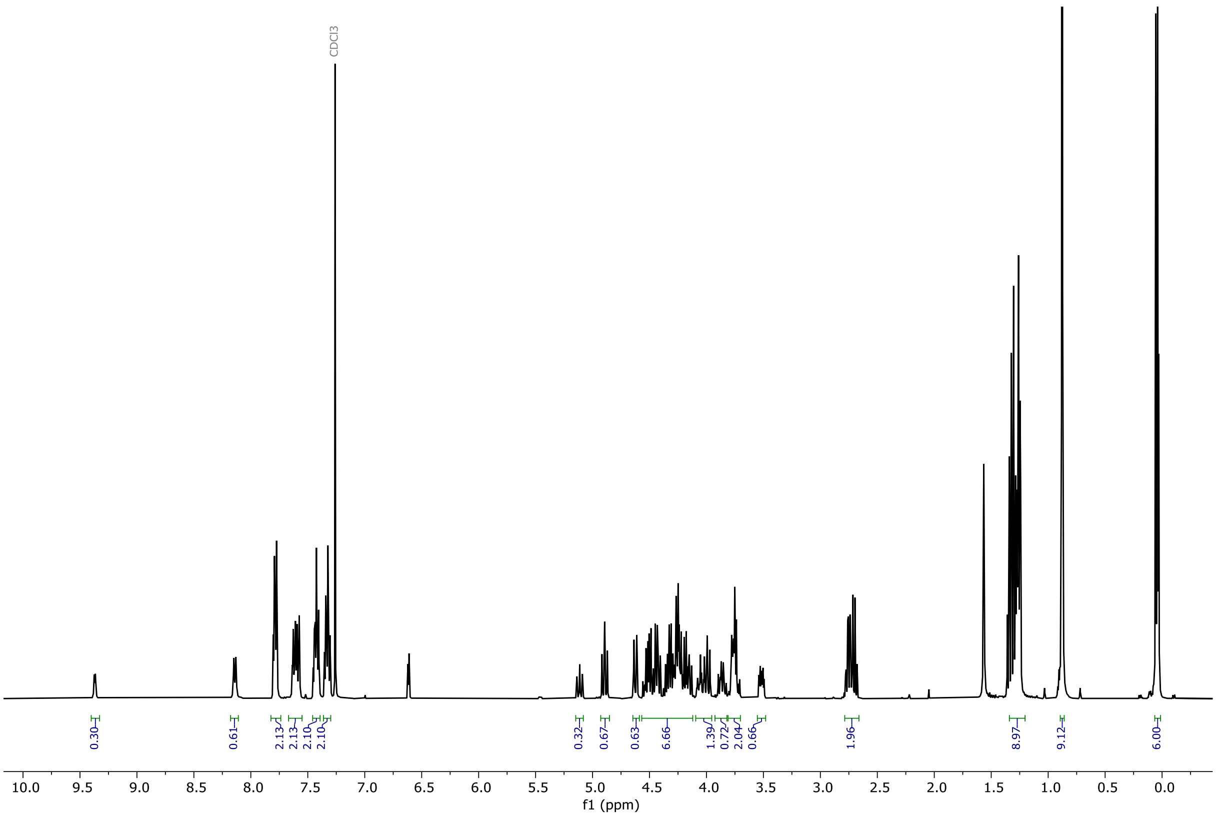

^{13}C NMR ($CDCl_3$)

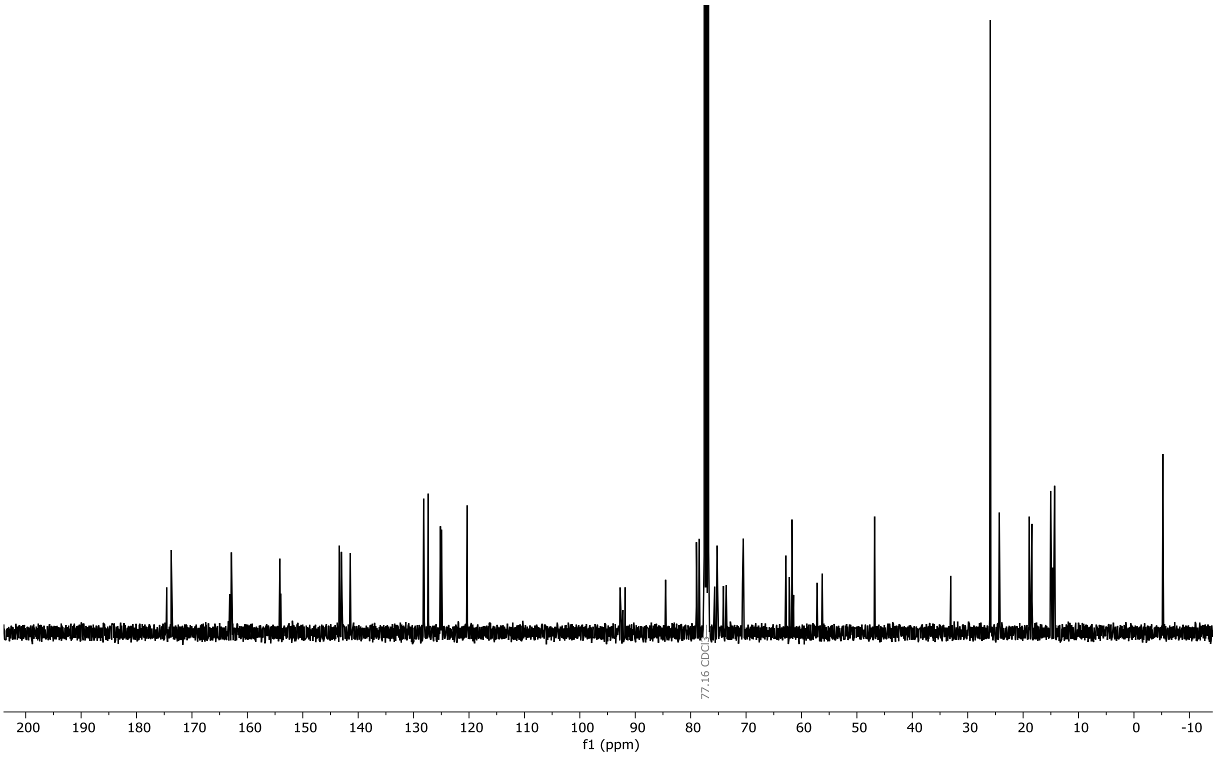

HSQC ($CDCl_3$)

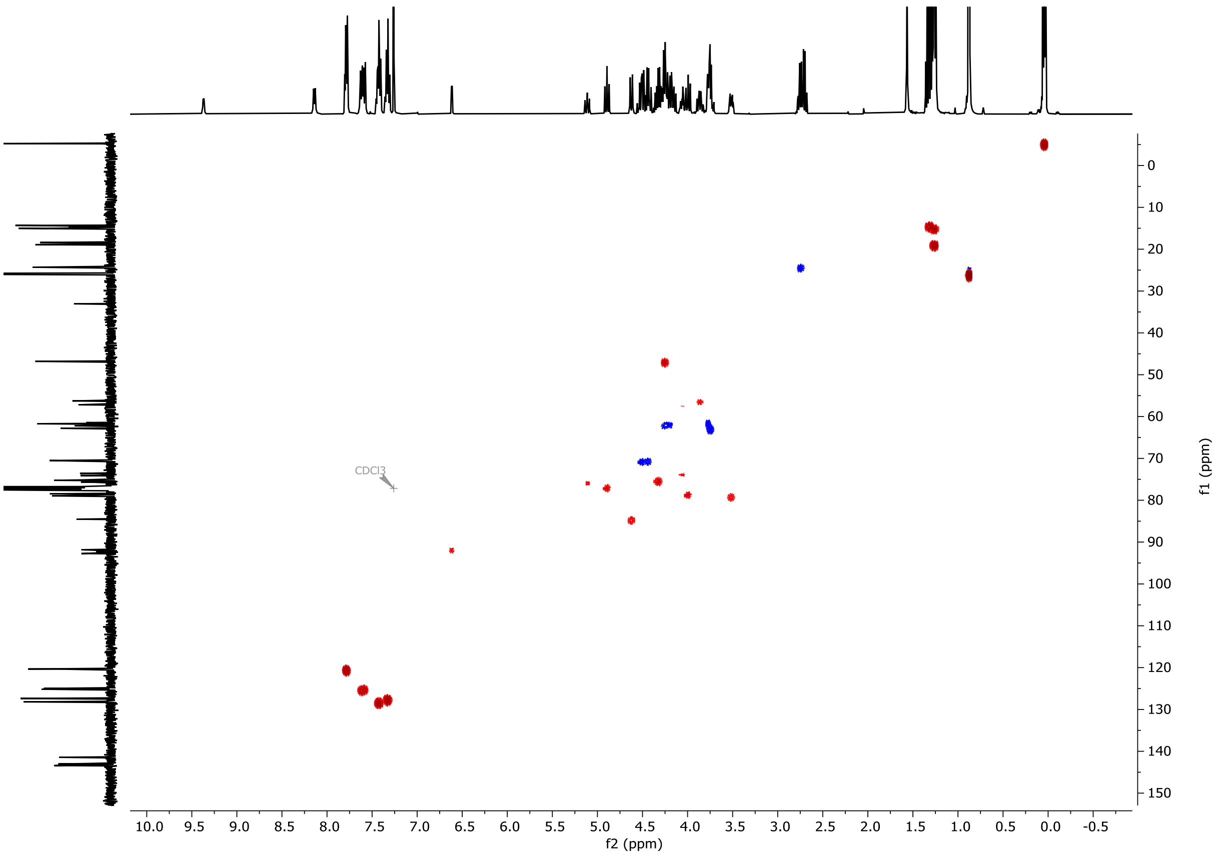

Ethyl 2-deoxy-3-O-((R)-1'-ethoxycarbonyl ethyl)-4-O-fluorenylmethoxycarbonyl-1-thio-2-trichloroaceta-mido-β-D-glucopyranoside ***5.3***

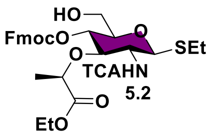

5.2 (2.80 g, 3.48 mmol, 1.00 equiv.) was dissolved in a solvent mixture of dichloromethane: methanol (2:1, 60 mL). AcCl (0.12 mL, 1.74 mmol, 0.50 equiv.) was added and the resulted mixture was stirred at rt for 30 min. The reaction mixture was diluted with dichloromethane (100 mL) and washed with $NaHCO_3$ (100 mL). The aqueous layer was re-extracted with dichloromethane (2 × 40 mL), and the combined organic layers were dried over ($MgSO_4$), and the solvent was removed under reduced pressure. The residue was purified by flash column chromatography using a mixture of hexane/ethyl acetate (2:1) as eluent. The product was obtained as a white foam in 99% yield (2.38 mg, 3.44 mmol). R_f (Hex/EA 2:1): 0.21; ^{1}H NMR (400 MHz, DMSO): δ 8.93 (d, J = 8.9 Hz, 1H), 7.89 (ddt, J = 7.6, 4.1, 1.0 Hz, 2H), 7.70 – 7.60 (m, 2H), 7.47 – 7.27 (m, 4H), 4.81 (t, J = 5.5 Hz, 1H), 4.71 – 4.57 (m, 2H), 4.57 – 4.49 (m, 2H), 4.30 (t, J = 5.5 Hz, 1H), 4.09 – 3.94 (m, 3H), 3.85 – 3.68 (m, 2H), 3.46 – 3.38 (m, 2H), 3.32 (s, 1H), 2.72 – 2.51 (m, 2H), 1.15 (td, J = 7.3, 4.6 Hz, 6H), 0.94 (d, J = 6.7 Hz, 3H) ppm; ^{13}C NMR (101 MHz, DMSO): δ 171.5, 161.4, 161.3, 154.2, 143.8, 143.6, 141.3, 128.2, 127.5, 125.3, 125.1, 120.6, 93.3, 83.6, 79.7, 78.4, 75.9, 74.5, 69.2, 60.9, 60.8, 56.2, 46.7, 23.8, 18.5, 15.2, 14.3 ppm. IR (neat) νmax: 3300, 2253, 1442, 1418, 1375, 1039, 749 cm^{-1}; ESI-HRMS: m/z $[M-Na]^+$ calcd. for $C_{30}H_{34}Cl_3NO_9SNa$ 712.0892 found 712.0910.

^{1}H NMR (DMSO)

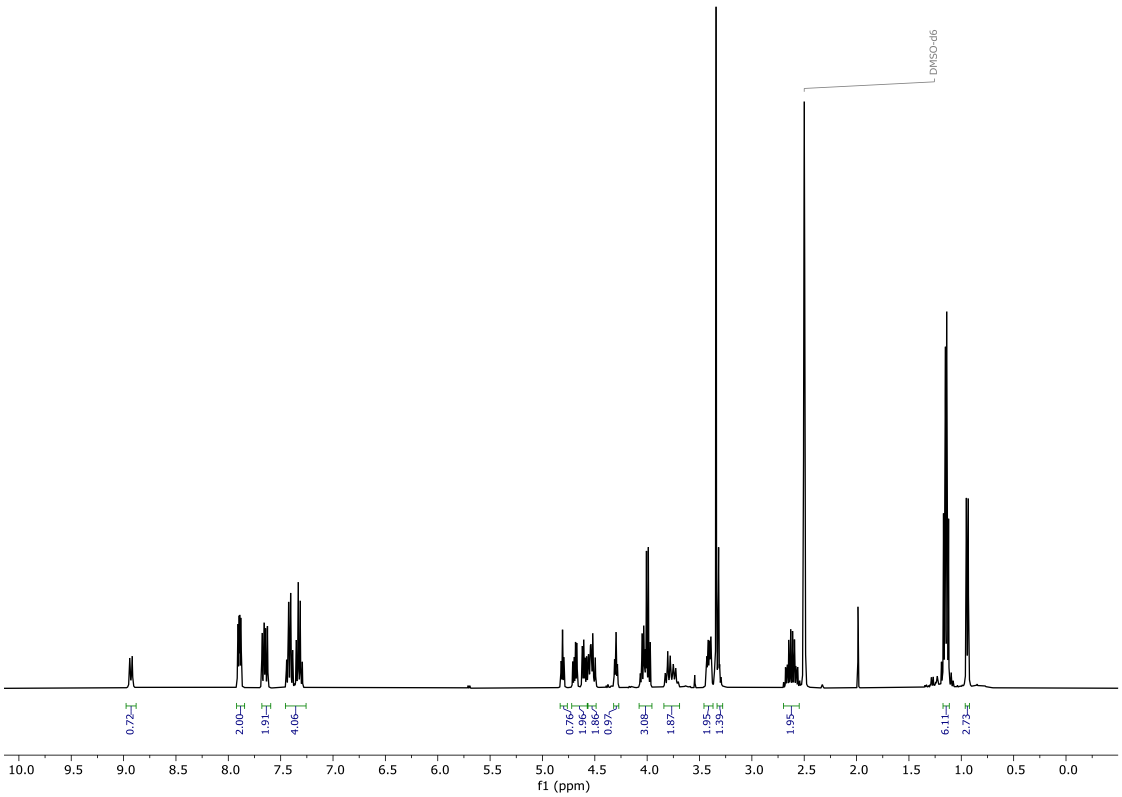

^{13}C NMR (DMSO)

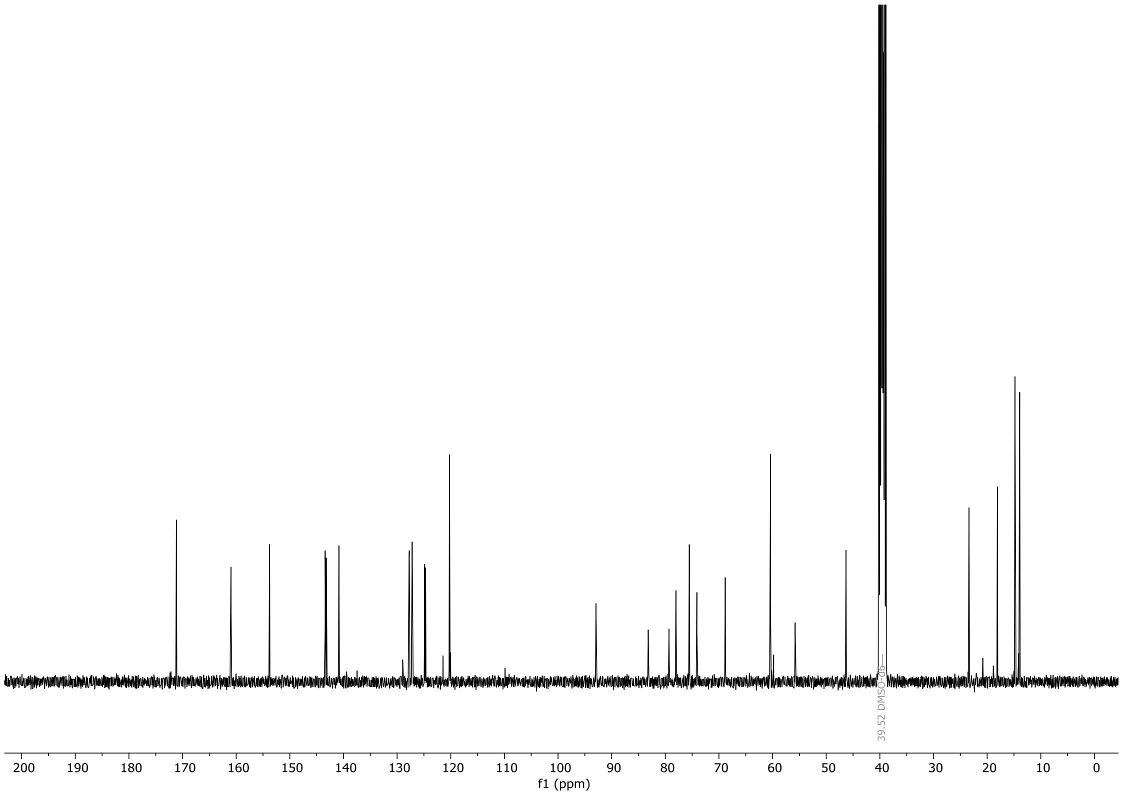

HSQC (DMSO)

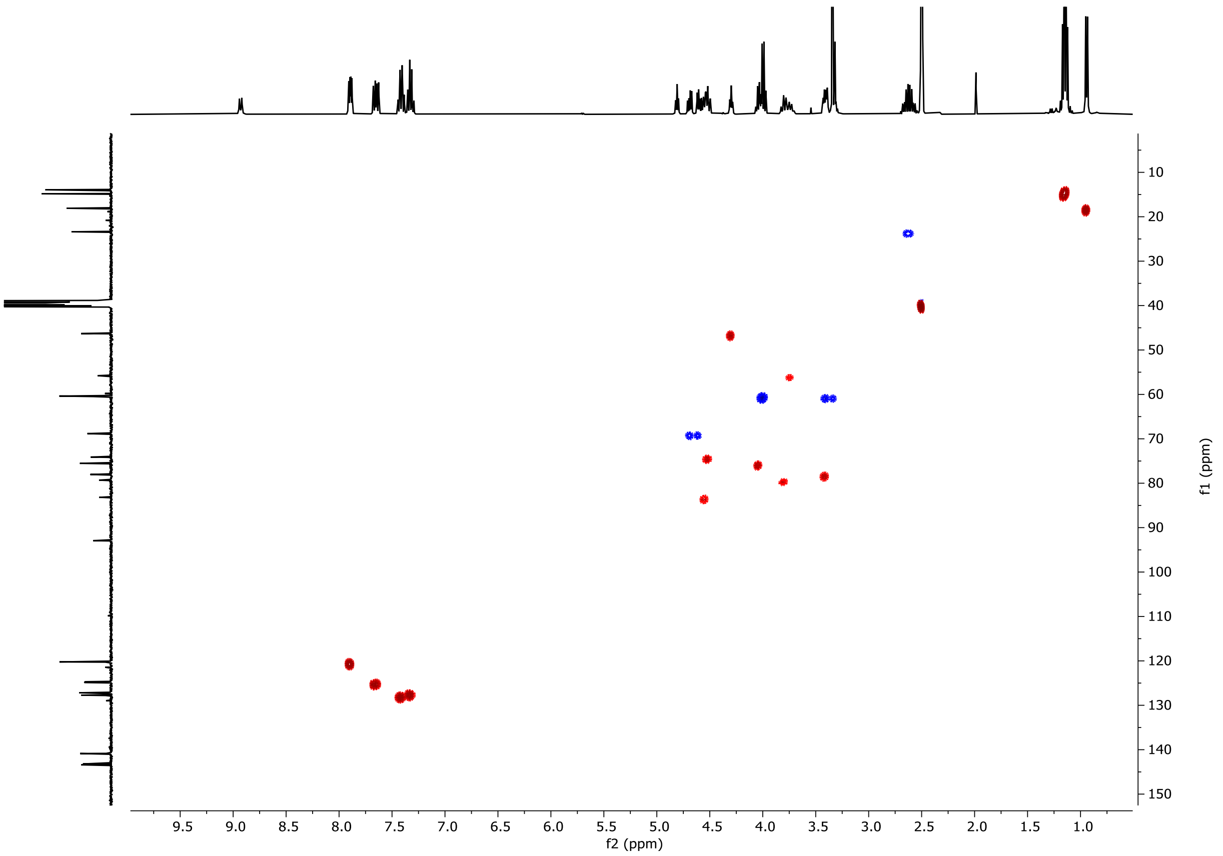

Ethyl 2-deoxy-3-O-((R)-1'-ethoxycarbonyl ethyl)-4-O-fluorenylmethoxycarbonyl-6-O-benzoyl-1-thio-2-tri-chloroacetamido-β-D-glucopyranoside ***5.4***

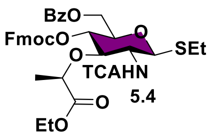

5.3 (2.30 g, 3.32 mmol, 1.00 equiv.) was dissolved in anhydr. Pyridine (20 mL) under argon atmosphere and the solution was cooled to 0 °C. BzCl (1.15 mL, 10.0 mmol, 3.00 equiv.) was added dropwise and the mixture was left to react overnight at rt. After completion, the mixture was poured into iced water; the precipitate was filtered off and washed with water. The solid was dissolved in DCM (20 mL) and washed with water (40 mL). Then, the organic layer was washed with hydrochloric acid (1 M, 150 mL), $NaHCO_3$ solution (100 mL), and water (100 mL). The organic layer was dried over Na_2SO_4 and the solvent was removed under reduced pressure. The residue was purified by flash column chromatography using a mixture of hexane/ethyl acetate (2:1) as eluent. The product was obtained as a white foam in 94% yield (2.49 mg, 3.13 mmol). R_f (Hex/EA 2:1): 0.71; ^{1}H NMR (400 MHz, DMSO): δ 8.98 (d, *J* = 9.0 Hz, 1H), 8.00 (dt, *J* = 7.1, 1.4 Hz, 2H), 7.90 – 7.85 (m, 2H), 7.71 – 7.65 (m, 1H), 7.64 – 7.53 (m, 4H), 7.39 (t, *J* = 7.5 Hz, 2H), 7.32 – 7.21 (m, 3H), 4.74 (t, *J* = 9.5 Hz, 1H), 4.64 (dt, *J* = 10.7, 3.0 Hz, 2H), 4.57 (dd, *J* = 10.7, 5.7 Hz, 1H), 4.37 (dd, *J* = 12.3, 2.5 Hz, 1H), 4.25 (t, *J* = 5.5 Hz, 1H), 4.18 (dd, *J* = 12.3, 4.5 Hz, 1H), 4.08 (q, *J* = 6.7 Hz, 1H), 4.01 (q, *J* = 7.1 Hz, 2H), 3.91 – 3.84 (m, 2H), 3.84 – 3.78 (m, 1H), 2.68 – 2.52 (m, 2H), 1.13 (dt, *J* = 12.6, 7.2 Hz, 6H), 0.95 (d, *J* = 6.7 Hz, 3H); ^{13}C NMR (101 MHz, DMSO): δ 171.0, 165.3, 161.0, 153.7, 143.3, 143.1, 140.8, 133.5, 129.4, 129.2, 128.8, 127.7, 127.1, 127.0, 124.8, 124.6, 120.2, 120.1, 92.8, 83.4, 78.9, 75.6, 74.4, 73.6, 68.8, 62.6, 60.4, 59.8, 55.6, 46.3, 40.20, 23.5, 20.8, 18.0, 15.1, 14.1, 13.9 ppm; IR (neat) νmax: 2994, 2253, 1442, 1418, 1375, 1039, 749 cm^{-1}; ESI-HRMS: m/z [M-Na]$^+$ calcd. for $C_{37}H_{38}Cl_3NO_{10}SNa$ 816.1192 found 816.1151.

^{1}H NMR (DMSO)

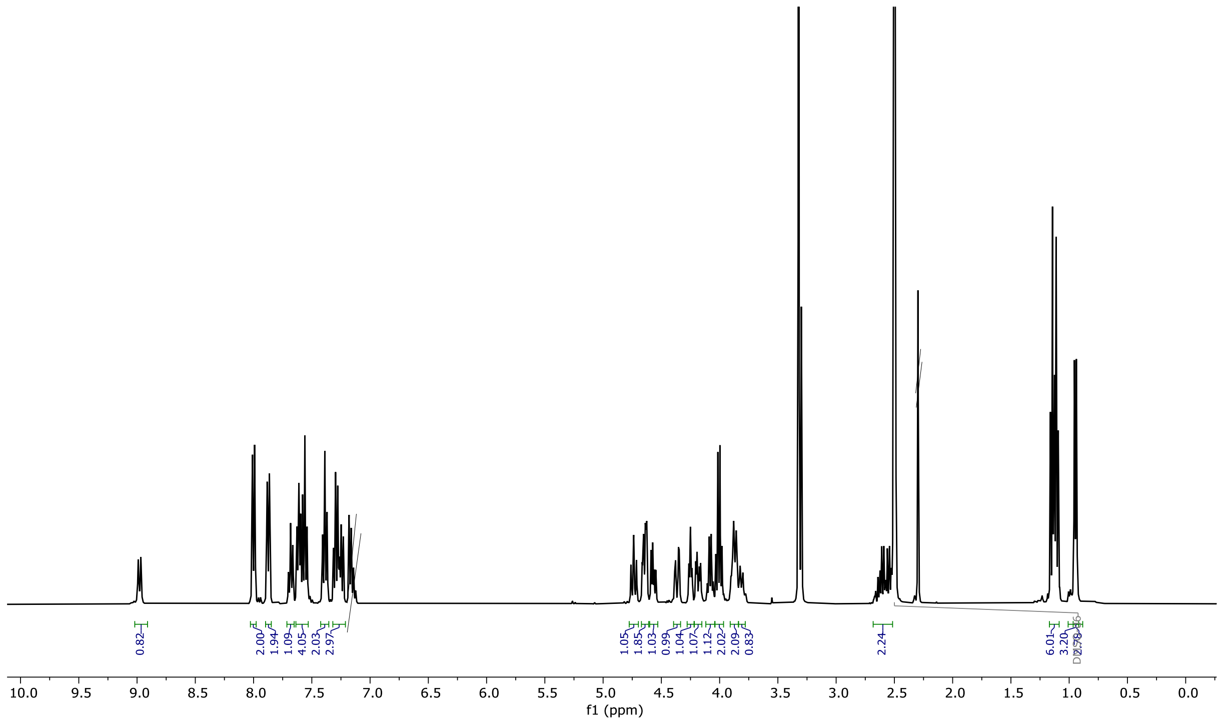

^{13}C NMR (DMSO)

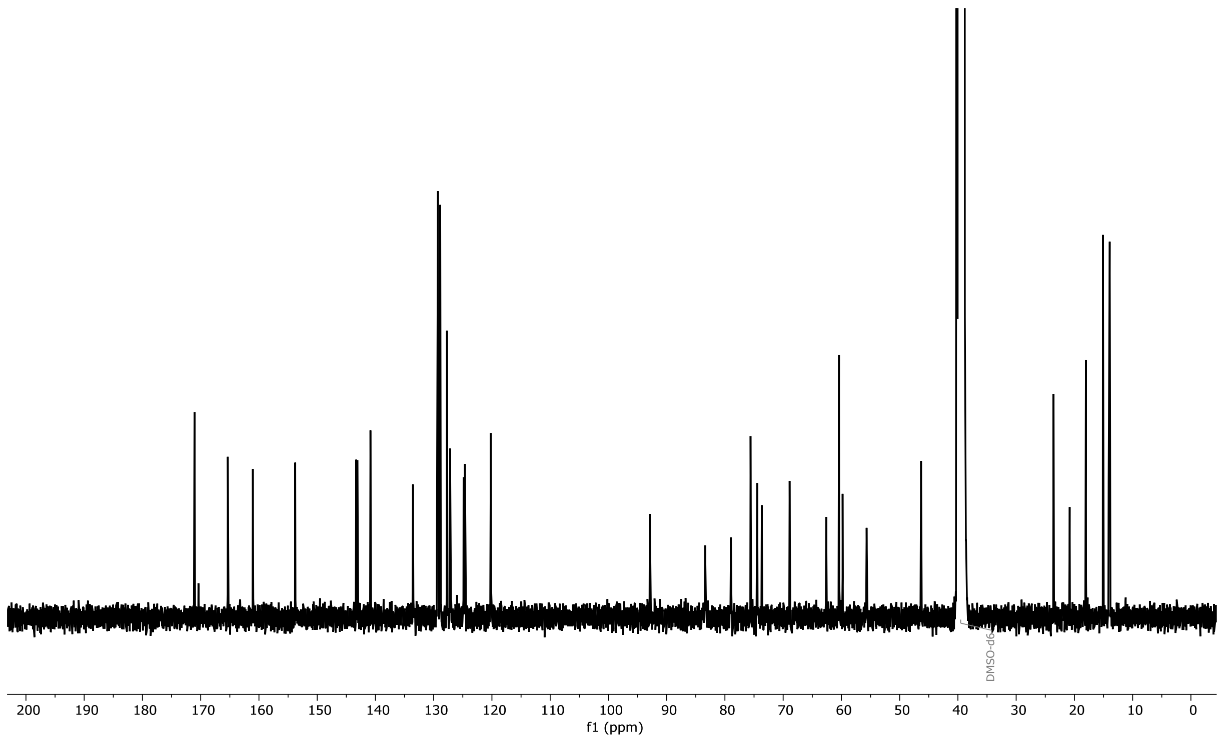

HSQC(DMSO)

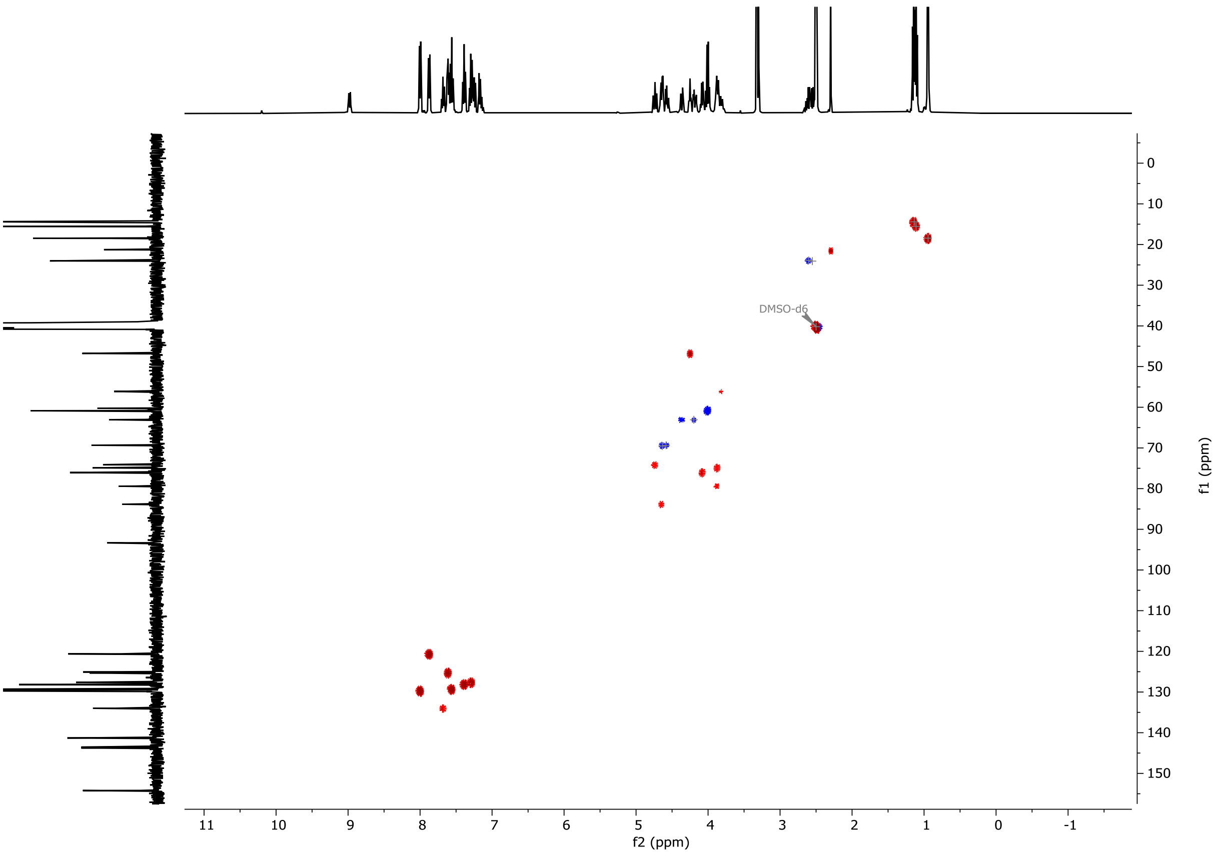

Dibutylphosphoryloxy 2-deoxy-3-O-((R)-1'-ethoxycarbonyl ethyl)-4-O-fluorenylmethoxycarbonyl-6-O-benzoyl-2-trichloroacetamido-β-glucopyranoside **5.5**

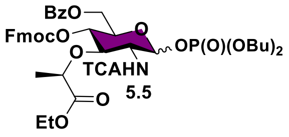

Dibutyl hydrogen phosphate (0.50 mL, 2.52 mmol, 2.00 equiv.) was added to a round-bottom flask containing activated 4Å molecular sieves anhydrous DCM (8 mL) and left stirring for 1.5 h. The molecular sieves were allowed to settle and the supernatant (8 mL) was added to a solution of the **5.4** (1.00 g, 1.26 mmol, 1.00 equiv.) in anhydrous DCM (8 mL). The mixture was cooled down to 0 °C, and *N*-iodosuccinimide (NIS) (368 mg, 1.64 mmol, 1.30 equiv.) and triflic acid (33.0 µL, 0.38 mmol, 0.30 equiv.) were added. The reaction was stirred for 1 h and then quenched with $NaHCO_3$ (5 mL). The organic layer was washed with $Na_2S_2O_3$ (30 mL) and water (30 mL), dried with Na_2SO_4, filtered and the solvent was removed under reduced pressure. The crude product was purified by flash column chromatography using a mixture of hexane/ethyl acetate (2:1). The α-anomer was obtained as a white foam in 60% yield (703 mg, 0.745 mmol), while the β-anomer a white foam in 17% yield (200 mg, 0.212 mmol). IR (neat) νmax: 2981, 2292, 2253, 1442, 1424, 1375, 1039, 749 cm^{-1}; ESI-HRMS: m/z $[M-Na]^+$ calcd. for $C_{43}H_{51}Cl_3NO_{14}PNa$ 964.1992 found 964.1979.

α-anomer: R_f (Hex/EA 2:1): 0.37; ^{1}H NMR (400 MHz, DMSO): δ 9.25 (d, *J* = 5.5 Hz, 1H), 8.04 – 7.99 (m, 2H), 7.88 (dq, *J* = 7.6, 1.0 Hz, 2H), 7.72 – 7.62 (m, 3H), 7.55 (t, *J* = 7.7 Hz, 2H), 7.44 – 7.36 (m, 2H), 7.35 – 7.27 (m, 2H), 5.73 (dd, *J* = 6.4, 3.1 Hz, 1H), 5.03 – 4.97 (m, 1H), 4.65 (m, 2H), 4.36 – 4.14 (m, 6H), 4.14 – 4.02 (m, 3H), 4.01 – 3.85 (m, 4H), 1.49 (m, 4H), 1.31 – 1.23 (m, 4H), 1.21 (t, *J* = 7.1 Hz, 3H), 1.05 (d, *J* = 6.9 Hz, 3H), 0.82 (dt, *J* = 13.3, 7.4 Hz, 6H) ppm; ^{13}C NMR (100 MHz, DMSO) δ 173.4, 165.3, 162.1, 153.6, 143.3, 143.0, 140.9, 133.6, 129.4, 129.2, 128.8,

127.7, 127.7, 127.2, 127.1, 124.8, 124.6, 120.2, 120.2, 93.5, 93.4, 92.0, 74.6, 74.5, 72.9, 69.2, 67.4, 67.4, 67.2, 67.1, 62.2, 61.1, 55.1, 55.0, 46.3, 31.6, 31.6, 31.6, 31.5, 18.3, 18.1, 13.9, 13.4, 13.3 ppm.

^{1}H NMR (DMSO)

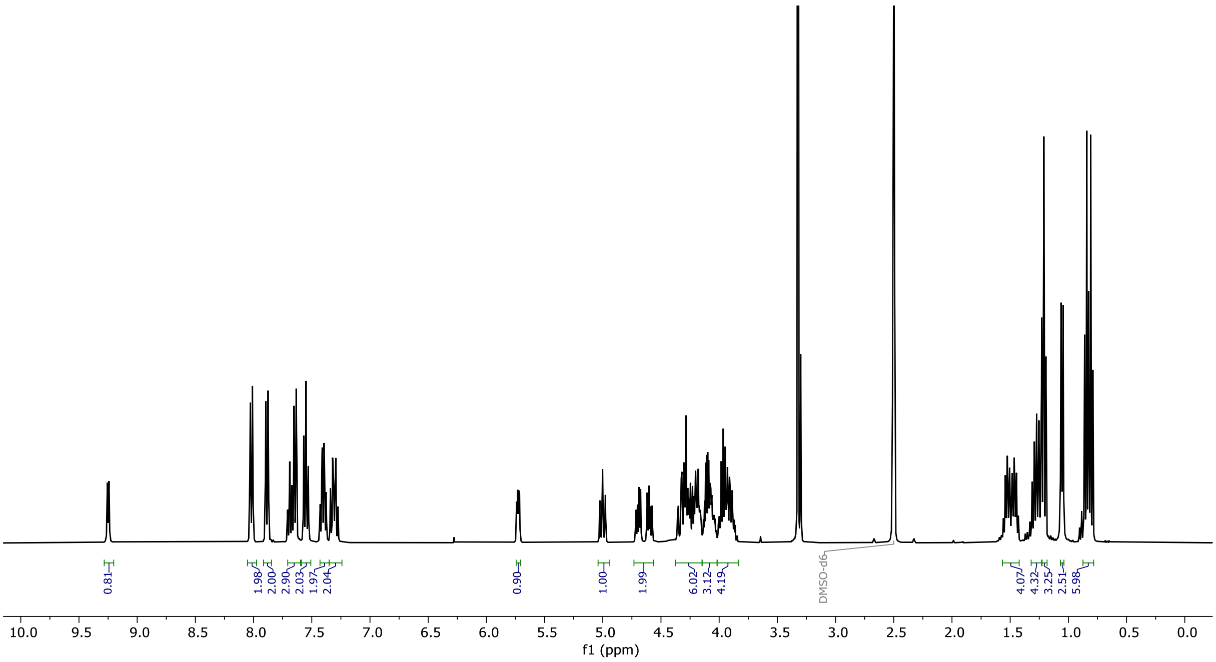

^{13}C NMR (DMSO)

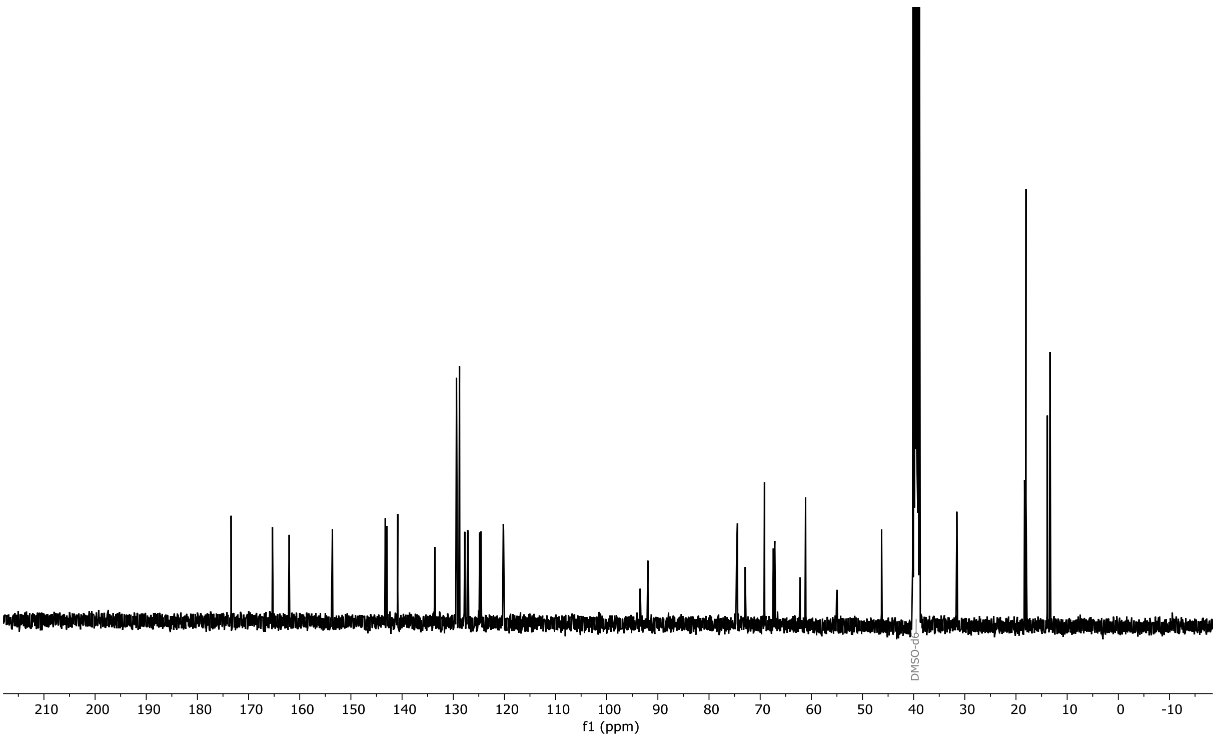

HSQC (DMSO)

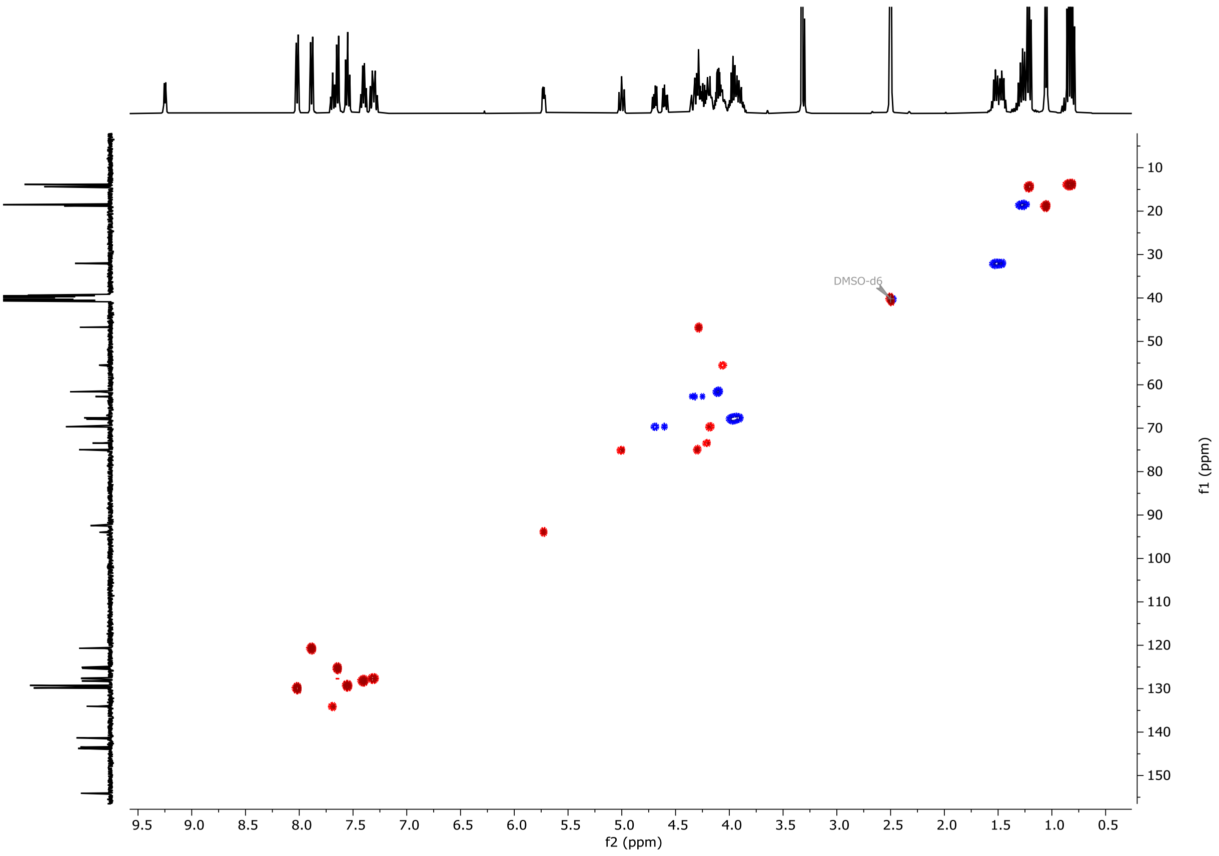

β-anomer: R_f (Hex/EA 2:1): 0.14; ^{1}H NMR (400 MHz, DMSO): δ 9.10 (d, *J* = 8.9 Hz, 1H), 8.05 – 7.96 (m, 2H), 7.90 – 7.84 (m, 2H), 7.73 – 7.66 (m, 1H), 7.65 – 7.60 (m, 2H), 7.56 (t, *J* = 7.7 Hz, 2H), 7.38 (t, *J* = 7.5 Hz, 2H), 7.28 (qd, *J* = 7.5, 1.2 Hz, 2H), 5.21 (dd, *J* = 8.1, 6.8 Hz, 1H), 4.81 (dd, *J* = 10.0, 9.0 Hz, 1H), 4.66 (ddd, *J* = 34.1, 10.7, 5.3 Hz, 2H), 4.38 (dd, *J* = 12.5, 2.4 Hz, 1H), 4.27 (t, *J* = 5.3 Hz, 1H), 4.18 (dd, *J* = 12.6, 4.4 Hz, 1H), 4.08 (q, *J* = 6.7 Hz, 1H), 4.05 – 3.93 (m, 4H), 3.93 – 3.81 (m, 5H), 1.54 – 1.35 (m, 4H), 1.32 – 1.09 (m, 7H), 0.94 (d, *J* = 6.7 Hz, 3H), 0.82 (t, *J* = 7.4 Hz, 3H), 0.71 (t, *J* = 7.4 Hz, 3H) ppm; ^{13}C NMR (100 MHz, DMSO) δ 171.5, 165.8, 162.0, 154.1, 143.7, 143.6, 141.3, 141.3, 134.0, 129.7, 129.3, 128.2, 127.6, 127.5, 125.3, 125.0, 120.7, 120.6, 96.5, 93.2, 77.3, 75.8, 73.6, 71.6, 69.4, 67.8, 67.7, 67.6, 67.5, 62.6, 60.9, 57.3, 57.2, 46.8, 32.0, 31.9, 31.8, 18.5, 18.4, 14.4, 13.8, 13.7 ppm.

^{1}H NMR (DMSO)

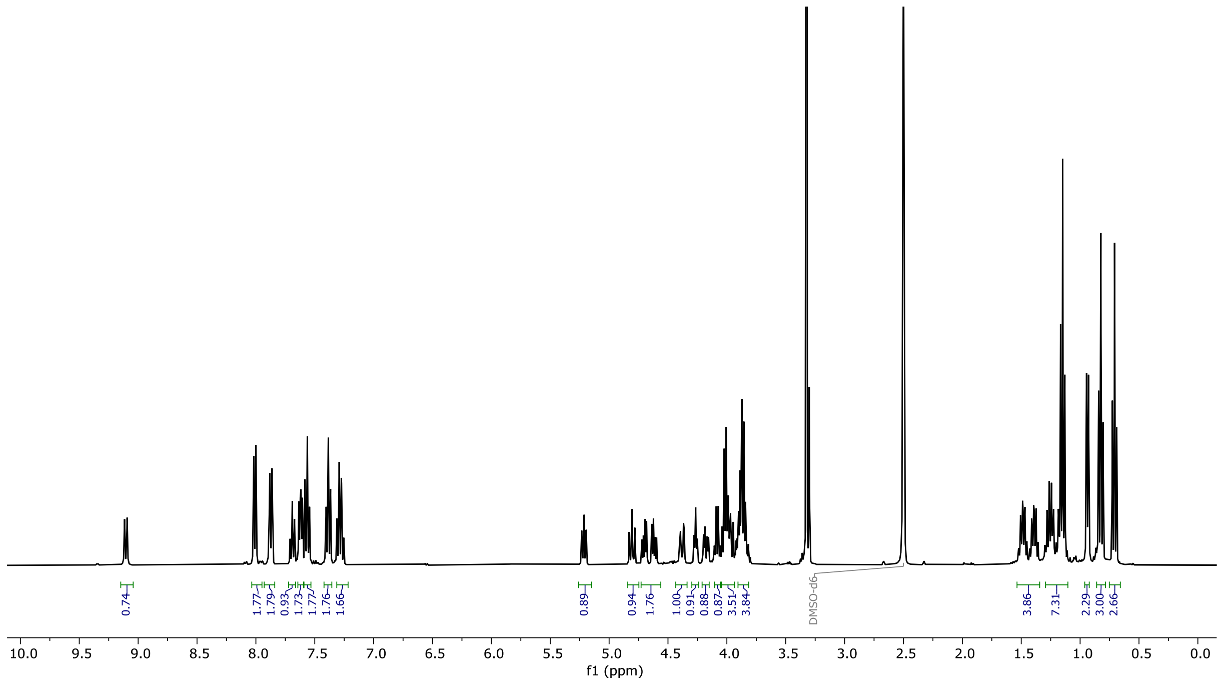

^{13}C NMR (DMSO)

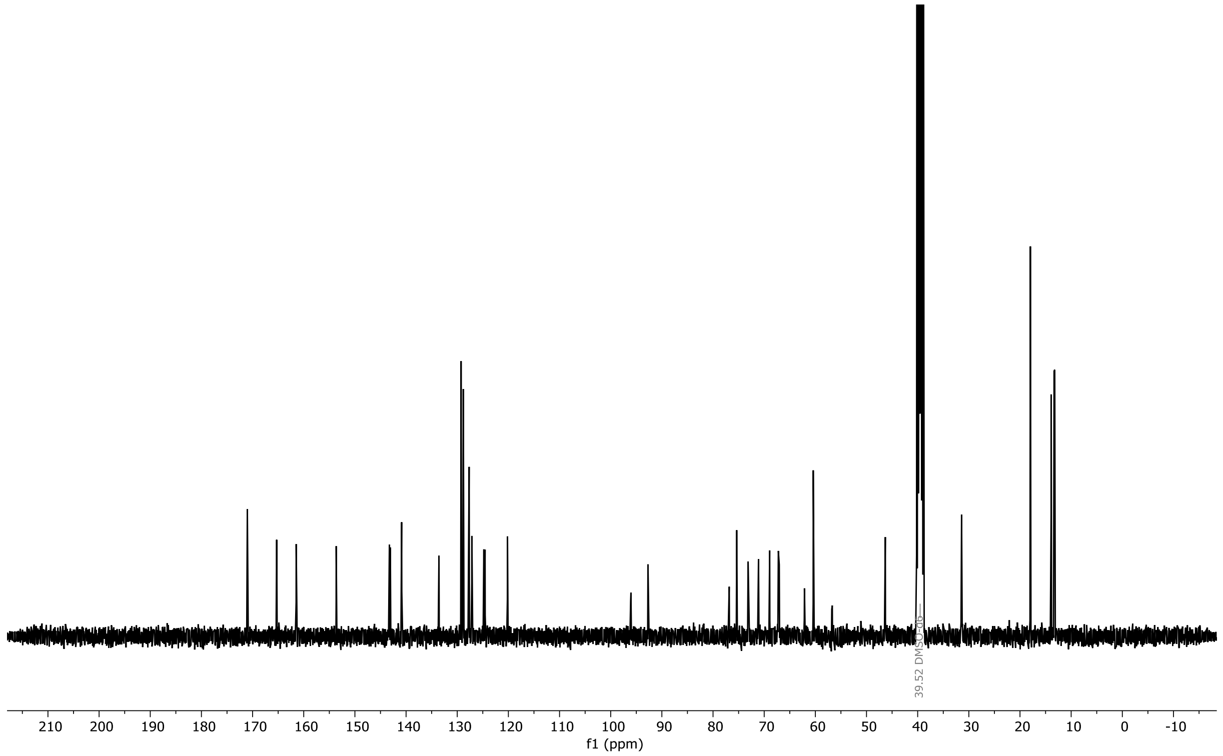

HSQC (DMSO)

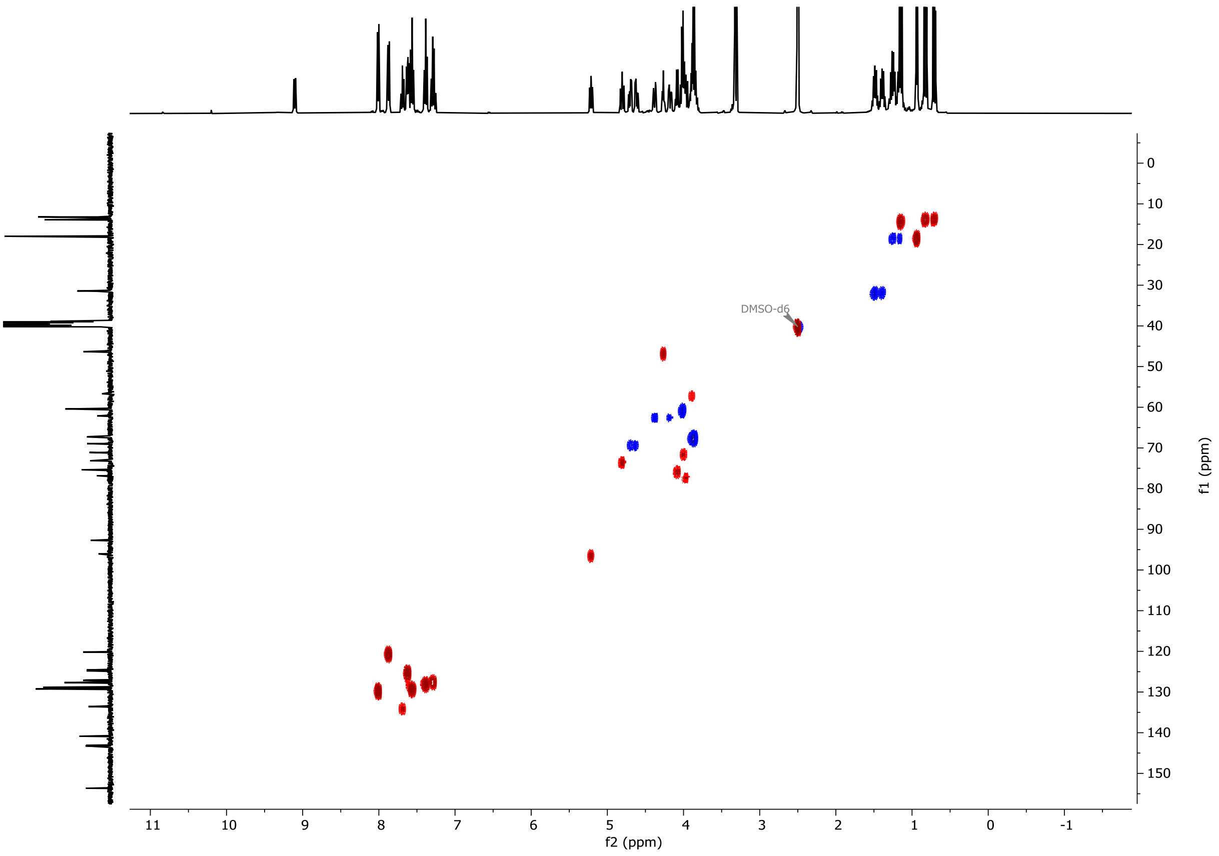

Benzyl 4-O-fluorenylmethoxycarbonyl-3,6-O-benzyl-2-deoxy-2-trichloroacetamido-β-D-glucopyranoside ***5.7***

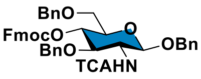

5.7

Phosphate donor **5.6**[4] (700 mg, 0.76 mmol, 1.00 equiv.) and benzyl alcohol (0.16 mL, 1.52 mmol, 2.00 equiv.) were added to a round-bottom flask containing activated 4Å molecular sieves and anhydrous DCM (10 mL) and left to stir for 1.5 h. The mixture was cooled down to -75 °C, and TMSOTf (10.0 µL, 0.583 mmol, 0.70 equiv.) was added and left it to stir for 2h wwhile the remperature was slowly increased to -30 ºC. Then, the reaction was quenched with $NaHCO_3$ (5 mL). The organic layer was washed with NaCl (30 mL), water (30 mL), dried over Na_2SO_4, and the solvent was removed under reduced pressure. The crude product was purified by flash column chromatography using a mixture of hexane/ethyl acetate (3:1) as eluent. The product was obtained as a white solid in 88% yield (551 mg, 0.67 mmol). R_f (Hex/EA 2:1): 0.87; ^{1}H NMR (400 MHz, $CDCl_3$): δ 7.77 – 7.72 (m, 2H), 7.60 – 7.49 (m, 2H), 7.40 – 7.27 (m, 13H), 7.24 – 7.16 (m, 6H), 6.91 (d, *J* = 7.5 Hz, 1H), 5.02 (d, *J* = 8.0 Hz, 1H), 4.98 – 4.86 (m, 2H), 4.62 (d, *J* = 4.5 Hz, 3H), 4.56 (d, *J* = 2.8 Hz, 2H), 4.30 (tt, *J* = 10.4, 5.2 Hz, 3H), 4.11 (td, *J* = 7.2, 1.4 Hz, 1H), 3.77 – 3.71 (m, 1H), 3.71 – 3.64 (m, 2H), 3.59 (dt, *J* = 10.2, 7.8 Hz, 1H) ppm; ^{13}C NMR (101 MHz, $CDCl_3$): δ 162.0, 154.4, 143.4, 143.2, 141.4, 141.4, 138.0, 137.4, 136.8, 128.7, 128.6, 128.6, 128.5, 128.3, 128.3, 128.1, 128.0, 128.0, 127.8, 127.8, 127.3, 127.3, 127.1, 125.2, 125.1, 120.2, 120.2, 98.0, 92.4, 76.2, 74.7, 73.8, 73.2, 71.4, 70.3, 69.5, 60.6, 58.6, 46.8 ppm; ESI-LRMS: m/z $[M-H]^-$ calcd. for $C_{44}H_{40}Cl_3NO_8$ 814.17 found 814.2.

⁕ Product was used without further characterization for the next reaction.

^{1}H NMR ($CDCl_3$)

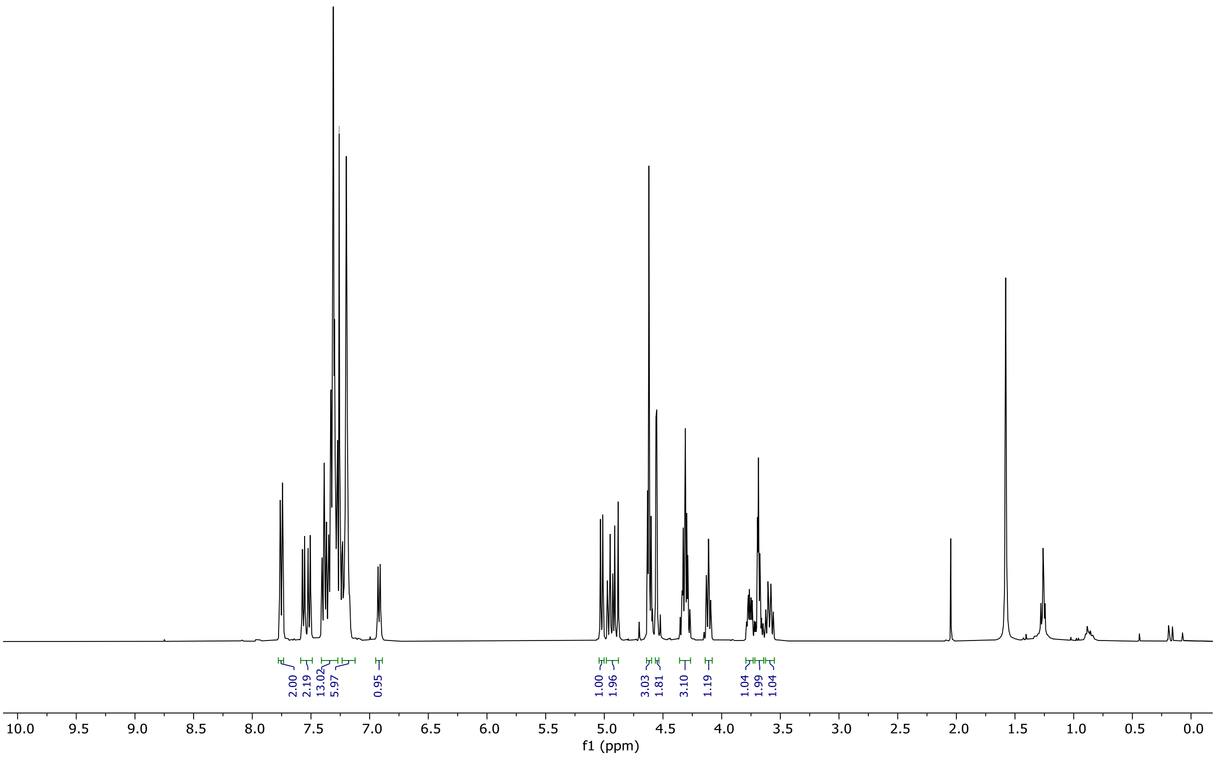

^{13}C NMR ($CDCl_3$)

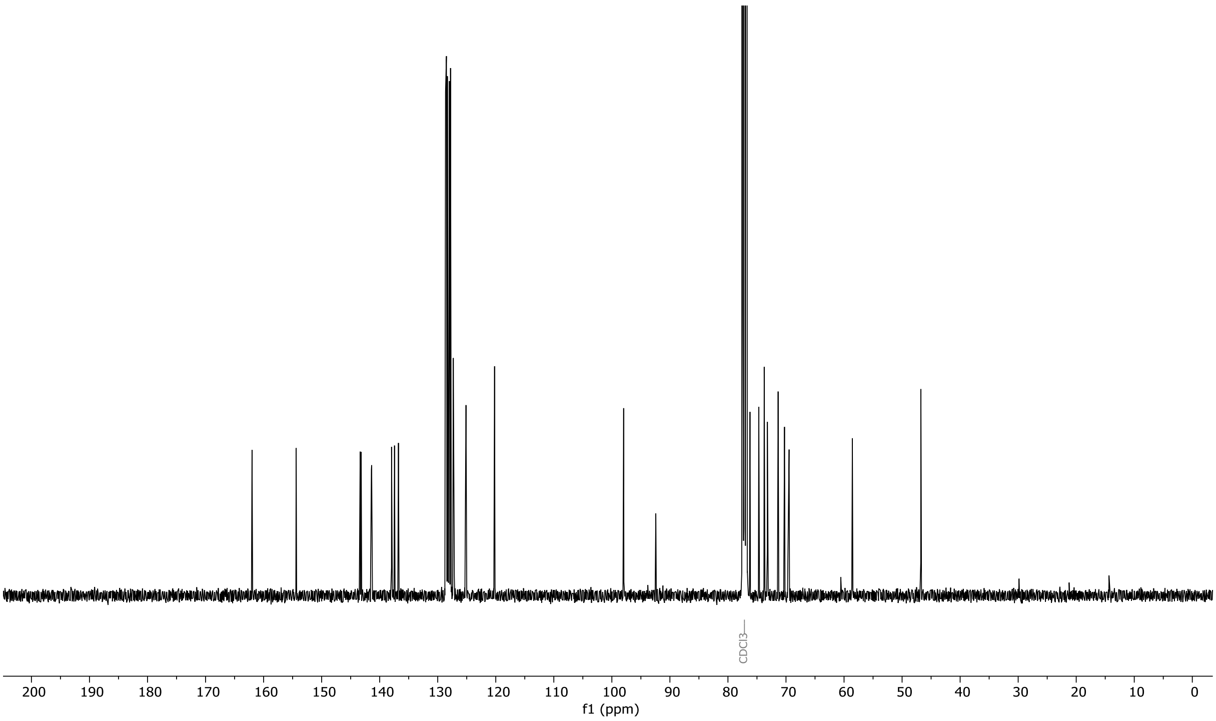

HSQC NMR ($CDCl_3$)

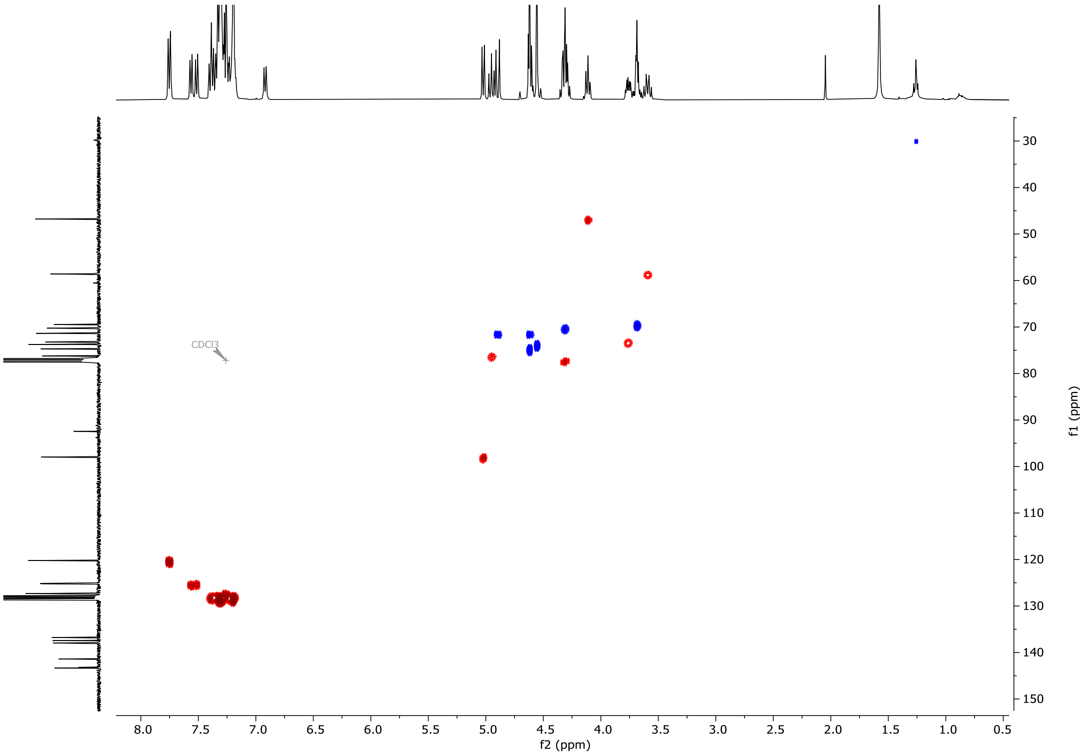

Benzyl 3,6-di-O-benzyl-2-deoxy-2-trichloroacetamido-β-glucopyranoside ***5.8***

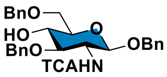

5.8

5.7 (721 mg, 0.88 mmol, 1.00 equiv.) was dissolved in anhydr. DCM (10 mL) under argon atmosphere and the solution was cooled to 0°C. Et_3N (2.45 mL, 17.7 mmol, 20.0 equiv.) was added dropwise and the mixture was left to react 3h at rt. After completion, the mixture was washed with hydrochloric acid (1 M, 40 mL) and NaCl (40 mL). The organic layer was dried over Na_2SO_4 and the solvent was removed under reduced pressure. The residue was purified by flash column chromatography using a mixture of hexane/ethyl acetate (2:1) as eluent. The product was obtained as a white foam in 89% yield (468 mg, 0.78 mmol). R_f (Hex/EA 2:1): 0.42; ^{1}H NMR (400 MHz, $CDCl_3$): δ 7.40 – 7.27 (m, 15H), 6.83 (d, J = 7.7 Hz, 1H), 4.91 (d, J = 8.3 Hz, 1H), 4.87 (d, J = 11.8 Hz, 1H), 4.80 – 4.72 (m, 2H), 4.65 (d, J = 12.0 Hz, 1H), 4.61 – 4.56 (m, 2H), 3.98 (dd, J = 10.4, 8.5 Hz, 1H), 3.84 – 3.71 (m, 3H), 3.60 – 3.50 (m, 2H), 1.58 (s, 1H) ppm; ^{13}C NMR (101 MHz, $CDCl_3$): δ 161.9, 138.1, 137.7, 136.9, 128.8, 128.7, 128.6, 128.3, 128.2, 128.1, 128.0, 98.3, 92.6, 79.7, 74.8, 73.9, 73.7, 73.7, 71.1, 70.6, 58.4 ppm; IR (neat) νmax: 2981, 2253, 1439, 1416, 1375, 1039, 749 cm^{-1}; ESI-HRMS: m/z [M-H]$^-$ calcd. for $C_{29}H_{29}Cl_3NO_6$ 592.1027 found 592.1035.

^{1}H NMR ($CDCl_3$)

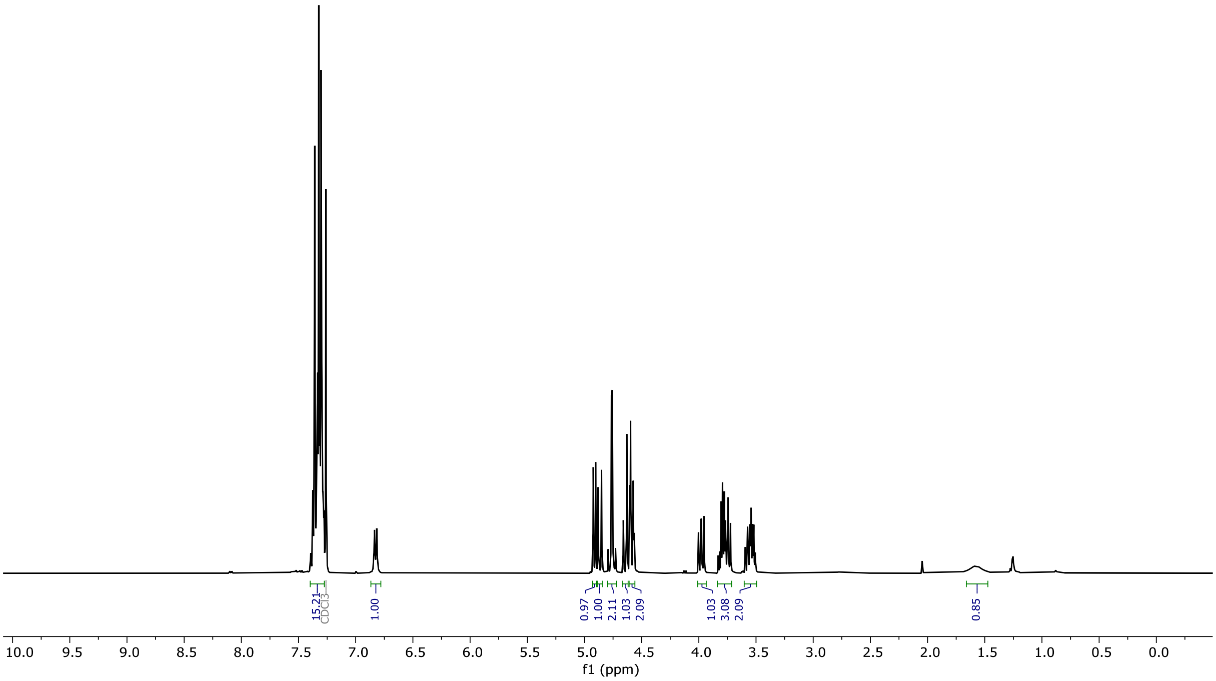

^{13}C NMR ($CDCl_3$)

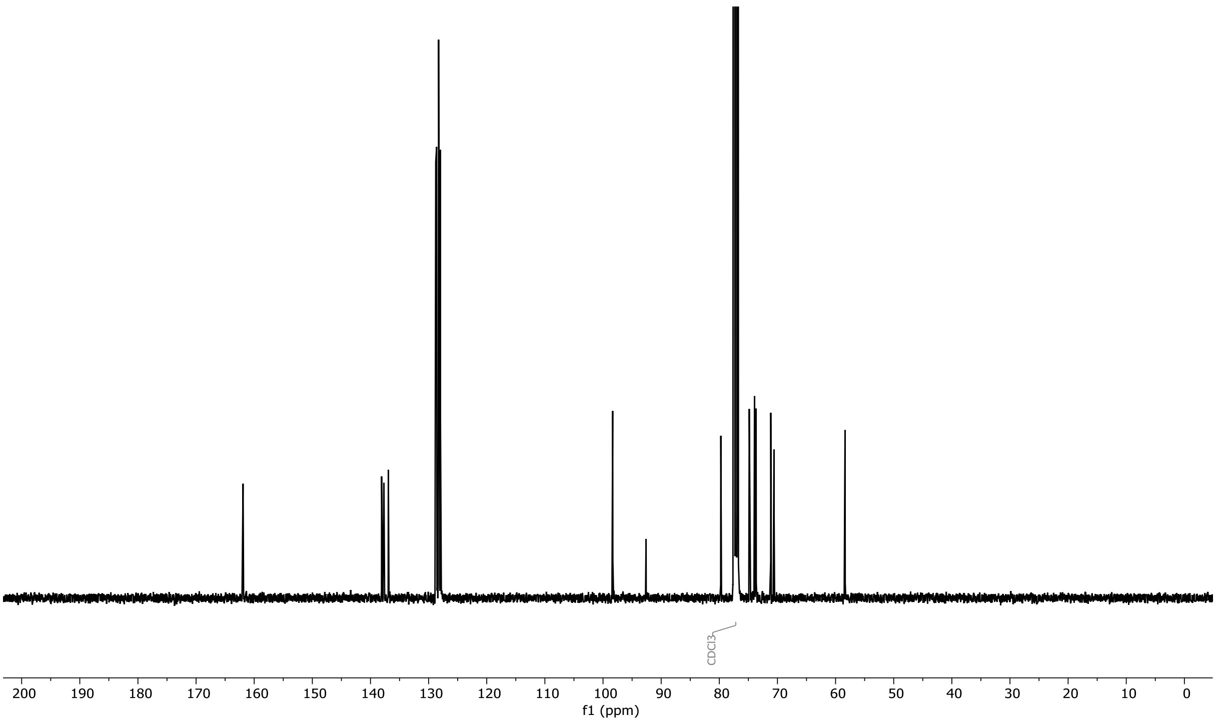

^{13}C HSQC ($CDCl_3$)

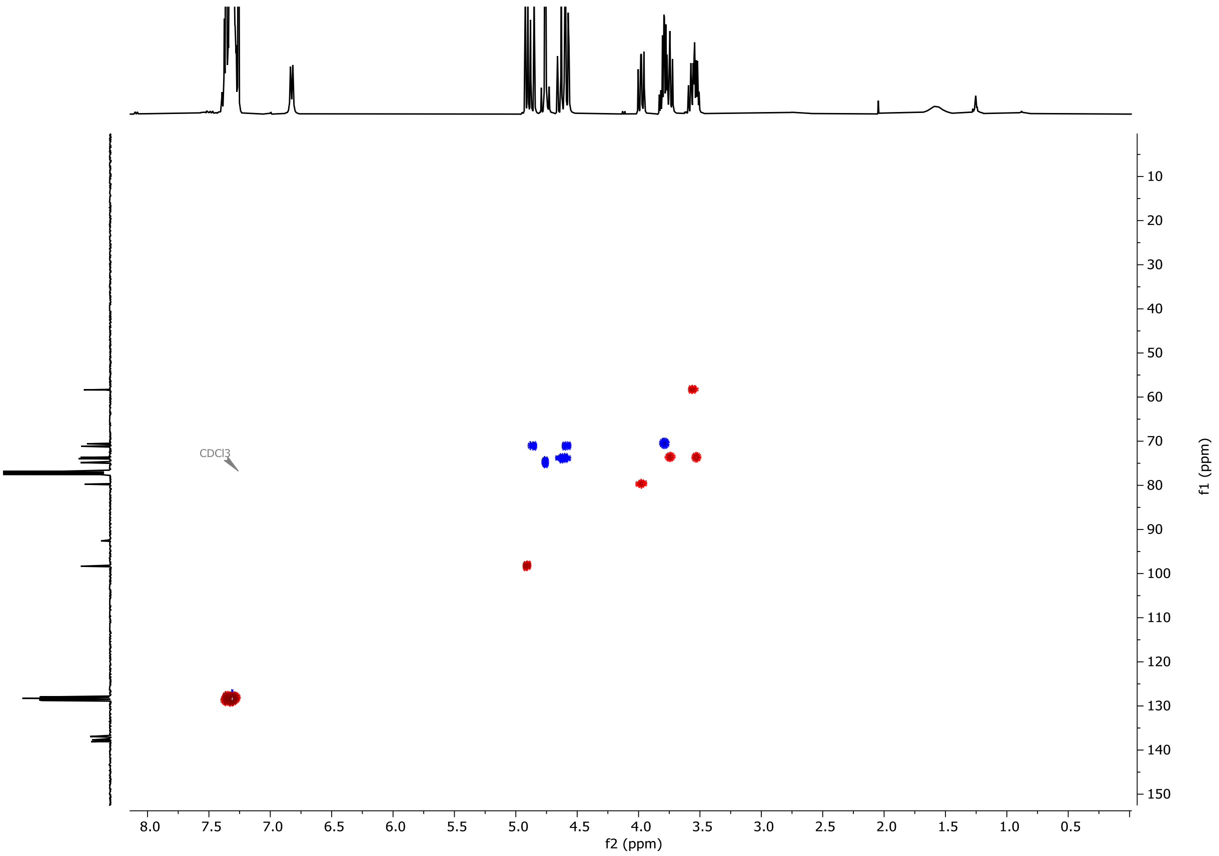

Benzyl 2-deoxy-3-O-[(R)-1`-ethoxycarbonylethyl]-4-O-fluorenylmethoxycarbonyl-6-O-benzoyl-2-trichloroacetamido-β-D-glucopyranosyl-(1→4)-2-deoxy-3,6-di-O-benzyl-2-trichloroacetamido-β-D-gluco-pyranoside **5.9**

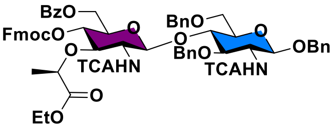

5.8 (162 mg, 0.29 mmol, 1.00 equiv.) and **5.5** (306 mg, 0.32 mmol, 1.10 equiv.) were dissolved in anhydrous DCM (6 mL), 4Å molecular sieves were added under argon atmosphere, and the suspension was stirred 1h at rt. The mixture was cooled down to -75°C, and TMSOTf (30 µL, 0.16 mmol) dissolved in anhydrous DCM (0.5 mL) was added dropwise and the temperature was left slowly to increase to -30°C within 2h. Additional 0.5mL of activator solution was added and the temperature was increased to 0°C within 2 h. The solution was carefully quenched with $NaHCO_3$ solution (5 mL). The organic layer was separated, washed with NaCl (2×10 mL), DCM and dried over anhydrous Na_2SO_4. The solvent was removed under reduced pressure and the crude product was purified by flash column chromatography using a mixture of hexane/DCM/ethyl acetate (6:2:1). The desired product was obtained as a yellowish foam in 71% (278 mg, 0.21 mmol). R_f (Hex/DCM/EA 4:2:1): 0.42; ^{1}H NMR (400 MHz, $CDCl_3$): δ 8.47 (d, *J* = 6.1 Hz, 1H), 8.03 – 7.98 (m, 2H), 7.77 (d, *J* = 7.6 Hz, 2H), 7.59 (d, *J* = 7.6 Hz, 1H), 7.55 – 7.48 (m, 2H), 7.40 (q, *J* = 7.8 Hz, 4H), 7.36 – 7.26 (m, 12H), 7.26 – 7.23 (m, 2H), 7.18 (dq, *J* = 5.6, 3.4 Hz, 4H), 4.95 – 4.84 (m, 2H), 4.84 – 4.70 (m, 3H), 4.60 (dd, *J* = 27.9, 11.9 Hz, 2H), 4.53 – 4.40 (m, 3H), 4.37 – 4.17 (m, 7H), 4.16 – 4.01 (m, 2H), 3.95 (q, *J* = 7.3 Hz, 1H), 3.88 – 3.74 (m, 5H), 3.66 (q, *J* = 4.9 Hz, 1H), 3.49 (ddd, *J* = 9.8, 4.8, 3.3 Hz, 1H), 1.35 (t, *J* = 7.2 Hz, 3H), 1.25 (dd, *J* = 7.1, 2.7 Hz, 3H) ppm; ^{13}C NMR (100 MHz, $CDCl_3$): δ 174.0, 166.0, 163.1, 161.8, 153.9, 143.4, 142.6, 141.4, 141.3, 138.2, 138.1, 137.3, 133.3, 129.7, 129.6, 128.5, 128.4, 128.2, 127.9, 127.7, 125.0, 124.7, 120.2, 120.2, 100.9, 99.3, 92.9, 92.6, 78.3, 76.1, 75.8, 75.2, 74.9, 73.5, 73.4, 71.6, 71.0, 70.5, 69.0, 62.7, 61.9, 56.7, 56.0, 46.6, 18.7, 18.7, 14.2 ppm; IR (neat)

νmax: 2981, 2350, 2253, 1444, 1412, 1375, 1039, 749 cm^{-1}; ESI-HRMS: m/z $[M-H]^-$ calcd. for $C_{64}H_{61}Cl_6N_2O_{16}$ 1323.2127 found 1323.1946.

^1H NMR ($CDCl_3$)

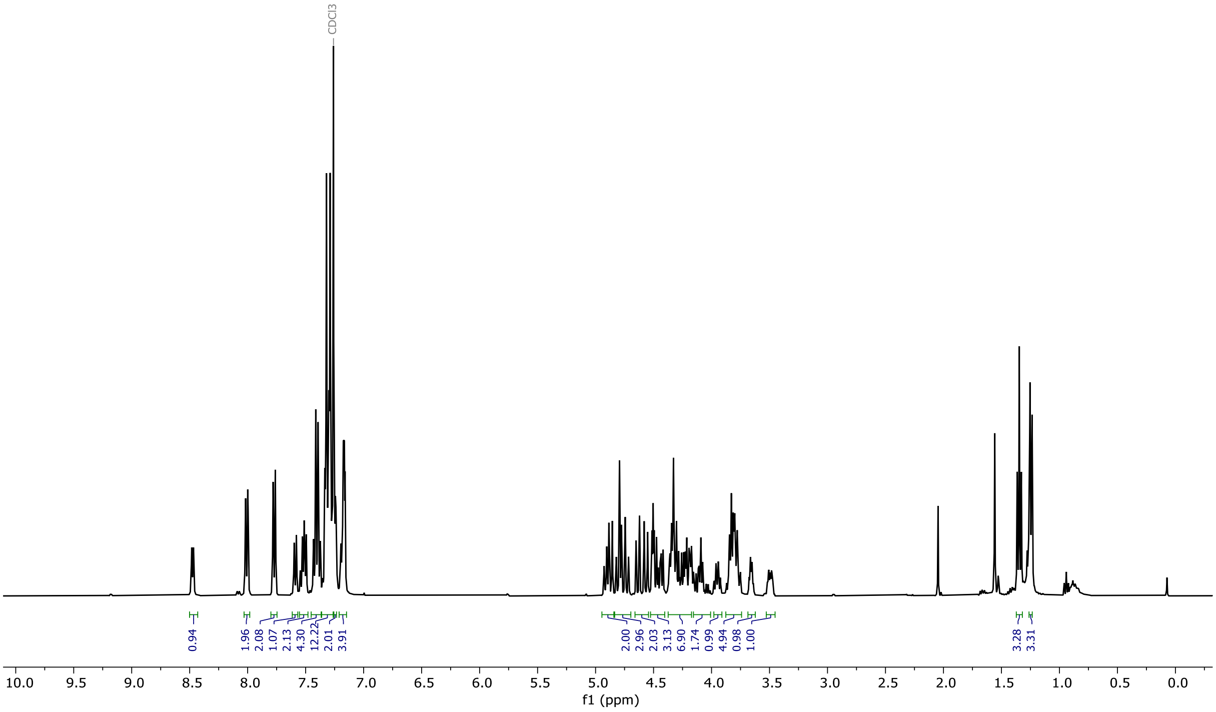

^{13}C NMR ($CDCl_3$)

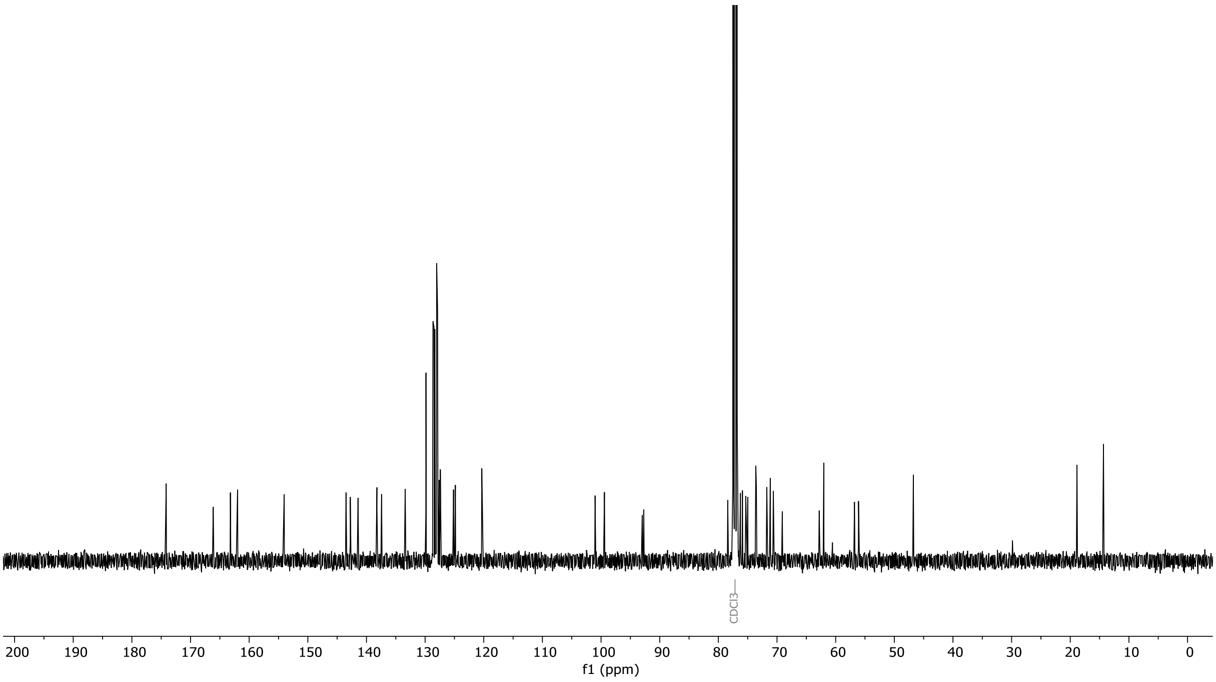

HSQC NMR ($CDCl_3$)

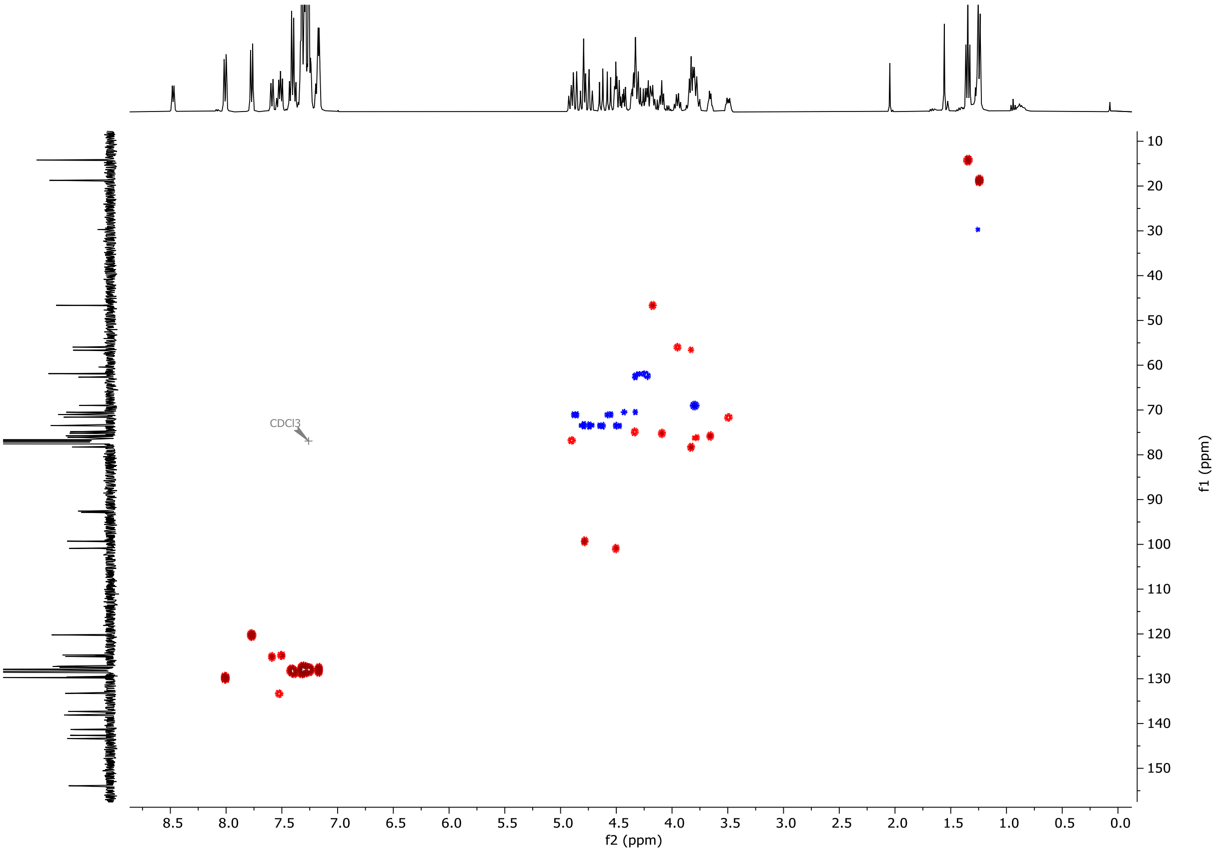

2-acetamido-3-O-[(R)-1`-carboxyethyl]-2-deoxy-β-D-glucopyranosyl-(1 → 4)-2-acetamido-2-deoxy-β-D-glucopyranoside **5**

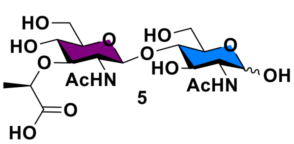

To a solution of **5.9** (270 mg, 0.20 mmol, 1.00 equiv.) in THF/H_2O (4:1, 5 mL), was added LiOH (7.30 mg, 0.31 mmol, 1.50 equiv.) and the mixture was stirred for 6 h. Then, the solution was neutralized Amberlite, filtered, and the solvent was removed under reduced pressure. The crude was then redissolved in H2O/AcOH/THF (1:1:2, 4 mL) and 200 mg of Pd(C) were added. The resulting mixture was stirred under hydrogen atmosphere for 5 days. The catalyst was filted off and the solvent was removed under reduced pressure. The crude product was purified using preparative reverse phase HPLC (Hypercarb column, 150 x 10 mm, 5 µm), flow rate of 3 mL/min with 5% ACN in H_2O (0.1% formic acid) as eluents [isocratic (5 min), linear gradient to 20% ACN (30 min), linear gradient to 100% ACN (5 min)]. The pure compound was analyzed using an analytical HPLC (Hypercarb column, 150 x 4.6 mm, 3 µm) flow rate of 0.7 mL/min with 5% ACN in H_2O (0.1% formic acid) as eluents [isocratic (5 min), linear gradient to 20% ACN (30 min), linear gradient to 100% ACN (5 min)]. The desired product was obtained after lyophilization as a an off white solid in 15% yield over 2 steps (15 mg, 0.03 mmol). ^{1}H NMR (400 MHz, D_2O) δ 5.06 (d, J = 2.7 Hz, 1H), 4.57 (d, J = 7.8 Hz, 1H), 4.45 `(dd, J = 8.4, 3.8 Hz, 1H), 4.17 (q, J = 6.8 Hz, 1H), 3.81 – 3.69 (m, 3H), 3.68 – 3.56 (m, 3H), 3.54 – 3.36 (m, 5H), 1.92 (d, J = 4.5 Hz, 6H), 1.23 (d, J = 6.9 Hz, 3H); ^{13}C NMR (101 MHz, D_2O) δ 174.5, 174.4, 101.7, 94.8, 90.3, 80.9, 79.7, 75.6, 74.5, 72.4, 69.9, 69.2, 69.1, 60.4, 59.9, 56.0, 54.4, 53.6, 22.2, 18.7 ppm; IR (neat) νmax: 3313, 1638 cm^{-1}; ESI-HRMS: m/z $[M-H]^-$ calcd. for $C_{19}H_{31}N_2O_{13}$ 495.1827 found 497.1985.

^{1}H NMR (D_2O)

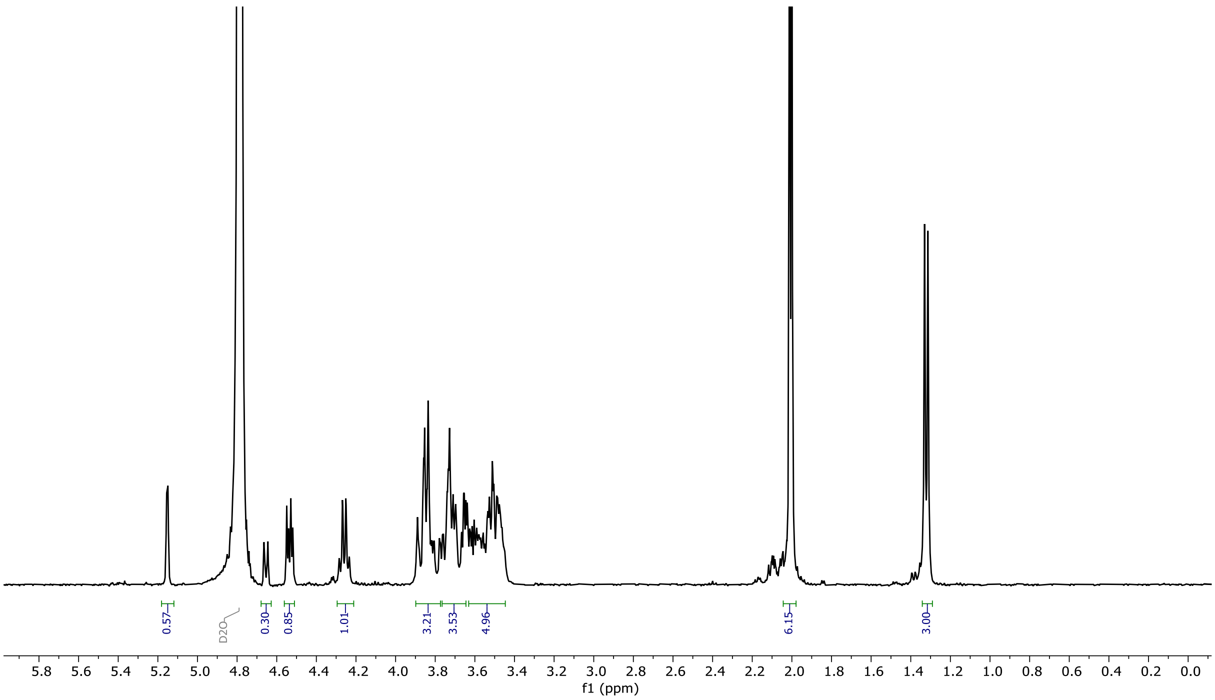

^{13}C NMR (D_2O)

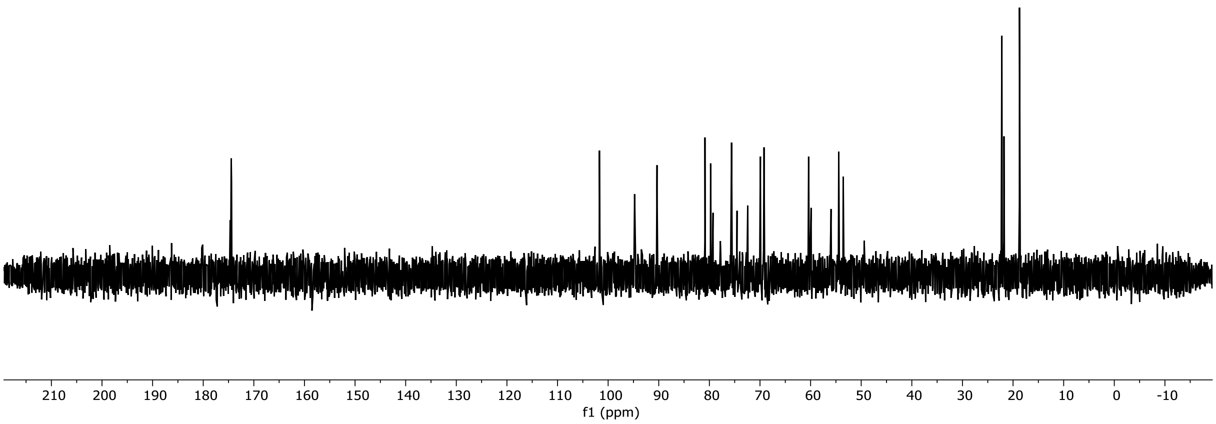

HSQC (D_2O)

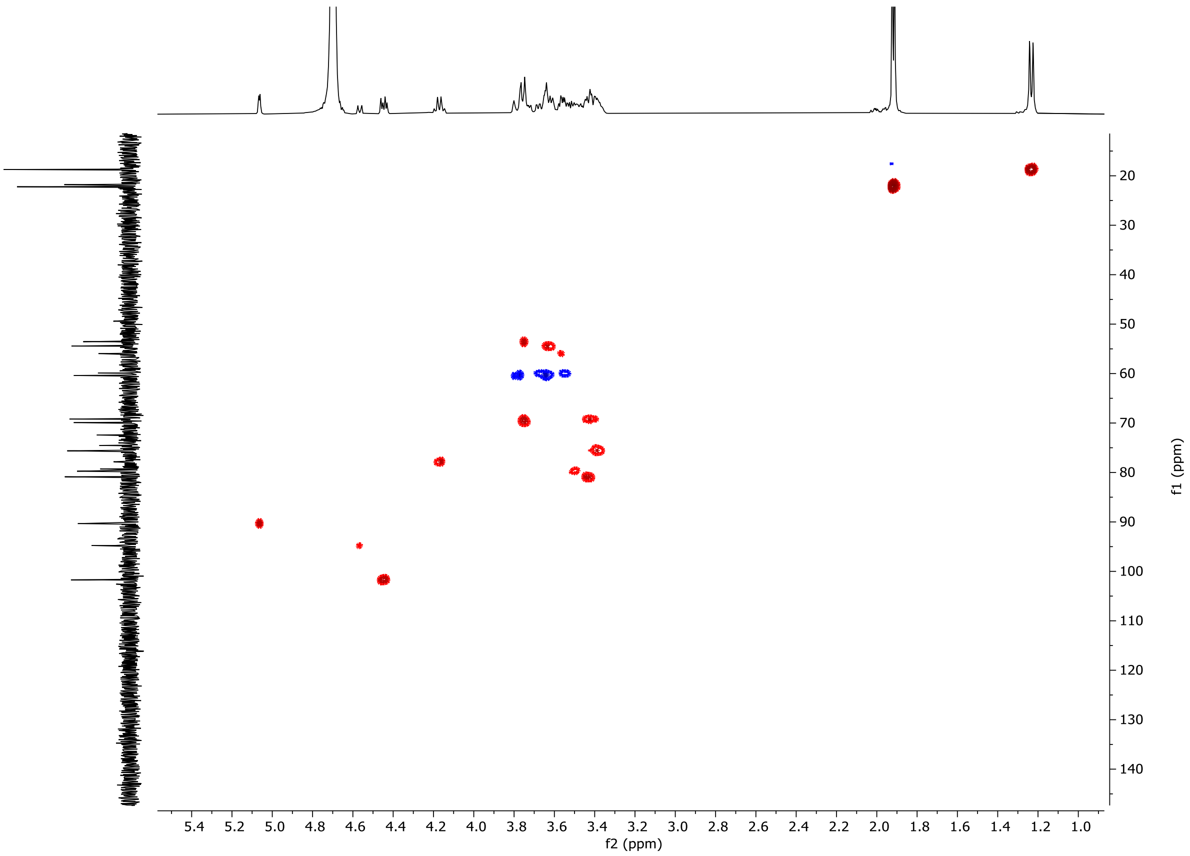

RP-HPLC (ELSD trace, method B, t_R = 23.5 min, t_R = 26.6 min of anomers)

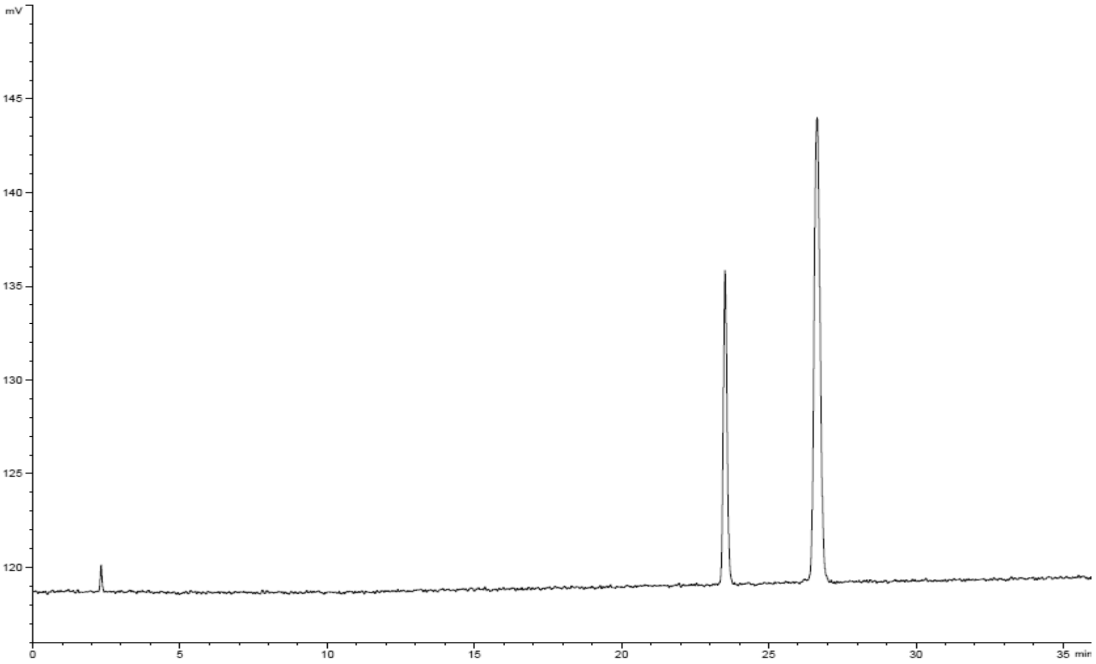

G. Modules of synthesis

Module A: Acceptor slide preparation for synthesis (436 min)

All syntheses were performed on amino functionalized glass slides. The slides were functionalized as described in *Section D*. The slides, prior to the synthesis, were placed into a petri dish, and swollen for 30 min in DMF on a shaker at rt (300 rpm). Then, each slide was washed with DMF (3×3 min), deprotected using Fmoc-deprotection solution, and washed with DMF (3×3 min), MeOH (1×2 min), and DCM (1×3 min).

Module B: Donor slide preparation

Donor slides bearing 10% or 20% amino acid in SLEC (see *Preparation of Solutions*) were spin coated onto the polyimide coated microscope glasses (80 rps) using a positive displacement pipette.

Module C: Laser transfer of AA(s)

For the array synthesis, a spot pitch of 150 μm was used. A laser scanning system with 405 nm wavelength and 210 mW maximum output power was used (~50 μm laser focus diameter). For the automated transfer, the donor and acceptor slides were placed in the slide holder and a robot handled them with 20 μm precision. For the applied lasing parameters see *Section K*, while a laser gradient from 50 to 120 mW was applied with a pulse duration range of 2-12 ms for the required optimization of the laser parameters for the used amino acids.

Module D: Coupling process (36 min)

The coupling reaction was accomplished under heat in an oven under nitrogen atmosphere at 95°C for 10 min. Subsequently, the slides were washed with acetone twice. Initially for 2 min in an ultrasonic bath and then for another 2 min in a petri dish on a shaker (450 rps). Then, slides were dried in a jet of air. The cLIFT transfer of the same amino acid pattern, the coupling, and the acetone wash steps were repeated twice. Each time a new donor slide was used for every transfer and coupling cycle.

Module E: Capping (74 min)

The remaining unreacted amino groups on the acceptor slides after coupling were subjected for acetylation for 2 min and then for 30 min. The same process was repeated with a freshly prepared capping solution for another 30 min in rt (300 rpm). The slides were washed with DMF (3×3 min), MeOH (1×2 min), DCM (1×1 min), and dried in a jet of air.

All modules were repeated for the synthesis of the desired peptide sequences.

Post-Synthesis manipulation

Fmoc-deprotection and washing of the respective arrays performed as reported previously.

Module F1: Attachment of MDP 1

*MurNAc-*D*-Ala-*L*-iGln* (2.50 mg, 5.00 µmol, 1.00 equiv.) was dissolved in 200 μL of anhydr. DMF in a vial. DIC (1.56 µL, 10.0 µmol, 2.00 equiv.) and PfpOH (1.84 mg, 10.0 µmol, 2.00 equiv.), were added consecutively and the vial was shaken for a few seconds. The resulting solution was pipetted on the amino glass slide and another slide was placed on top (sandwich functionalization method). The slides were left to react overnight in a petri dish.

Then, the slides were washed consecutively with DMF (3×3 min), MeOH (1×2 min), and DCM (1×1 min), and dried by a jet of air.

Module F2: Attachment of MDP 2

*MurNAc-L-Ala-L-*iGln (2.50 mg, 5.00 µmol, 1.00 equiv.) was dissolved in 200 µL of anhydr. DMF in a vial. DIC (1.56 µL, 10.0 µmol, 2.00 equiv.) and PfpOH (1.84 mg, 10.0 µmol, 2.00 equiv.), were added consecutively and the vial was shaken for a few seconds. The resulting solution was pipetted on the amino glass slide and another slide was placed on top (sandwich functionalization method). The slides were left to react overnight in a petri dish. Then, the slides were washed consecutively with DMF (3×3 min), MeOH (1×2 min), and DCM (1×1 min), and dried by a jet of air.

Module F3: Attachment of 2-acetamido-3-O-[(R)-1`-carboxyethyl]-2-deoxy-β-D-glucopyranoside (MurNAc) 3

MurNAc **3** (3.00 mg, 10.0 µmol, 1.00 equiv.), was dissolved in 200 µL of anhydr. DMF in a vial. DIC (3.12 µL, 20.0 µmol, 2.00 equiv.) and PfpOH (3.68 mg, 20.0 µmol, 2.00 equiv.), were added consecutively and the vial was shaken for a few seconds. The resulting solution was pipetted on the amino glass slide and another slide was placed on top (sandwich functionalization method). The slides were left to react overnight in a petri dish. Then, the slides were washed consecutively with DMF (3×3 min), MeOH (1×2 min), and DCM (1×1 min), and dried by a jet of air.

Module F4: Attachment of 2-acetamido-2-deoxy-β-D-glucopyranosyl-(1 → 4)-2-acetamido-3-O-[(R)-1`-carboxyethyl]-2-deoxy-β-D-glucopyranoside [GlcNAc (1→4) MurNAc] 4

Dimer **4** (2.50 mg, 5.00 µmol, 1.00 equiv.) was dissolved in 200 µL of anhydr. DMF in a vial. DIC (1.56 µL, 10.0 µmol, 2.00 equiv.) and PfpOH (1.84 mg, 10.0 µmol, 2.00 equiv.), were added consecutively and the vial was shaken for a few seconds. The resulting solution was pipetted on the amino glass slide and another slide was placed on top (sandwich functionalization method). The slides were left to react overnight in a petri dish. Then, the slides were washed consecutively with DMF (3×3 min), MeOH (1×2 min), and DCM (1×1 min), and dried by a jet of air.

Module F5: Attachment of 2-acetamido-3-O-[(R)-1`-carboxyethyl]-2-deoxy-β-D-glucopyranosyl-(1 →4)-2-acetamido-2-deoxy-β-D-glucopyranoside [MurNAc (1→4) GlcNAc] 5

Dimer **5** (2.50 mg, 5.00 µmol, 1.00 equiv.) was dissolved in 200 µL of anhydr. DMF in a vial. DIC (1.56 µL, 10.0 µmol, 2.00 equiv.) and PfpOH (1.84 mg, 10.0 µmol, 2.00 equiv.), were added consecutively and the vial was shaken for a few seconds. The resulting solution was pipetted on the amino glass slide and another slide was placed on top (sandwich functionalization method). The slides were left to react overnight in a petri dish. Then, the slides were washed consecutively with DMF (3×3 min), MeOH (1×2 min), and DCM (1×1 min), and dried by a jet of air.

***Module F6: Attachment of 2-Azidoethyl 2-acetamido-2-deoxy-β-D-glucopyranoside (GlcNAc-N_3)* 6**

The CuAAC reaction between the sugar azide and the *PEGMA/MMA β-Ala-X-β-Ala-Pra-NHAc* glass solid support was performed as reported in the literature.[5]

Module G: Deprotection of acid labile protecting groups (132 min)

Deprotection of the acid labile protecting groups of the amino acids was performed by immersing the acceptor in the TFA-deprotection solution (see *Preparation of Solutions*) (3×30 min). Next, the acceptor slides were washed with DCM (1×5 min) and DMF (1×5 min), neutralized using a 5% DIPEA in DMF (v/v) solution for 20 min. Then, the slides were washed consecutively with DMF (3×3 min), MeOH (1×2 min), and DCM (1×1 min), and dried by a jet of air.

H. Synthesis & validation of PGN arrays by mAbs on different surface functionalizations

Functionalization of acceptor glass slides: PEGMA/MMA-β-Ala-X-β-Ala- NHFmoc

The pre-functionalization was performed as reported in *Section E*. However, two different amino acids, Fmoc-Asp(OtBu)-OPfp, Fmoc-Ala-OPfp, and two spacers Fmoc-8-Aoc-OH and Fmoc-8-O_2Oc-OH, marked as X, were introduced prior to the attachment of Fmoc−β−Ala-OH in solution, to investigate the effect of the acceptor functionalization on the antibody recognition.

Preparation of donor slides

Donor slides bearing the Fmoc-Asp(OtBu)-OPfp and Fmoc-Ala-OPfp were prepared as reported in previous sections.

For the non-pre-activated spacers, the activation was performed *in-situ* during the preparation of the spin-coating solution and was used without isolation or further characterization. The non-pre-activated spacer (5.00 mg), DIC (2.50 µL), and PfpOH (2.50 mg) were dissolved in 50 µL anhydr. DMF, while SLEC (25.0 mg) was dissolved in 450 µL anhydr. DCM. The first solution was added into the second matrix solution. The final mixture was shaken for 2 min (vibrating orbital shaker) and afterwards the solution was spin-coated on top of the polyimide.

Screening of Monoclonal Antibodies

Following the already established protocols, six different arrays were generated:

i. *PEGMA/MMA-β-Ala-X-β-Ala- NHAc*
ii. *PEGMA/MMA-β-Ala-X-β-Ala- MDP **1***
   *PEGMA/MMA-β-Ala-X-β-Ala- MDP **2***
iii. *PEGMA/MMA-β-Ala-X-β-Ala- MurNAc **3***
iv. *PEGMA/MMA-β-Ala-X-β-Ala- Dimer 4*
   *PEGMA/MMA-β-Ala-X-β-Ala- Dimer **5***
v. *PEGMA/MMA β-Ala-X-β-Ala-Pra (GlcNAc **6**)-NHAc*

Freshly synthesized arrays were incubated with the respective mAbs before and after treatment with the TFA deprotection solution. In both cases, the synthesized arrays were incubated with the chosen monoclonal antibodies against peptidoglycan (PGN) fused with either mouse or human-Fc antibody fragment (mAbs). The results were detected after incubation of the respective array with secondary goat polyclonal anti-mouse or anti-human IgG fused with a fluorophore as shown in **Table S1**.

Characteristics of mAbs

According to the literature, mAb 2E9 demonstrates specificity towards muramyl dipeptide (MDP) and lysozyme-solubilized cell walls of various Gram-positive bacteria.[6] Isothermal titration calorimetry (ITC) analysis showed that 2E7 binds more strongly to MDP **1** than to the isomeric biologically inactive MDP **2**structure. Furthermore, indirect competitive enzyme-linked immunosorbent assay in the presence of MDP revealed that 2E7 does not bind common moieties of PGN such as GlcNAc, MurNAc, Mur; L-Ala; and D-iGln but it binds weakly to MurNAc-L-Ala.[7] The F598 mAb recognizes poly-*N*-acetylglucosamine (PNAG) structures found in a wide range of microbes, including both Gram-negative and Gram-positive bacteria, as well as pathogens, fungi, and protozoan parasites, and it is extensively used for the identification of *Staphylococcus aureus* biofilms.[8,9] Lastly, commercial MAB995 (clone 3F6B3) is specific towards insoluble 3D polymer complex PGNs from *Streptococcus mutans*, enabling detailed immunohistochemical and ELISA analyses.[10]

Table S1: Implemented peptidoglycan/control mAbs with their corresponding secondary fluorescently labelled antibodies.

ID	Origin	Class	Isotype & subclass	Conc. [µg/mL]	Dilution	Secondary antibody staining
**2E9**	Rotterdam[5]	mouse	IgG3	3.2 mg/mL	1:10	*a-mouse IgG Fc CF633 (2 mg/mL, 1:500)*
**2E7**	Singapore[6]	mouse	IgG1	1 mg/mL	1:500	*a-mouse IgG Fc CF633 (2 mg/mL, 1:500)*
**F598**	Harvard[7]	human	IgG1	17 mg/mL	1:250	*a-human Fc DyLight® 650 (0.5 mg/mL, 1:500)*
**MAB995**	Merck	mouse	IgG1	/	1:100	*a-mouse IgG Fc CF633 (2 mg/mL, 1:500)*

Prior to mAb incubation, to avoid unspecific binding, the arrays were incubated with a blocking buffer (Rockland, Limerick-Pennsylvania, USA; blocking buffer for fluorescent western blotting MB-070) for 30 min. Subsequently, the arrays were incubated with mAbs in staining buffer (10% Rockland blocking buffer in PBS-T, pH: 7.4) for 1 h on an orbital shaker. To remove unbound antibody, the arrays were shortly washed with staining buffer (1×1 min). Secondary staining followed, using anti-mouse or anti-human IgG 1:500 concentration for 1h. The arrays were washed with staining buffer (1×1 min), and then were rinsed with Tris buffer (1 mM Tris-HCl buffer, pH: 7.4) to remove all remaining salt residues, and dried by a jet of air.

i. *PEGMA/MMA-β-Ala-X-β-Ala- NHAc*

The arrays *PEGMA/MMA-β-Ala-X-β-Ala- NHAc,* without additional modification served as negative controls for every monoclonal and polyclonal antibody and no binding was detected.

ii. *PEGMA/MMA-β-Ala-X-β-Ala- MDP* ***1***
PEGMA/MMA-β-Ala-X-β-Ala- MDP ***2***

Staining of the arrays bearing MDP **1** and MDP **2** respectively yielded interesting observations (**Figure S2**). The 2E7 antibody demonstrated strong binding on both the MDP **1** and MDP **2** arrays, with the intensities in both

arrays showing the same trend and falling within the same intensity range. This consistent binding pattern was observed regardless of whether the arrays underwent TFA treatment.

In contrast, the 2E9 and MAB995 antibodies exhibited weak affinity towards the MDP **1** arrays, while no binding was detected on the MDP **2** arrays. This indicates that these antibodies have a low affinity for the MDP **1** arrays, but do not recognize the MDP **2** arrays. Additionally, the effect of TFA treatment on the binding profile was notable. While the 2E7 mAb continued to show the same binding trend after TFA treatment, the overall intensities were significantly lower on the MDP **1** array post-treatment. Despite this reduction in intensity, the binding remained consistent with the pre-TFA treatment observations. These findings underscore the specific of the 2E7 mAb to both MDP **1** and MDP **2** arrays, and highlight the differential binding behavior of the 2E9 and MAB995 antibodies, as well as the impact of TFA treatment on antibody binding intensity.

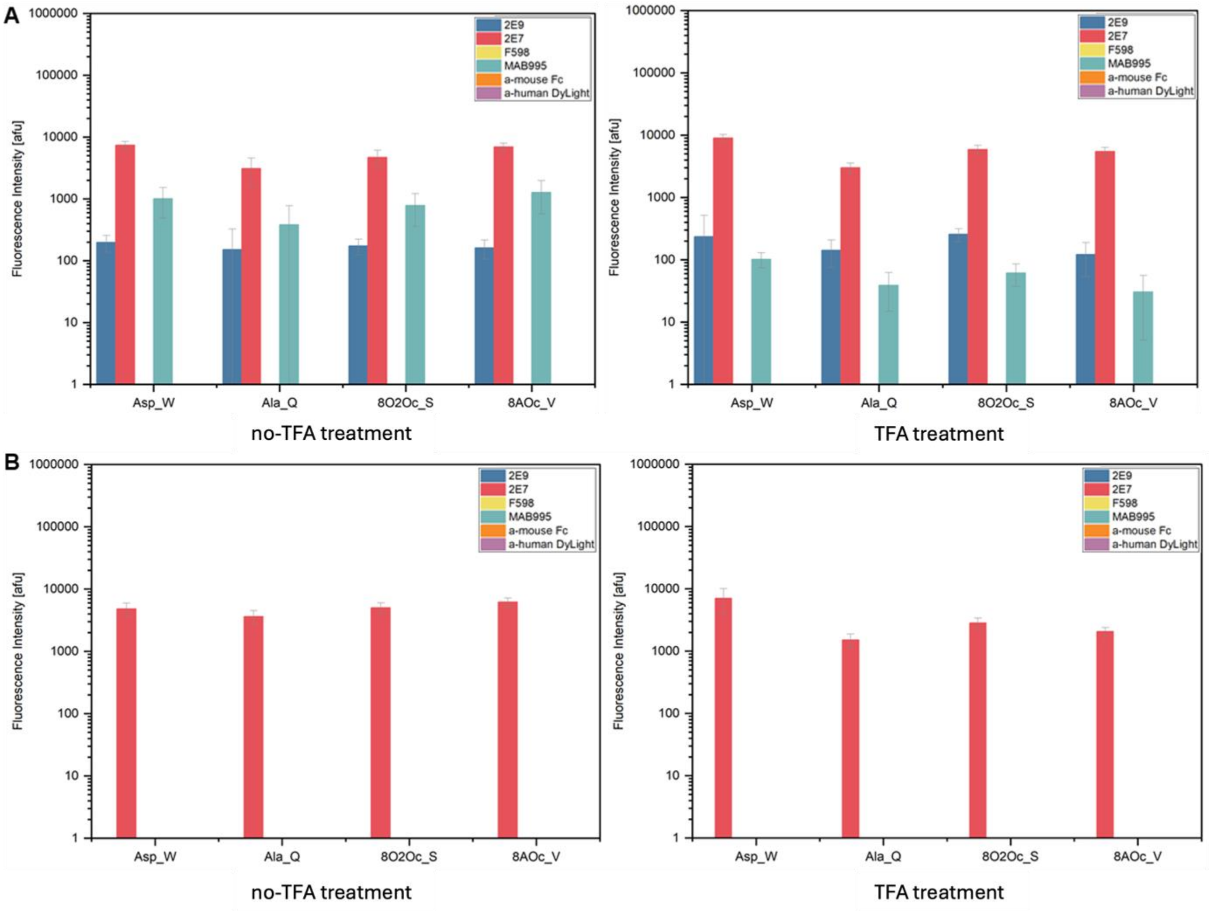

Figure S2. Fluorescence staining intensities of A) PEGMA/MMA-β-Ala-X-β-Ala-MDP **1** functionalized arrays and B) PEGMA/MMA-β-Ala-X-β-Ala-MDP 2 functionalized arrays with three anti-PGN and one anti-PNAG antibodies with and without TFA-treatment. Data shown as median ± standard deviation (SD) as error bars, n = 125.

iii. PEGMA/MMA-β-Ala-X-β-Ala-MurNAc ***3***

The generated arrays with MurNAc **3** as the terminal carbohydrate moiety were incubated with the antibodies listed in **Table S1**. As shown in **Figure S3**, binding to the MurNAc **3** moieties was detected only for 2E7 antibody after secondary fluorescence staining with the corresponding secondary anti-mouse polyclonal antibody. Despite the decreased intensities observed after treatment of the array with the TFA-deprotection solution, the same binding was maintained with and without TFA treatment. It is considered that protonation of the oxygen on the C-

3 position occurs during TFA treatment, leading to hydrolytic cleavage of the sugar residues. In summary, we observed from our studies, that 2E7 shows high selectivity towards terminal MurNAc moieties, while the fluorescence intensity was more pronounced on the structures found in Gram-positive bacteria bearing D-iGln in the position 2 of the stem peptides.

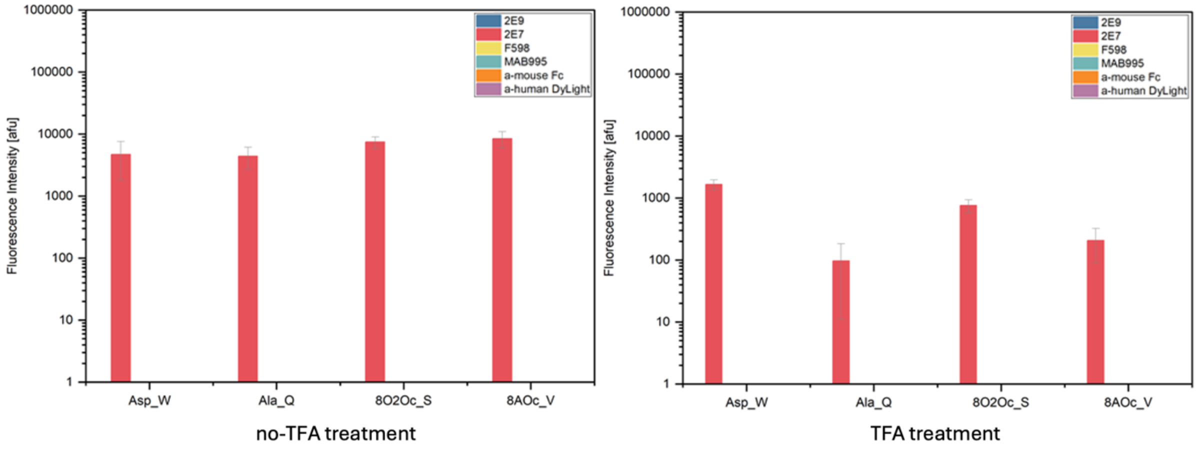

Figure S3: Fluorescence staining intensities of PEGMA/MMA-β-Ala-X-β-Ala-MurNAc **3** functionalized arrays with three anti-PGN and one anti-PNAG antibodies with and without TFA-treatment. Data shown as median ± SD as error bars, n = 125.

v. *PEGMA/MMA-β-Ala-X-β-Ala- Dimer* ***4***

PEGMA/MMA-β-Ala-X-β-Ala- Dimer ***5***

Dimer **4** and **5** (**Figure S4**), displayed distinct differences in binding selectivity and their responses to TFA-treatment. Antibodies 2E9 and MAB995 showed binding on the arrays bearing dimer **4**. The binding intensities remained consistent with slightly lower intensities, regardless of TFA-treatment, indicating a stable and unaffected binding trend after acidic treatment.

In contrast, dimer **5** exhibited a broader binding profile, interacting with all antibodies tested. Without TFA-treatment, 2E9 antibodies showed the highest selectivity, followed by F598 and MAB995 antibodies, while 2E7 antibody exhibited minimal to no signal, suggesting negligible binding or background noise. Following TFA-treatment, the overall fluorescence intensities of dimer **5** notably decreased. Specifically, the binding intensities for 2E9 and MAB995 antibodies decreased following the same binding trend of dimer **4**. In contrast, 2E7 and F598 antibodies continued to exhibit lower binding, comparable to background signal levels.

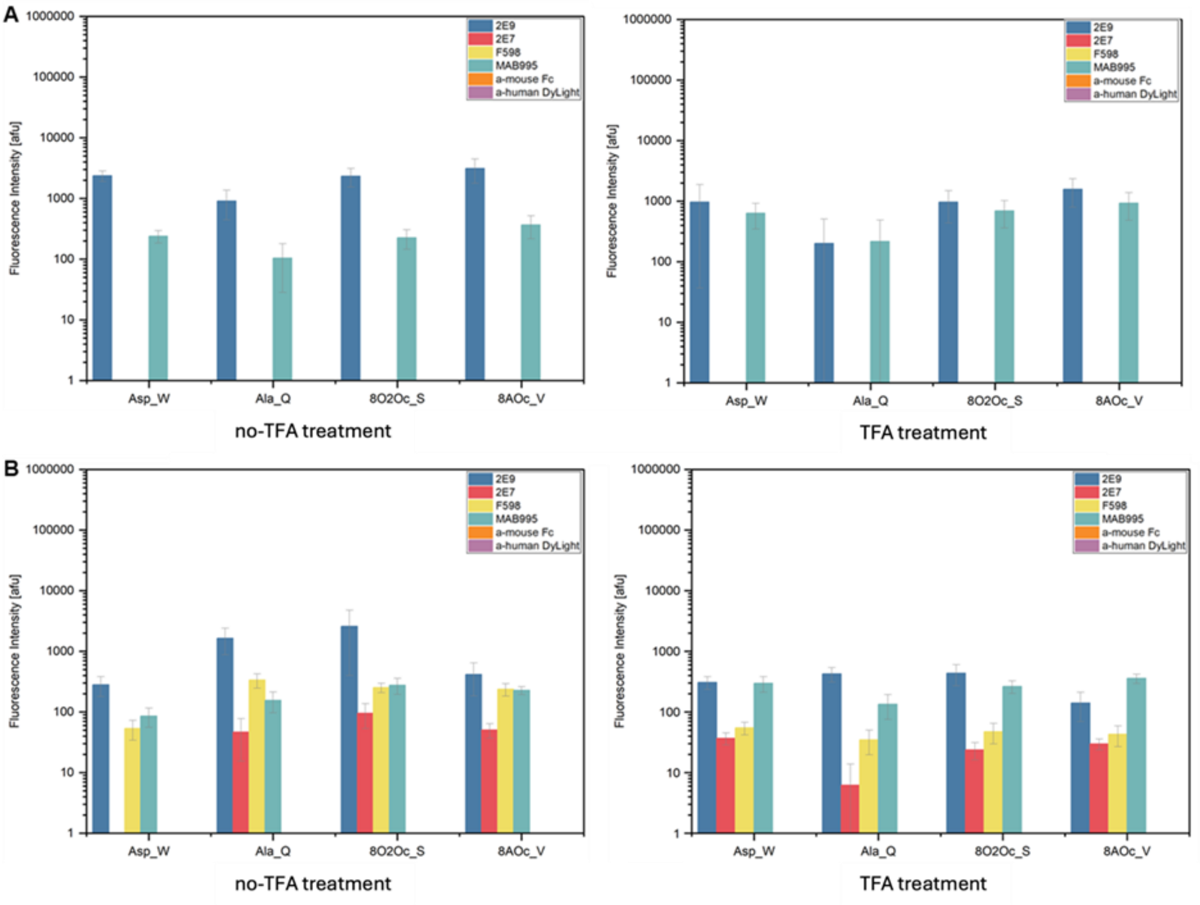

Figure S4: Fluorescence staining intensities of A) PEGMA/MMA-β-Ala-X-β-Ala-dimer **4** functionalized arrays and B) PEGMA/MMA-β-Ala-X-β-Ala-dimer **5** functionalized arrays with three anti-PGN and one anti-PNAG antibodies with and without TFA-treatment. Data shown as median ± SD as error bars, n = 125.

iv. PEGMA/MMA β-Ala-X-β-Ala-Pra (GlcNAc) ***6****-NHAc*

In this surface functionalization study (**Figure S5**), only the F598 antibody demonstrated significant binding, which is expected given that GlcNAc is a glycan building block present in mammalian cells. The other antibodies showed lower intensities, comparable to background levels. Notably, the binding intensities for the F598 antibody remained consistent between the arrays with and without TFA treatment. This consistency is attributed to the

attachment of sugars *via* CuAAC, which ensures stability and prevents hydrolytic cleavage during TFA treatment, a phenomenon observed in the D-lactoyl moiety of MurNAc.

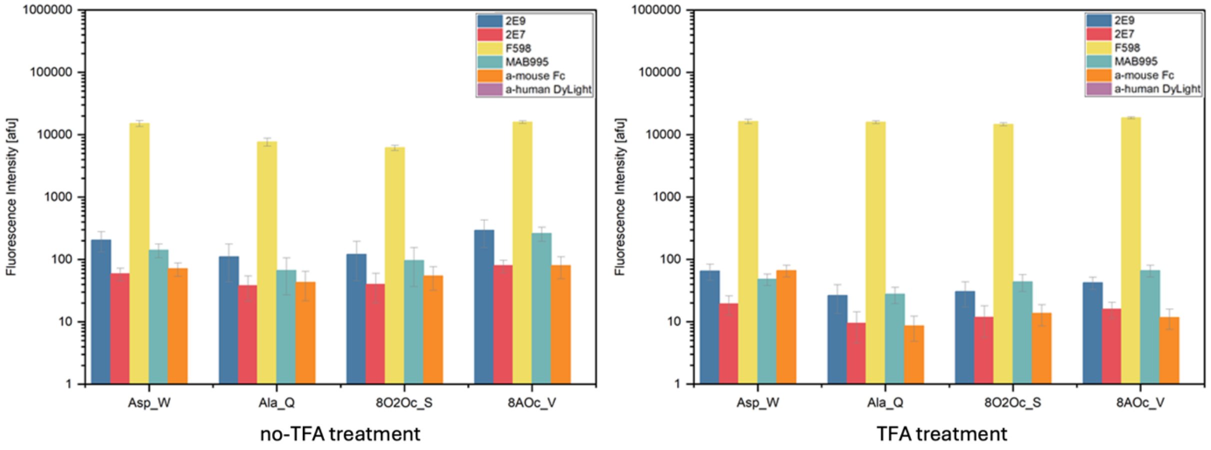

Figure S5: Fluorescence staining intensities of PEGMA/MMA β-Ala-X-β-Ala-Pra (GlcNAc **6**)-NHAc functionalized arrays with three anti-PGN and one anti-PNAG antibodies with and without TFA-treatment. Data shown as median ± SD as error bars, n = 125.

Summary of assumed epitopes recognized by mAbs

The data presented in **Figures S2-5** was summarized showing the recognized/bound structures for each mAb. Each mAb bound different epitopes with different binding strengths, categorized as strong, moderate, or weak. However, no comparisons were made between the individual antibodies on the same structures, due to individual differences in affinity/avidity and concentration. The relatively low intensities obtained for mAbs 2E9, 2E7, and MAB995 on the GlcNAc **6** arrays were likely due to the presence of background fluorescence, while the intensities observed for F598 were more pronounced (**Figure S6**).

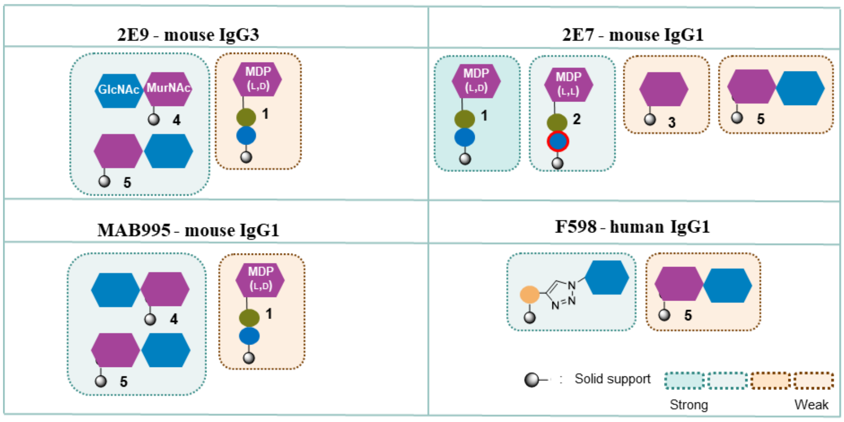

Figure S6: Structures recognized by three anti-PGN and one anti-PNAG mAbs.

I. Optimization of LIFT conditions

For the successful generation of peptidoglycan microarrays with high spot density (Pitch: 150 µm, 4444 spots/cm²), several parameters had to be tested and optimized as reported in the literature.[2] The pipeline previously reported for optimizing the 20 naturally occurring amino acids (L-AAs on PEPperPRINT slides) was implemented and different solid supports with varied functionalization were prepared via amide bond formation.

The required donor glass slides bearing the respective AAs were prepared using 10% AA (w/w), unless stated otherwise in **Table S2**. The transfer of each AA to the functionalized acceptor glass slide was achieved thrice at defined positions by varying the laser power and duration in a range (50–120 mW and 2–12 ms), while the coupling was achieved at 95 °C for 10 min, from which we extracted the optimal experimental parameters.

Screening of the generated arrays was achieved via fluorescence labeling. Two different labelling approaches were implemented: A. direct dye labeling with and without side chain deprotection with DyLight 633 *N*-hydroxy-succinimide ester and B. indirect biotin-streptavidin labeling with and without side chain deprotection. Each spot was analyzed in terms of homogeneity and size resulting in the following lasing parameters (**Table S2**).

Table S2: Peptide synthesis parameters 4444 spots/cm^2 (150 µm pitch). Laser transfer parameters for AA donor slides with a 10% AA concentration. Donor slides containing a 20% AA concentration are highlighted in green. Non-pre-activated AAs are indicated with (*).

AA	Given Symbols	Lasing Duration [ms]	Lasing Power [mw]	Coupling cycles
L -Glu	E	11	60 (40%)	3
D-isoGlu*	N	12	80 (48%)	3
L-isoGln*	P	12	100 (56%)	3
D-isoGln*	K	12	100 (56%)	3
L-Lys	I	11	80 (48%)	3
D-Lys*	L	11	80 (48%)	3
L-Arg	R	12	120 (64%)	4
L-Cit	C	9	80 (48%)	3
L-Hrs*	H	10	120 (64%)	3
L Gly	G	7	60 (40%)	3
L-Orn*	M	12	80 (48%)	3
L-Ala	Q	10	60 (40%)	3
D-Ala*	A	11	60 (40%)	3
L-Ser	F	9	80 (48%)	3
D-Ser*	D	9	80 (48%)	3
L-Asp	W	10	60 (40%)	3
8Aoc *****	V	8	120 (64%)	3
$8O_2Oc$	S	9	100 (56%)	3
L-Pra*	Y	7.5	80 (48%)	3

J. Generation & screening of combinatorial PGN arrays via mAbs

The PGN arrays were generated as explained in *Section G* and *Section I.*

Structures of stem peptides generated via LIFT:

Table S3: Overview of the 216 synthesized stem pentapeptides and the 27 smaller sequences found in the interpeptide bridge of PGNs, from the N→C terminus. Where W: L-Asp is the pre-patterning of the acceptor slide, A: D-Ala, C: L-Cit,, D: D-Ser, E: L-Glu, F: L-Ser, G: L-Gly, H: L-Hsr, I: D-Lys, K: D-iGln, L: L-Lys, M: L-Orn, N: D-iGlu, P: L-iGln, Q: L-Ala and R: L-Arg. Sequences found in Gram (+) highlighted in green, Gram (-) in blue, control (L-iGln, in position 2) no highlighted, and 27 interpeptide bridge sequences in orange.

GKLAAW	GNLAAW	GPLAAW	FKLAAW	FNLAAW	FPLAAW	QKLAAW	QNLAAW	QPLAAW
GKLADW	GNLADW	GPLADW	FKLADW	FNLADW	FPLADW	QKLADW	QNLADW	QPLADW
GKLAGW	GNLAGW	GPLAGW	FKLAGW	FNLAGW	FPLAGW	QKLAGW	QNLAGW	QPLAGW
GKMAAW	GNMAAW	GPMAAW	FKMAAW	FNMAAW	FPMAAW	QKMAAW	QNMAAW	QPMAAW
GKMADW	GNMADW	GPMADW	FKMADW	FNMADW	FPMADW	QKMADW	QNMADW	QPMADW
GKMAGW	GNMAGW	GPMAGW	FKMAGW	FNMAGW	FPMAGW	QKMAGW	QNMAGW	QPMAGW
GKHAAW	GNHAAW	GPHAAW	FKHAAW	FNHAAW	FPHAAW	QKHAAW	QNHAAW	QPHAAW
GKHADW	GNHADW	GPHADW	FKHADW	FNHADW	FPHADW	QKHADW	QNHADW	QPHADW
GKHAGW	GNHAGW	GPHAGW	FKHAGW	FNHAGW	FPHAGW	QKHAGW	QNHAGW	QPHAGW
GKQAAW	GNQAAW	GPQAAW	FKQAAW	FNQAAW	FPQAAW	QKQAAW	QNQAAW	QPQAAW
GKQADW	GNQADW	GPQADW	FKQADW	FNQADW	FPQADW	QKQADW	QNQADW	QPQADW
GKQAGW	GNQAGW	GPQAGW	FKQAGW	FNQAGW	FPQAGW	QKQAGW	QNQAGW	QPQAGW
GKEAAW	GNEAAW	GPEAAW	FKEAAW	FNEAAW	FPEAAW	QKEAAW	QNEAAW	QPEAAW
GKEADW	GNEADW	GPEADW	FKEADW	FNEADW	FPEADW	QKEADW	QNEADW	QPEADW
GKEAGW	GNEAGW	GPEAGW	FKEAGW	FNEAGW	FPEAGW	QKEAGW	QNEAGW	QPEAGW
GKIAAW	GNIAAW	GPIAAW	FKIAAW	FNIAAW	FPIAAW	QKIAAW	QNIAAW	QPIAAW
GKIADW	GNIADW	GPIADW	FKIADW	FNIADW	FPIADW	QKIADW	QNIADW	QPIADW
GKIAGW	GNIAGW	GPIAGW	FKIAGW	FNIAGW	FPIAGW	QKIAGW	QNIAGW	QPIAGW
GKRAAW	GNRAAW	GPRAAW	FKRAAW	FNRAAW	FPRAAW	QKRAAW	QNRAAW	QPRAAW
GKRADW	GNRADW	GPRADW	FKRADW	FNRADW	FPRADW	QKRADW	QNRADW	QPRADW
GKRAGW	GNRAGW	GPRAGW	FKRAGW	FNRAGW	FPRAGW	QKRAGW	QNRAGW	QPRAGW
GKCAAW	GNCAAW	GPCAAW	FKCAAW	FNCAAW	FPCAAW	QKCAAW	QNCAAW	QPCAAW

GKCADW	GNCADW	GPCADW	FKCADW	FNCADW	FPCADW	QKCADW	QNCADW	QPCADW
GKCAGW	GNCAGW	GPCAGW	FKCAGW	FNCAGW	FPCAGW	QKCAGW	QNCAGW	QPCAGW
KLAAW	NLAAW	PLAAW	KGAAW	NGAAW	PGAAW	LAAW	GAAW	AAW
W	AW	AAW	AAW	GW	GGW	GGGW	GGGGW	GGGGGW
W	GGGGGW	GGGGGW	GGGGGW	GGGGGW	AAW	AAW	AAW	AAW

Analyzed fluorescence scan results of the generated PGN arrays bearing the corresponding glycan moiety after staining with the respective anti-PGN mAbs with brief explanation of their preference towards structures found in Gram-positive and Gram-negative bacteria (**Figure S7**).

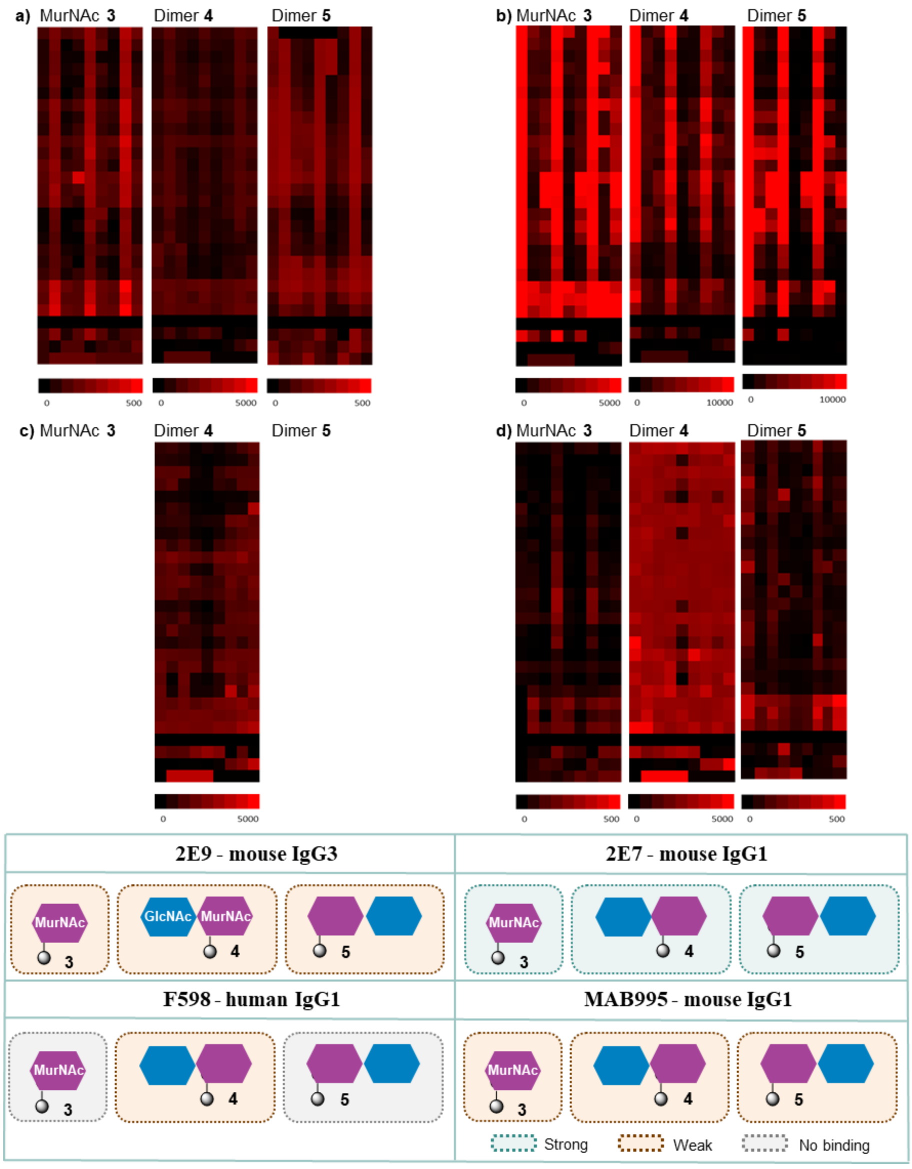

Figure S7: Heat map of the synthesized arrays containing 216 stem-pentapeptides and 27 shorter variations bearing the respective glycan moieties **3-5**, incubated with a) mouse anti-PGN monoclonal 2E9; b) mouse anti-PGN monoclonal 2E7; c) human anti-PNAG monoclonal monoclonal F598; and d) mouse anti-PGN monoclonal MAB995. Detection was achieved with

goat anti-mouse IgG polyclonal and goad anti-human IgG polyclonal respectively. Each array contained three copies of the corresponding pattern, n = 3. Data shown as median (of median IgG value). Scanning parameters: Wavelength 635 nm, PMT gain 600, laser power 33 %, pixel size 5 µm. Spot pitch is 150 µm. Summary of structural recognition on the generated arrays.

K. Microarray with PGN oligosaccharide fragments

Last, we performed additional experiments, in respect to the mAbs (2E9, 2E7, F598, MAB995) immobilizing ten different PGN oligosaccharide fragments up to hexamers (without the stem peptide amino acids, but with free -COOH at the MurNAc moieties) bearing a pentamino-linker. The structures (**Figure S8**) were previously synthesized by us using automated glycan assembly (AGA),[4] and we printed them on commercially functionalized *N*-hydroxysuccinimide (NHS) ester-activated surfaces. The immobilization of the oligosaccharides (0.1 mM glycan in 50 mM phosphate-buffered saline, pH 8.5) was accomplished using a piezoelectric microarray spotting device (S12 Scienion), which generated 64 identical array copies on each surface. Coupling of the transferred structures was achieved in a humid chamber at room temperature overnight, followed by quenching of the remaining unreacted NHS-esters with ethanolamine (100 mM) in sodium phosphate buffer for 1 h at room temperature. The generated slides were washed with water, dried in a jet of air and then subjected for incubation with the four monoclonal antibodies 2E9, 2E7, MAB995, and F598. Interestingly, the fluorescence analysis (**Figure S9**) shows low (but detectable) binding of 2E9 to all oligosaccharide structures. Specifically, we found that the binding of 2E7 is dependent on the presence of amino acid residues from stem peptide, and thus no binding was detected for the immobilized oligosaccharide fragments. In contrast, F598 was binding exclusively to the **GlcNAc 6** monomer, confirming our previous results. However, it is important to acknowledge that the obtained results on these arrays may not be directly comparable with our other microarray results, due to different surface functionalizations and manufacturing processes. Most importantly, the attachment of the PGN fragments between the two approaches differs: In the case of the PGN fragments generated with our laser printing technology, the oligosaccharides are attached through an amide bond, formed with the free carboxylic acid of the MurNAc building blocks. In contrast, the attachment of the 10 PGN oligosaccharide fragments without the peptide stem was achieved by the amino-pentanol linker attached to the anomeric position of the oligosaccharide, causing free carboxylic acids to be present on all MurNAc moieties. During the incubation of the arrays with mAbs in staining buffer (10% Rockland blocking buffer in PBS-T, pH: 7.4), the free carboxylic acid of the lactoyl moiety on the 10 PGN oligosaccharide fragments undergoes deprotonation, acquiring a negative charge. This will likely affect the binding of the mAbs, particularly the binding of MAB995, which should be further studied in the future.

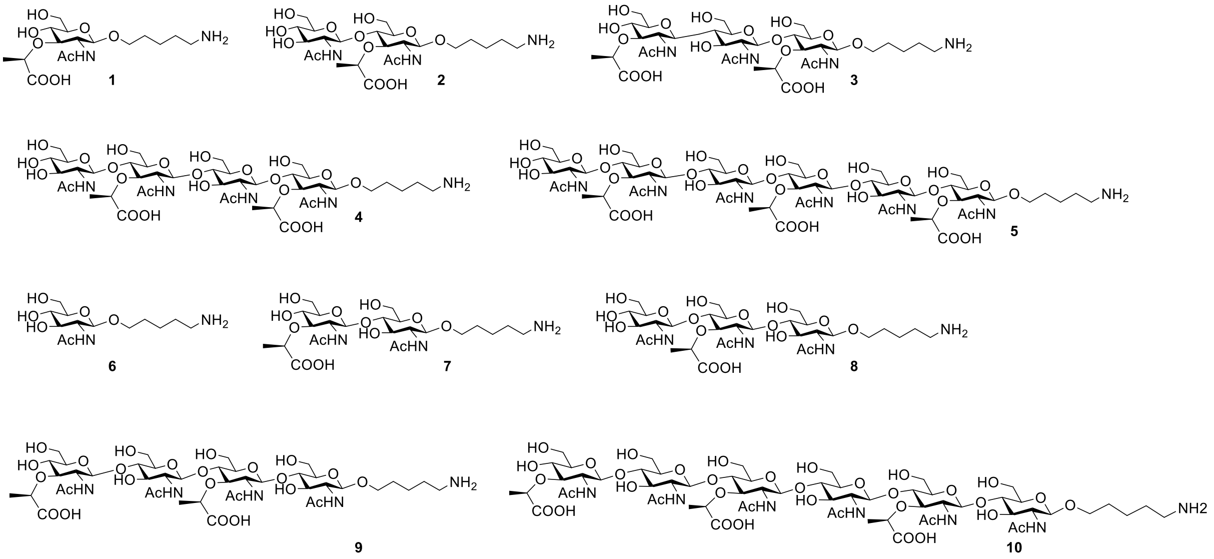

Figure S8. PGN oligosaccharide fragments synthesized by AGA.[4]

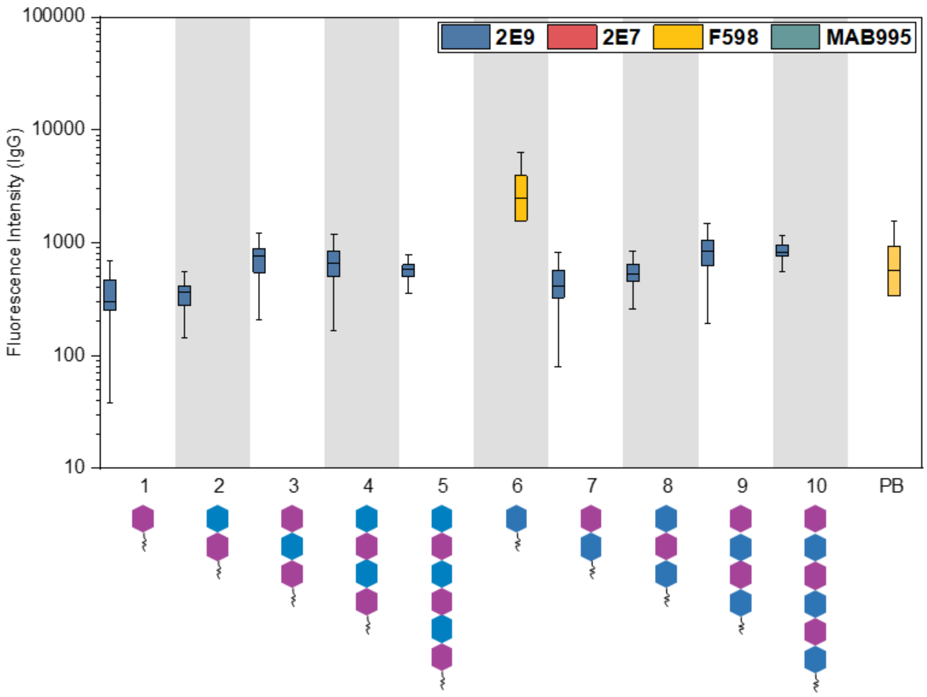

Figure S9. Analysis of the fluorescence intensities of 10 oligosaccharide PGN fragments after incubation with mouse anti-PGN monoclonal 2E9, mouse anti-PGN monoclonal 2E7, mouse anti-PGN monoclonal MAB995, and human anti-PNAG monoclonal F598. Detection was achieved with goat anti-mouse IgG polyclonal and goat anti-human IgG polyclonal antibodies respectively. The fluorescence intensity of each interaction was calculated as the median of 6 spots. Box plots (center line, median; box limits, upper and lower quartiles; whiskers, outermost data point that falls within 1.5 × interquartile range) were calculated from 125 spots.

Fluorescence scan images of screened arrays for each mAb are shown after incubation with the corresponding fluorescently labeled secondary antibodies (**Figure S10**).

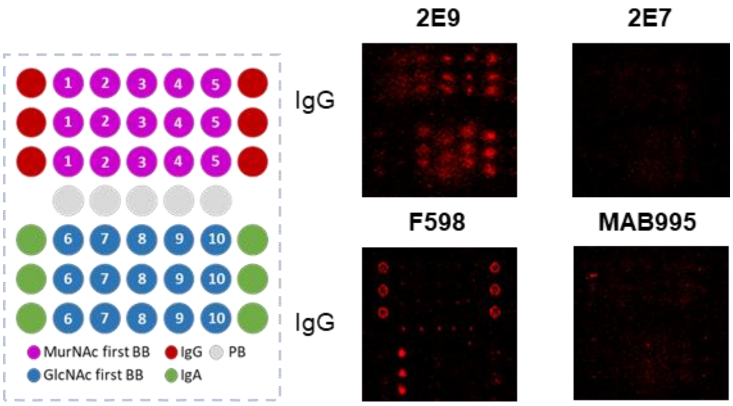

Figure S10. Layout and fluorescence scan images of spotted PGN glycan fragment arrays. The surface functionalization with the fluorescence intensities of the secondary anti-mouse IgG and anti-human IgG were used as negative control, while polyclonal IgG and IgA antibodies and the phosphate spotting buffer (PB) were spotted as controls. Scanning parameters: Wavelength 635 nm, PMT gain 800, laser power 33 %, pixel size 5 μm.

L. Epidermolysis bullosa (EB) patient sample screening

Epidermolysis bullosa (EB) is the collective name for a group of rare genetic disorders of skin fragility, leading to blistering and erosion upon minor trauma or friction. The wounds of EB patients are highly colonized with *S. aureus*, being more exposed than healthy individuals to staphylococcal antigens. Previous studies have shown that EB patients have highly elevated levels of *S. aureus*-specific IgG1 and IgG4 antibodies in their plasma and blister fluids compared to healthy individuals. There are several types of EB, each caused by mutations in different genes responsible for skin integrity.[11,12] The most common type of EB is Simplex (EBS), which can be in the outer layer of the skin, mostly in the areas of palms and feet. In this case, blisters are produced by heat or friction, and they can easily be healed without scarring the skin. Junctional EB (JEB) is more severe with the blisters already appearing during infancy on the skin and mucous membranes, influencing the junction between the epidermis and dermis. In Dystrophic EB (DEB), blisters are formed in the sublamina densa, leading to deeper skin involvement and scarring, as well as malformations around the fingers and toes.

In this study, we screened with our generated arrays (*Section H*), the IgG reactivity in plasma samples donated by patients with EB from the Dutch Epidermolysis Bullosa Registry (DEBR) from the Netherlands, as well as plasma samples donated by healthy non-carriers (NC) of *S. aureus* (NC00-NC10) and healthy nasal carriers (C) of *S. aureus* (C11-C18).

From IgG analysis to MDP **1** arrays with EB patient samples (EB01, EB02, EB09, EB11, EB14, EB15, EB51, EB53, EB58, EB60), distinct patterns were observed. Notably, in EB01 lower fluorescence intensity was acquired, indicating a weaker immune reactivity. In contrast, samples from patients EB02, EB11, EB14, and EB15 showed intense IgG signals, suggesting a robust immune reactivity, potentially reflecting higher levels of MDP **1**-specific antibodies in these individuals. For EB51, the IgG reactivity was detected only on surfaces functionalized with $8O_2Oc$ and 8Aoc, implying a degree of specificity in antibody binding to these functional groups. Interestingly, healthy controls NC00, NC01, NC02, NC07 also showed binding to **MDP 1**, raising questions about possible baseline immune recognition of **MDP** or cross-reactivity potentially from another bacterial infection, which merits further investigation. In summary, 9 out of 13 patient samples showed **MDP 1** binding with relatively high intensities.

In our study (**Figure S11**), specific binding to MurNAc **3** was observed only in seven samples from patients with EB (EB01, EB11, EB14, EB15, EB51, EB55, EB60). In particular, among the samples, those from patients with junctional EB (JEB), EB01, EB15, and EB60, exhibited an IgG reactivity across all tested surface functionalizations. In contrast, the sample from a patient with epidermolysis bullosa simplex (EBS), EB11, which had non-chronic wounds, showed an IgG reactivity exclusively on the array prefunctionalized with aspartic acid (Asp). This indicates, that MurNAc might be a biomarker for *S. aureus* carriage or infection in the case of EB patient samples.

Interestingly, samples EB51 and EB55 did not display reactivity on the Asp surface, whereas responses were detected on arrays prefunctionalized with the other three amino acids. Lastly, sample EB14 exhibited IgG reactivity solely on surfaces functionalized with both aliphatic and non-aliphatic amino acids. These findings suggest that the specific surface functionalization plays a critical role in detecting IgG binding in different EB subtypes, highlighting the importance of tailored approaches for antibody screening in EB disease etiology. The intensities of the IgG binding on each surface are summarized below:

Asp: *EB60* > *EB01* > *EB11* > *EB15*

Ala: *EB60* > *EB01* > *EB51* > *EB15* > *EB55*

$8O_2Oc$: *EB60* > *EB01* > *EB51* > *EB15* > *EB14* > *EB55*

8Aoc: *EB60* > *EB01* > *EB51* > *EB15* > *EB14* > *EB55*

Additionally, we observed a high IgG reactivity among all samples towards the dimer **4** but not in EB55 which is intriguing. It is considered that dimer **4** is the smallest epitope found ubiquitously across samples, and given its small size and common presence, it is likely recognized by a wide range of antibodies due to its basic structural features, making it a common epitope.

On the GlcNAc **6** arrays, an IgG binding was observed in all samples apart from the EB01 sample, with intensities higher on healthy individuals than in patient samples. This can be explained due to the unique immune response in each individual or the different progression of the disease in comparison with the genetic factor and the immune history of this patient. In the case of samples NC08 and C14, unspecific IgG reactivity was detected across all structures, which may be attributed to hemolysis and/or contamination.

Finally, no binding was detected on the plain arrays bearing only the surface functionalization without any sugar moieties attached. In addition to the IgG analysis, IgA reactivities of all patient samples were screened in parallel (Fc specific secondary antibodies). However, the results from the IgA staining were not further investigated in this study (**Figure S12**).

Asp functionalization – IgG, shown in the main manuscript (**Figure 5**)

Ala functionalization - IgG

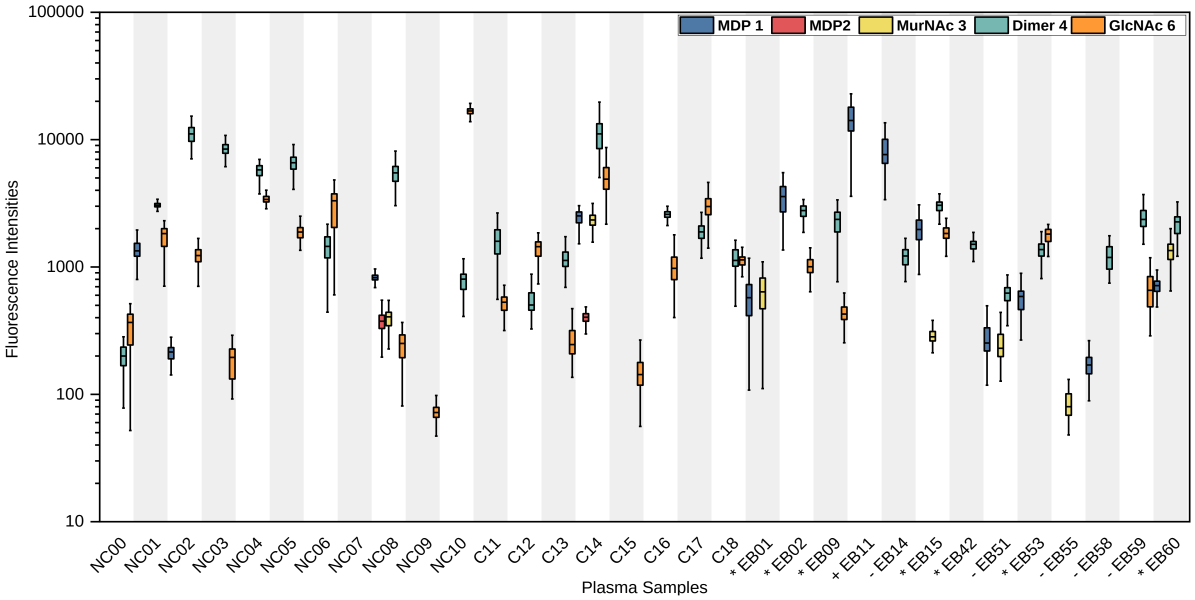

$8O_2Oc$ functionalization - IgG

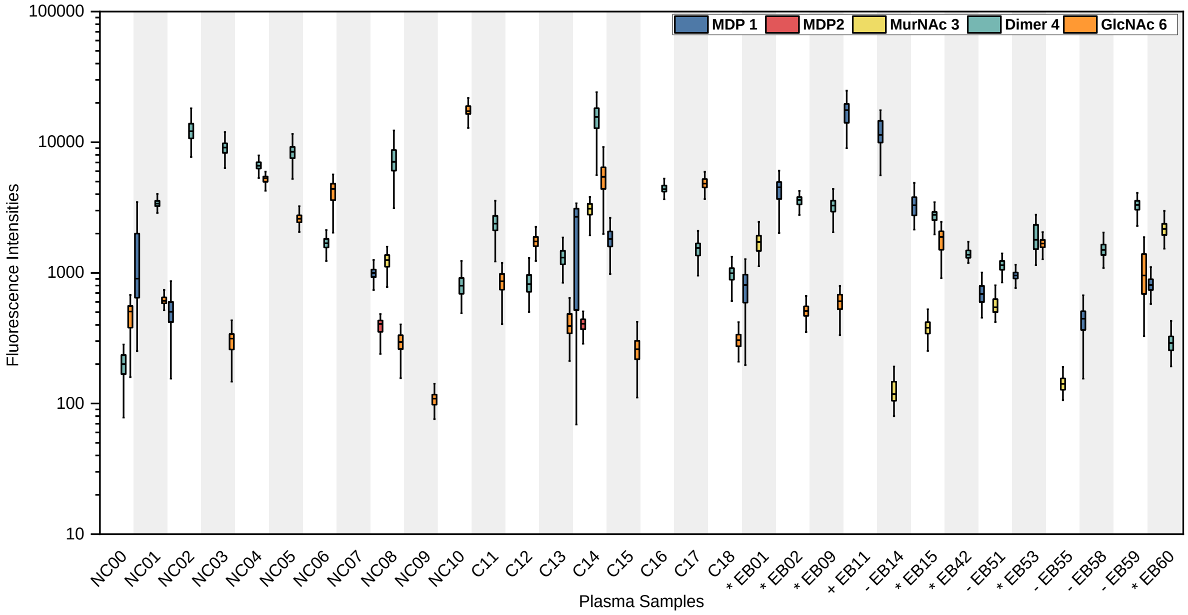

8Aoc functionalization - IgG

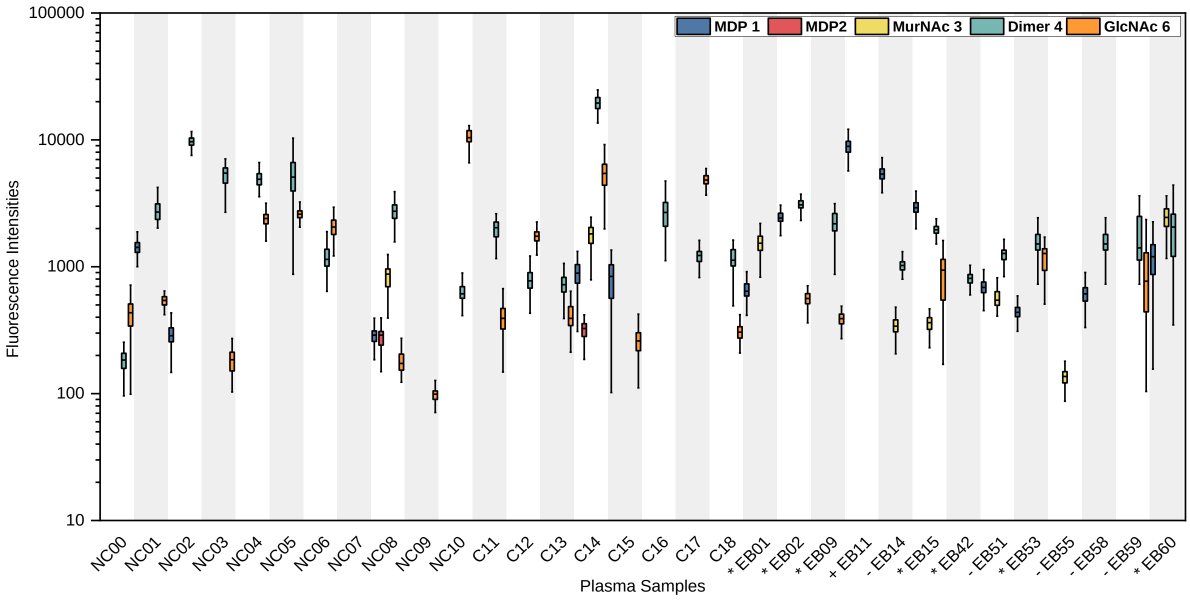

Figure S11. Detection of IgG reactivity in plasma from non-carriers (NC), carriers (C) of *S. aureus*, and EB patients on differently functionalized solid supports bearing L-Asp, L-Ala, $8O_2Oc$, 8Aoc. Analysis of median fluorescence intensity for each individual PGN-fragment detected for the respective patient sample. EB samples grouped as: JEB (*), DEB (-), EBS sample (+). Box plots (center line, median; box limits, upper and lower quartiles; whiskers, outermost data point that falls within 1.5 × interquartile range) were calculated from 125 spots.

Asp functionalization - IgA

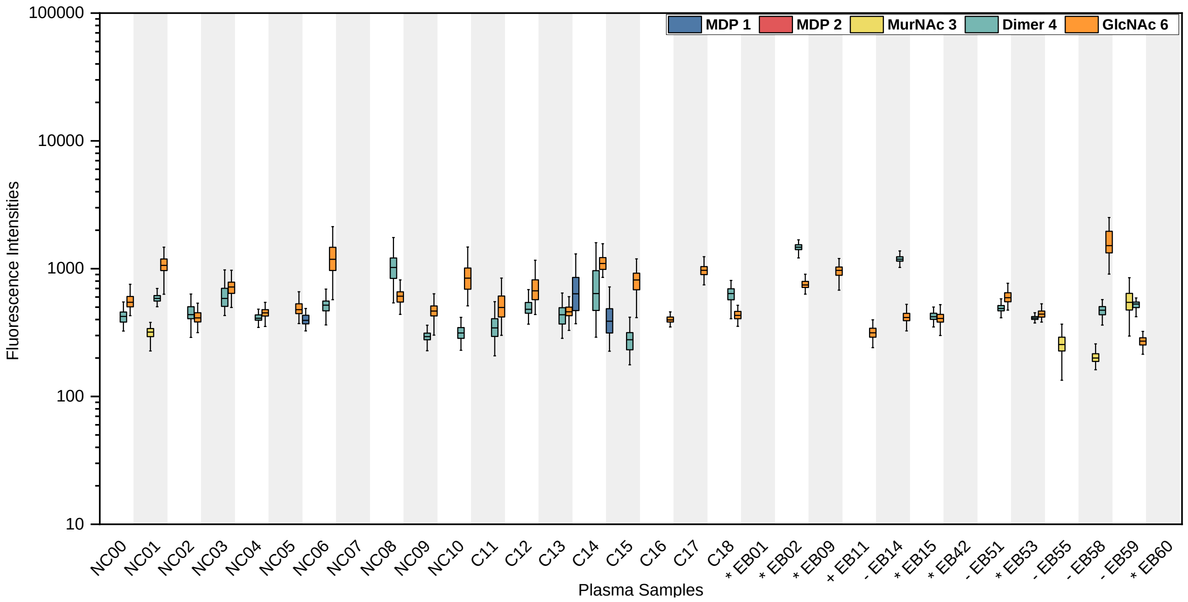

Ala functionalization - IgA

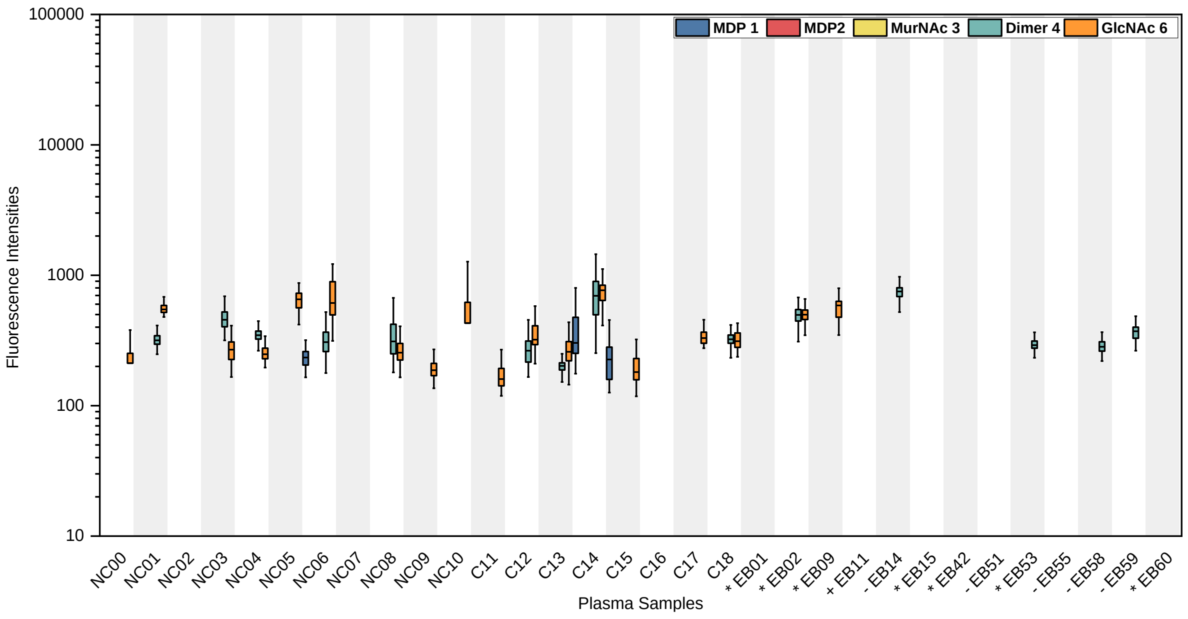

$8O_2Oc$ functionalization - IgA

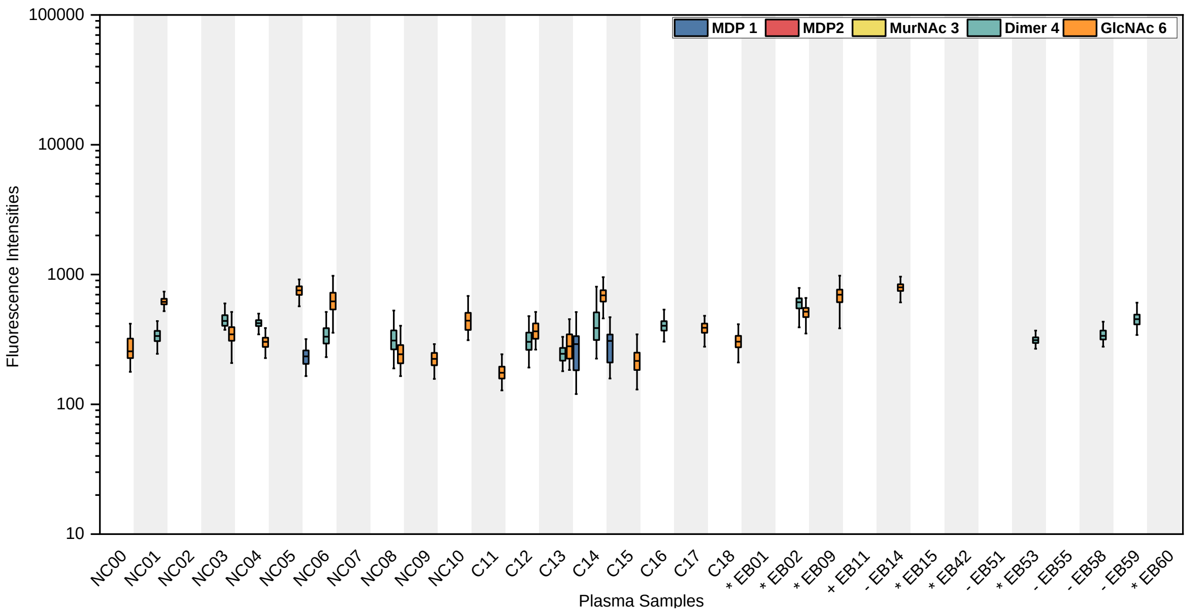

8Aoc functionalization - IgA

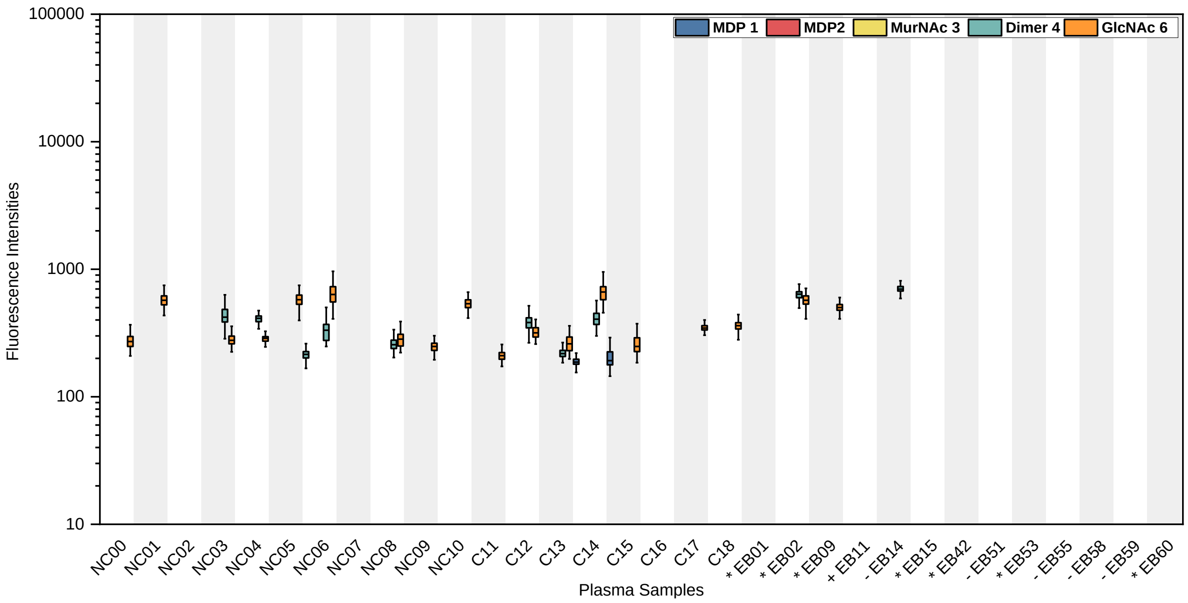

Figure S12. Detection of IgA reactivity in plasma from non-carriers (NC), carriers (C) of *S. aureus*, and EB patients on differently functionalized solid supports bearing L-Asp, L-Ala, 8O2Oc, 8Aoc. Analysis of median fluorescence intensity for each individual PGN-fragment detected for the respective patient sample. EB samples grouped as: JEB (*), DEB (-), EBS sample (+). Box plots (center line, median; box limits, upper and lower quartiles; whiskers, outermost data point that falls within 1.5 × interquartile range) were calculated from 125 spots.

Overview of human samples in respect to their IgG reactivity

Table S4: List of screened plasma samples. Eleven non-carrier (NC) samples, eight *S. aureus* carrier (C) samples of, and 13 samples of EB patients were screened.

No. Sample	Type	Category	1	2	3	4	6
**NC00**	-	-	√	×	×	√	√
**NC01**	-	-	√	×	×	√	√
**NC02**	-	-	√	×	×	√	√
**NC03**	-	-	×	×	×	√	○
**NC04**	-	-	×	×	×	√	√
**NC05**	-	-	×	×	×	√	√
**NC06**	-	-	×	×	×	√	√
**NC07**	-	-	○	×	×	×	×
**NC08**	-	-	√	√	√	√	√
**NC09**	-	-	×	×	×	×	○
**NC10**	-	-	×	×	√	×	√
**C11**	-	-	×	×	×	×	√
**C12**	-	-	×	×	×	√	√
**C13**	-	-	×	×	×	√	√
**C14**	-	-	√	√	√	√	√
**C15**	-	-	×	×	×	√	√
**C16**	-	-	×	×	×	√	√
**C17**	-	-	×	×	×	√	√
**C18**	-	-	×	×	×	√	√
**EB01**	JEB (*)	Chronic wounds	√	×	○	√	×
**EB02**	JEB (*)	Chronic wounds	√	×	×	√	√
**EB09**	JEB (*)	Chronic wounds	√	×	×	√	√

**EB11**	EBS (+)	non-Chronic wounds	√	×	○	√	√
**EB14**	DEB (-)	Chronic wounds	√	×	○	√	√
**EB15**	JEB (∗)	Chronic wounds	√	×	○	√	√
**EB42**	JEB (∗)	Chronic wounds	×	×	×	√	√
**EB51**	DEB (-)	Chronic wounds	√	×	○	√	○
**EB53**	JEB (∗)	non-Chronic wounds	√	×	×	√	√
**EB55**	DEB (-)	Chronic wounds	×	×	○	×	√
**EB58**	DEB (-)	Chronic wounds	√	×	×	√	○
**EB59**	DEB (-)	Chronic wounds	×	×	×	√	√
**EB60**	JEB (∗)	Chronic wounds	√	×	√	√	○

M. Fluorescence scan images of EB samples

In the following section the fluorescence scan images of all synthesized and screened arrays are shown after incubation of each structure with the corresponding fluorescently labeled secondary antibodies. The plain peptides as well as the surface functionalization were used as negative controls. Scanning parameters: Wavelength 635/532 nm, PMT gain 600, laser power 33 %, pixel size 5 µm, Spot pitch is 150 µm.

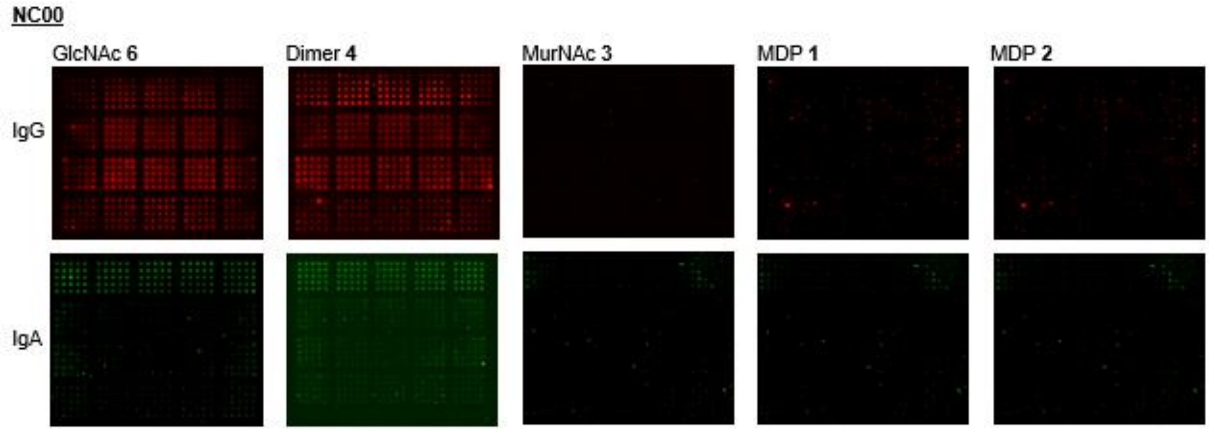

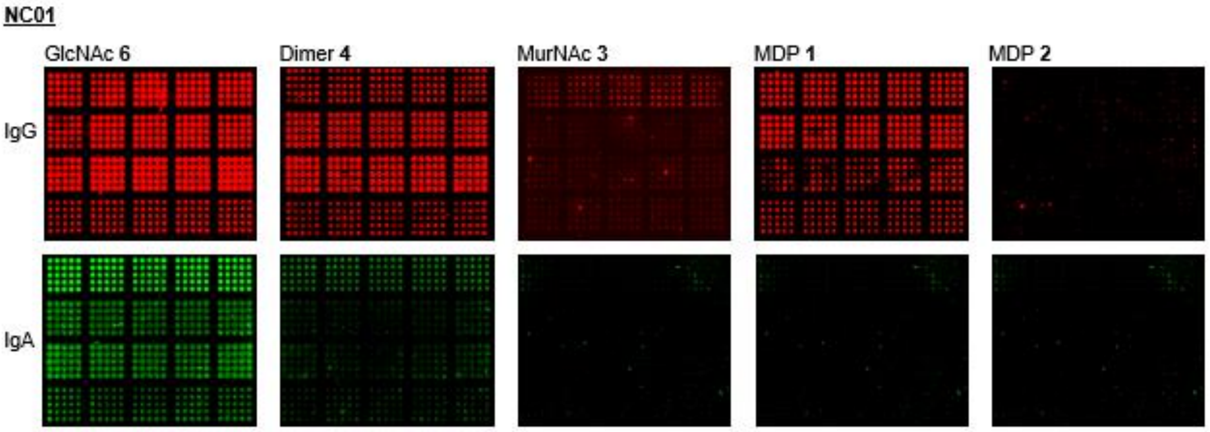

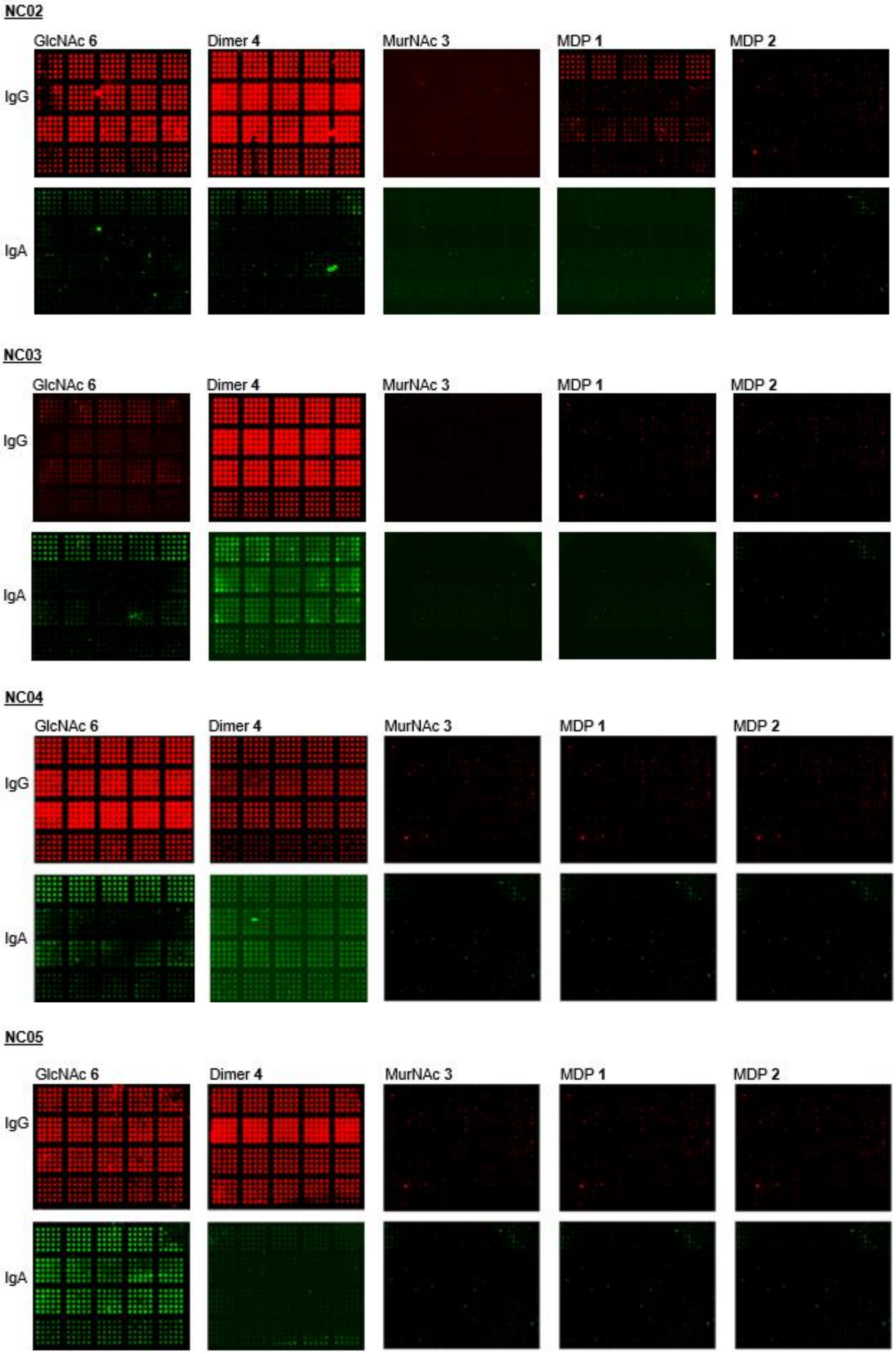
NC02
GlcNAc 6
Dimer 4
MurNAc 3
MDP 1
MDP 2
IgG
IgA
NC03
GlcNAc 6
Dimer 4
MurNAc 3
MDP 1
MDP 2
IgG
IgA
NC04
GlcNAc 6
Dimer 4
MurNAc 3
MDP 1
MDP 2
IgG
IgA
NC05
GlcNAc 6
Dimer 4
MurNAc 3
MDP 1
MDP 2
IgG
IgA

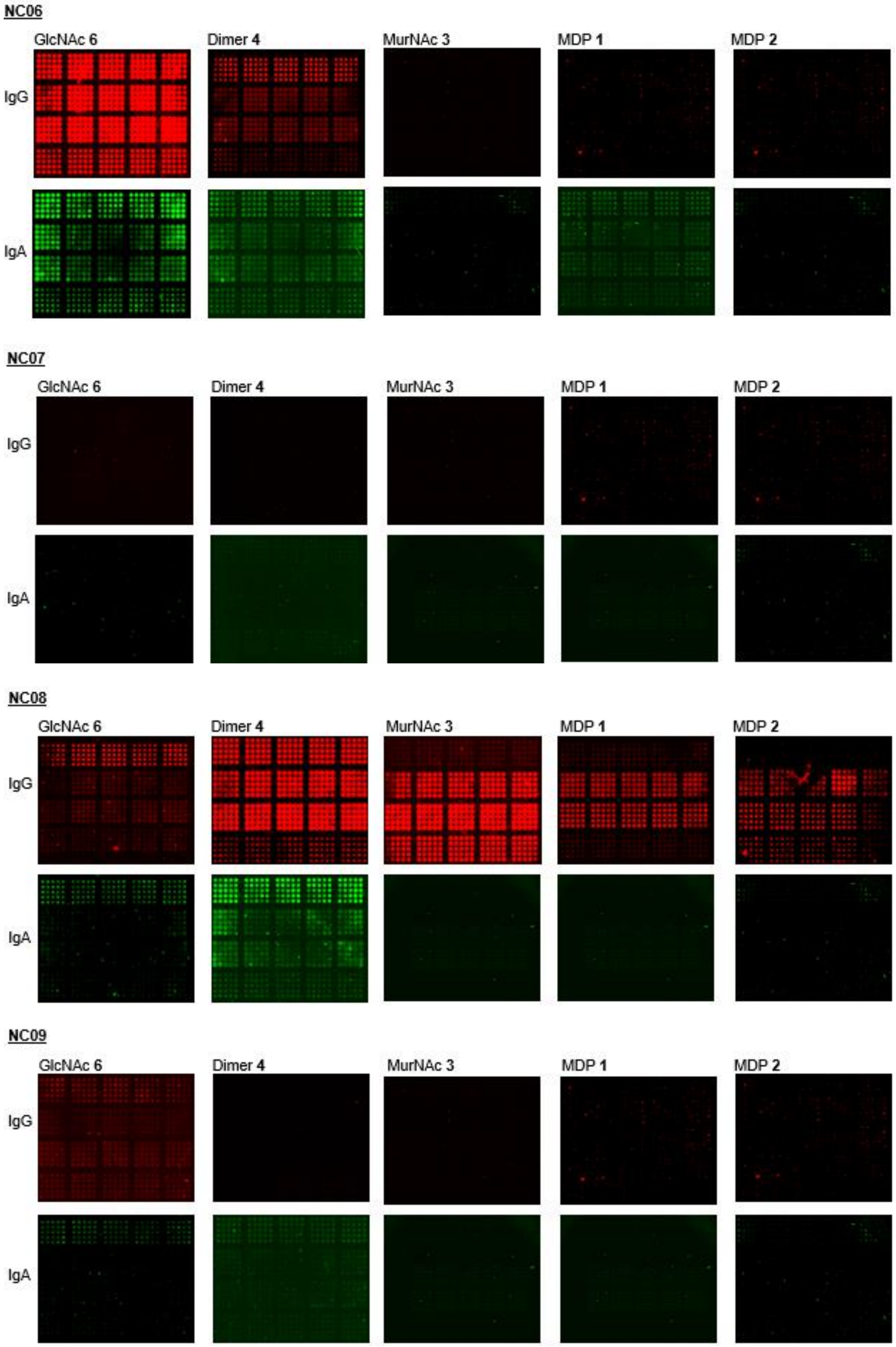
NC06
GlcNAc 6
Dimer 4
MurNAc 3
MDP 1
MDP 2
IgG
IgA
NC07
GlcNAc 6
Dimer 4
MurNAc 3
MDP 1
MDP 2
IgG
IgA
NC08
GlcNAc 6
Dimer 4
MurNAc 3
MDP 1
MDP 2
IgG
IgA
NC09
GlcNAc 6
Dimer 4
MurNAc 3
MDP 1
MDP 2
IgG
IgA

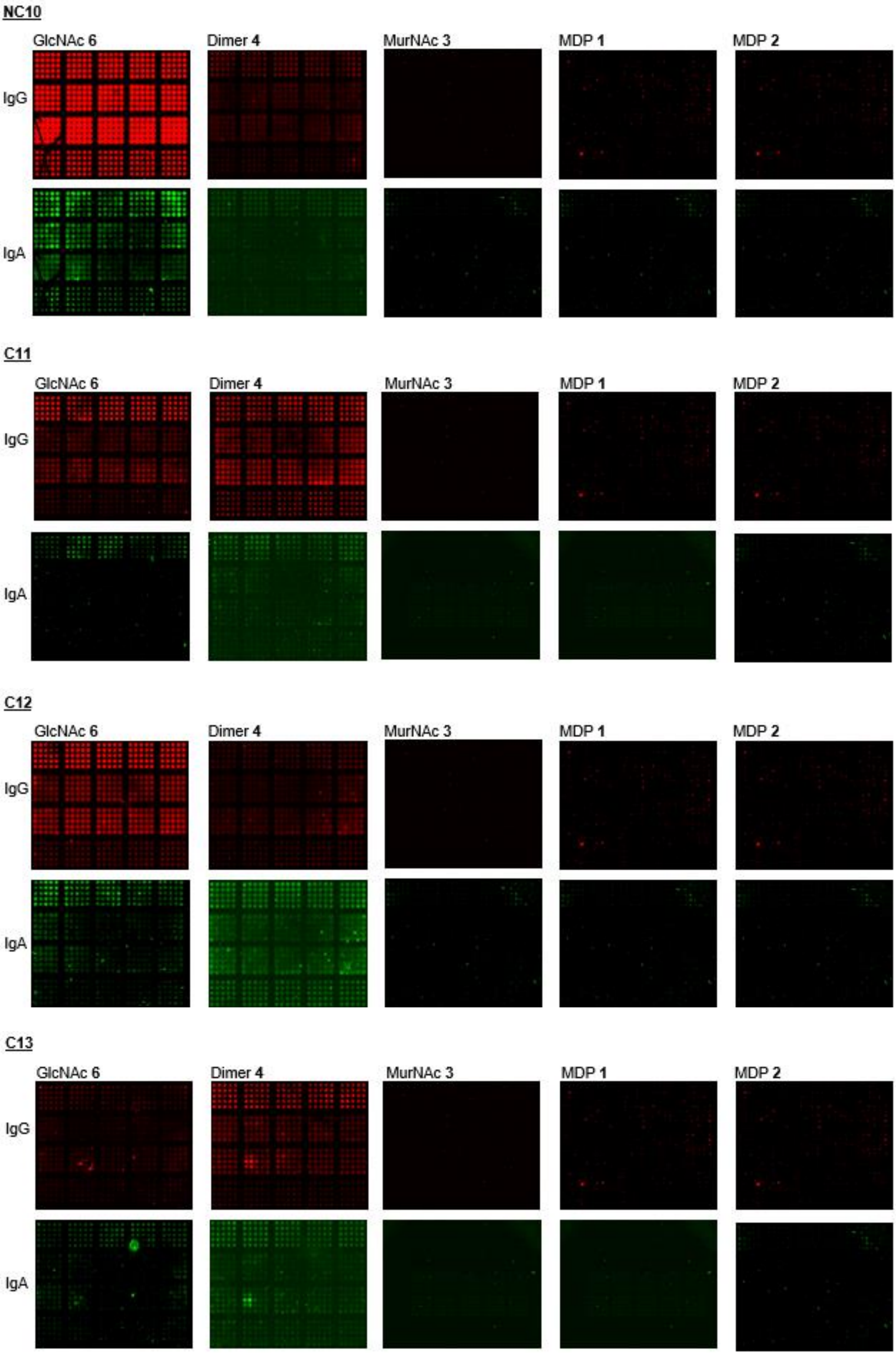
NC10
GlcNAc 6
Dimer 4
MurNAc 3
MDP 1
MDP 2
IgG
IgA
C11
GlcNAc 6
Dimer 4
MurNAc 3
MDP 1
MDP 2
IgG
IgA
C12
GlcNAc 6
Dimer 4
MurNAc 3
MDP 1
MDP 2
IgG
IgA
C13
GlcNAc 6
Dimer 4
MurNAc 3
MDP 1
MDP 2
IgG
IgA

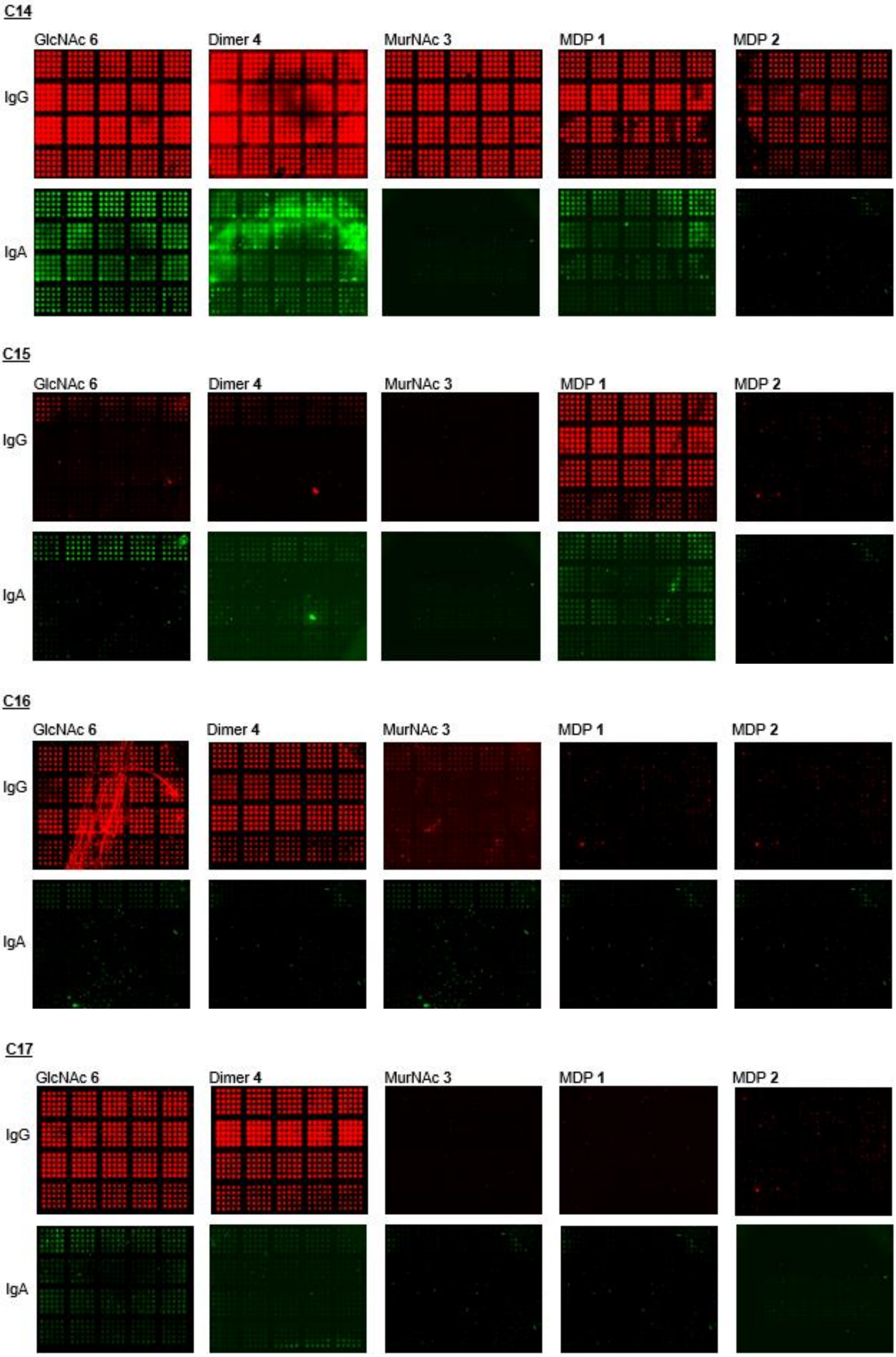
C14
GlcNAc 6
Dimer 4
MurNAc 3
MDP 1
MDP 2
IgG
IgA
C15
GlcNAc 6
Dimer 4
MurNAc 3
MDP 1
MDP 2
IgG
IgA
C16
GlcNAc 6
Dimer 4
MurNAc 3
MDP 1
MDP 2
IgG
IgA
C17
GlcNAc 6
Dimer 4
MurNAc 3
MDP 1
MDP 2
IgG
IgA

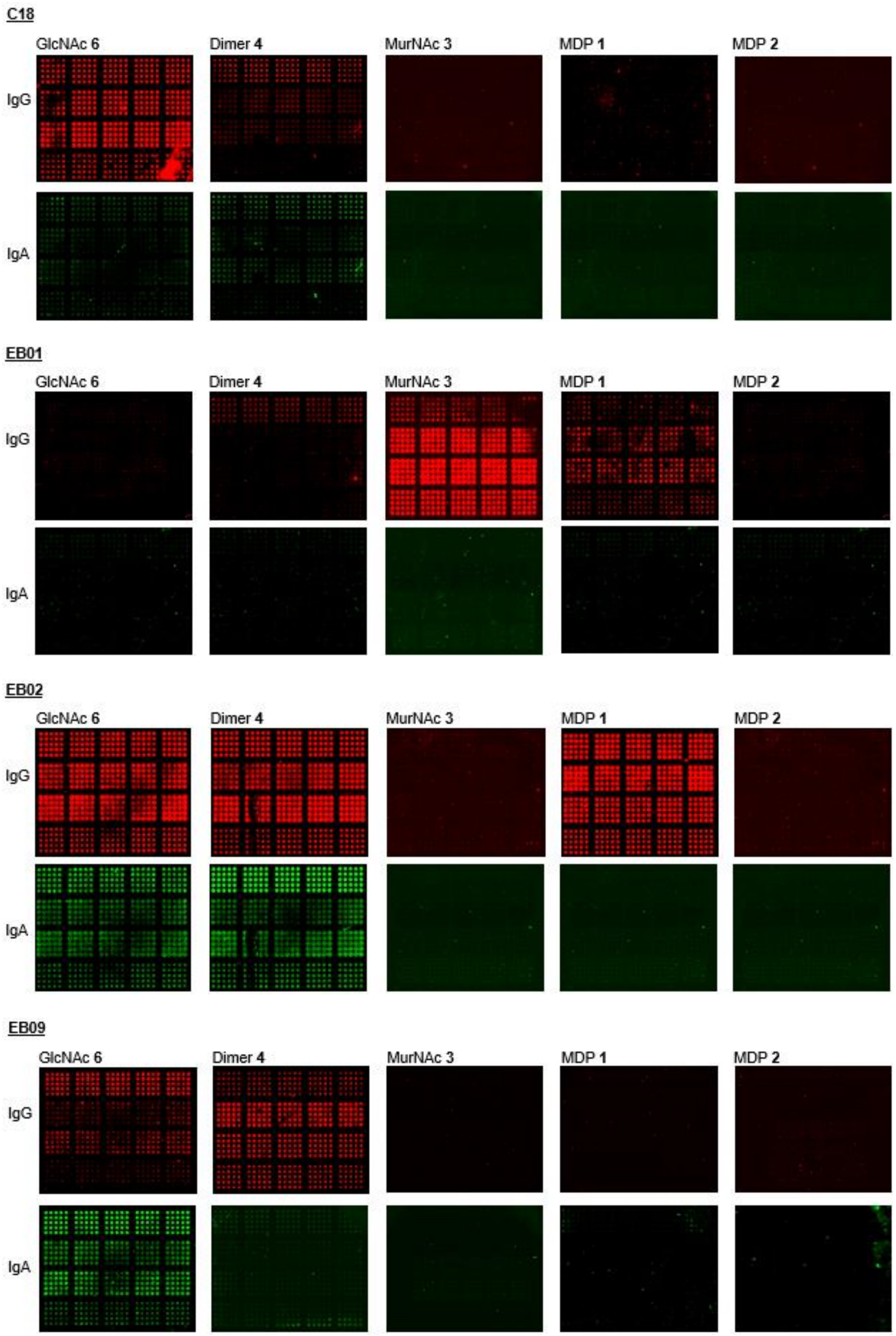
C18
GlcNAc 6
Dimer 4
MurNAc 3
MDP 1
MDP 2
IgG
IgA
EB01
GlcNAc 6
Dimer 4
MurNAc 3
MDP 1
MDP 2
IgG
IgA
EB02
GlcNAc 6
Dimer 4
MurNAc 3
MDP 1
MDP 2
IgG
IgA
EB09
GlcNAc 6
Dimer 4
MurNAc 3
MDP 1
MDP 2
IgG
IgA

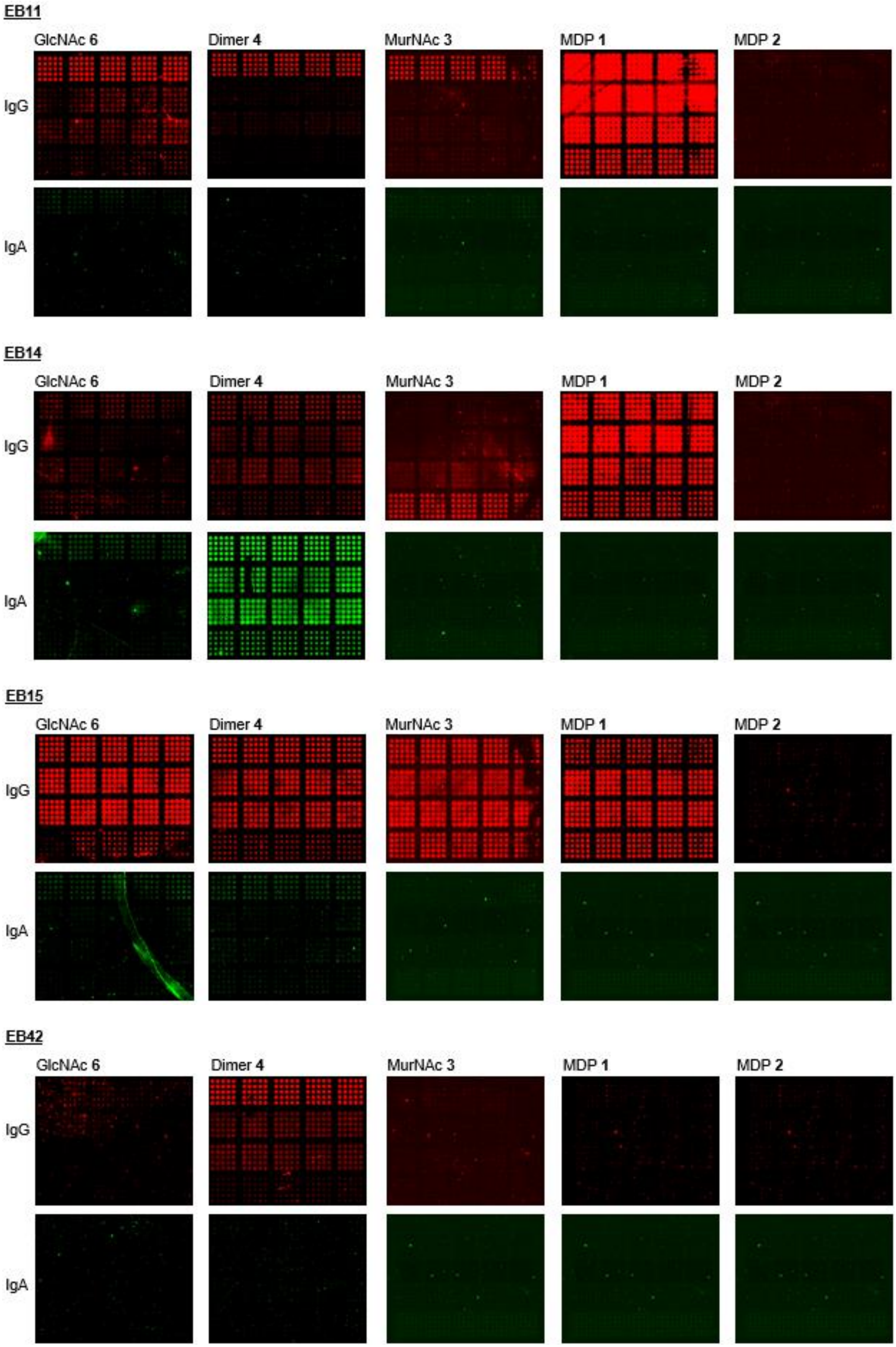
EB11
GlcNAc 6
Dimer 4
MurNAc 3
MDP 1
MDP 2
IgG
IgA
EB14
GlcNAc 6
Dimer 4
MurNAc 3
MDP 1
MDP 2
IgG
IgA
EB15
GlcNAc 6
Dimer 4
MurNAc 3
MDP 1
MDP 2
IgG
IgA
EB42
GlcNAc 6
Dimer 4
MurNAc 3
MDP 1
MDP 2
IgG
IgA

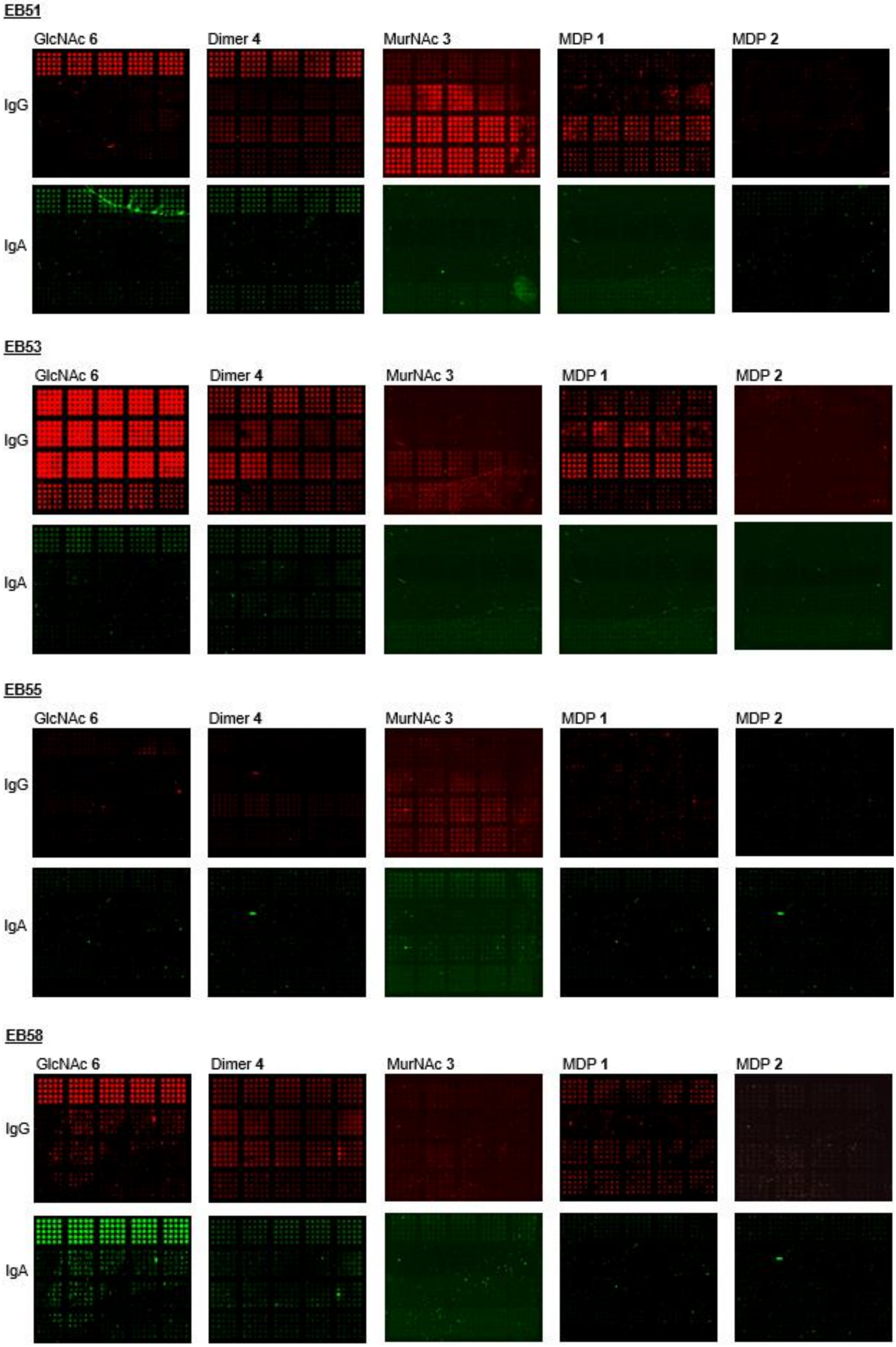
EB51
GlcNAc 6
Dimer 4
MurNAc 3
MDP 1
MDP 2
IgG
IgA
EB53
GlcNAc 6
Dimer 4
MurNAc 3
MDP 1
MDP 2
IgG
IgA
EB55
GlcNAc 6
Dimer 4
MurNAc 3
MDP 1
MDP 2
IgG
IgA
EB58
GlcNAc 6
Dimer 4
MurNAc 3
MDP 1
MDP 2
IgG
IgA

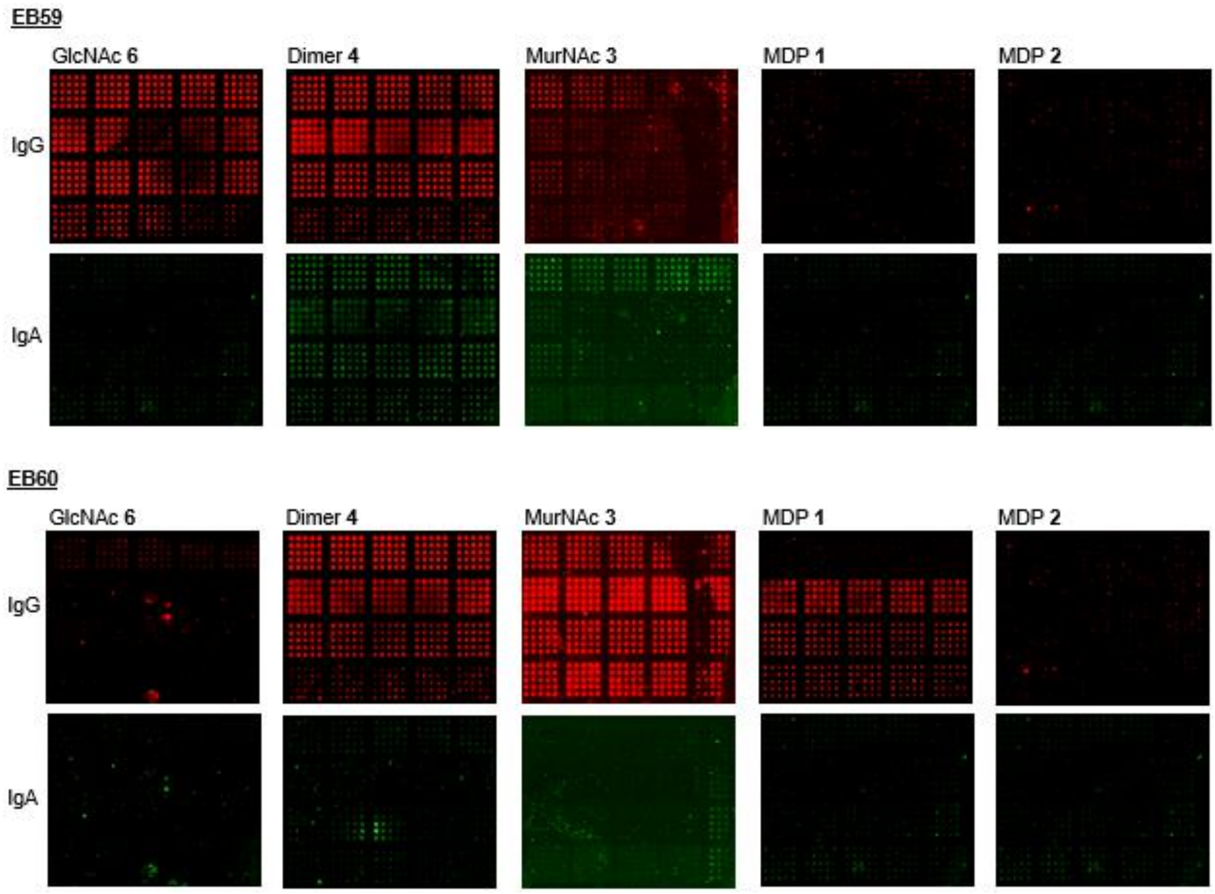

P. References

[1] W. Vollmer, D. Blanot, M. A. De Pedro, *FEMS Microbiol. Rev.* **2008**, *32*, 149–167.

[2] G. Paris, J. Heidepriem, A. Tsouka, Y. Liu, D. S. Mattes, S. Pinzón Martín, P. Dallabernardina, M. Mende, C. Lindner, R. Wawrzinek, C. Rademacher, P. H. Seeberger, F. Breitling, F. R. Bischoff, T. Wolf, F. F. Loeffler, *Adv. Mater.* **2022**, *34*, 2200359.

[3] J. Kandasamy, F. Schuhmacher, H. S. Hahm, J. C. Klein, P. H. Seeberger, *Chem. Commun.* **2014**, *50*, 1875–1877.

[4] P. Dallabernardina, V. Benazzi, J. D. Laman, P. H. Seeberger, F. F. Loeffler, *Org. Biomol. Chem.* **2021**, *19*, 9829–9832.

[5] A. Tsouka, K. Hoetzel, M. Mende, J. Heidepriem, G. Paris, S. Eickelmann, P. H. Seeberger, B. Lepenies, F. F. Loeffler, *Front. Chem.* **2021**, *9*, 931.

[6] M. P. H. and J. D. L. Ingrid A. Schrijver, Marjan van Meurs, Marie-Jose ́Melief, C. Wim Ang, Dragan Buljevac, Rivka Ravid, I. A. Schrijver, M. Van Meurs, M. J. Melief, C. W. Ang, D. Buljevac, R. Ravid, M. P. Hazenberg, J. D. Laman, *Brain* **2001**, *124*, 1544–1554.

[7] Z. Huang, J. Wang, X. Xu, H. Wang, Y. Qiao, W. C. Chu, S. Xu, L. Chai, F. Cottier, N. Pavelka, M. Oosting, L. A. B. Joosten, M. Netea, C. Y. L. Ng, K. P. Leong, P. Kundu, K.-P. Lam, S. Pettersson, Y. Wang, *Nat. Microbiol.* **2019**, *4*, 766–773.

[8] C. Soliman, A. K. Walduck, E. Yuriev, J. S. Richards, C. Cywes-Bentley, G. B. Pier, P. A. Ramsland, *J.*

Biol. Chem. **2018**, *293*, 5079–5089.

[9] L. de Vor, B. van Dijk, K. van Kessel, J. S. Kavanaugh, C. de Haas, P. C. Aerts, M. C. Viveen, E. C. Boel, A. C. Fluit, J. M. Kwiecinski, G. C. Krijger, R. M. Ramakers, F. J. Beekman, E. Dadachova, M. G. E. H. Lam, H. C. Vogely, B. C. H. van der Wal, J. A. G. van Strijp, A. R. Horswill, H. Weinans, S. H. M. Rooijakkers, *Elife* **2022**, 11:e67301.

[10] W. G. Branton, J. Q. Lu, M. G. Surette, R. A. Holt, J. Lind, J. D. Laman, C. Power, *Sci. Rep.* **2016**, *6*, 37344.

[11] F. Romero Pastrana, J. Neef, D. G. A. M. Koedijk, D. de Graaf, J. Duipmans, M. F. Jonkman, S. Engelmann, J. M. van Dijl, G. Buist, *Sci. Rep.* **2018**, *8*, 3234.

[12] M. M. van der Kooi-Pol, C. P. de Vogel, G. N. Westerhout-Pluister, Y. K. Veenstra-Kyuchukova, J. C. Duipmans, C. Glasner, G. Buist, G. S. Elsinga, H. Westra, H. P. J. Bonarius, H. Groen, W. J. B. van Wamel, H. Grundmann, M. F. Jonkman, J. M. van Dijl, *J. Invest. Dermatol.* **2013**, *133*, 847–850.

Ethical Approval

Sera from EB patients were collected with approval of the Medical Ethics Committee of the University Medical Center Groningen (approval no. Nl2747104209), and sera from healthy human volunteers were collected with approval of the Independent Ethics Committee of the Foundation 'Evaluation of Ethics in Biomedical Research' (Assen, the Netherlands). All patients and healthy volunteers provided written informed consent in this study.[12] The study was performed with adherence to the Helsinki Guidelines and local regulations. Whole blood samples were processed for further analyses immediately after collection. The donated blood was diluted 1:1 in Hanks' Balanced Salt Solution (HBSS, Gibco). Plasma was obtained after separation from blood cells using Ficoll-Paque PLUS (GE Healthcare) according to manufacturer's instructions. The collected human plasma was stored at -30°C.[12]

Author contribution

All authors have given approval to the final version of the manuscript.
